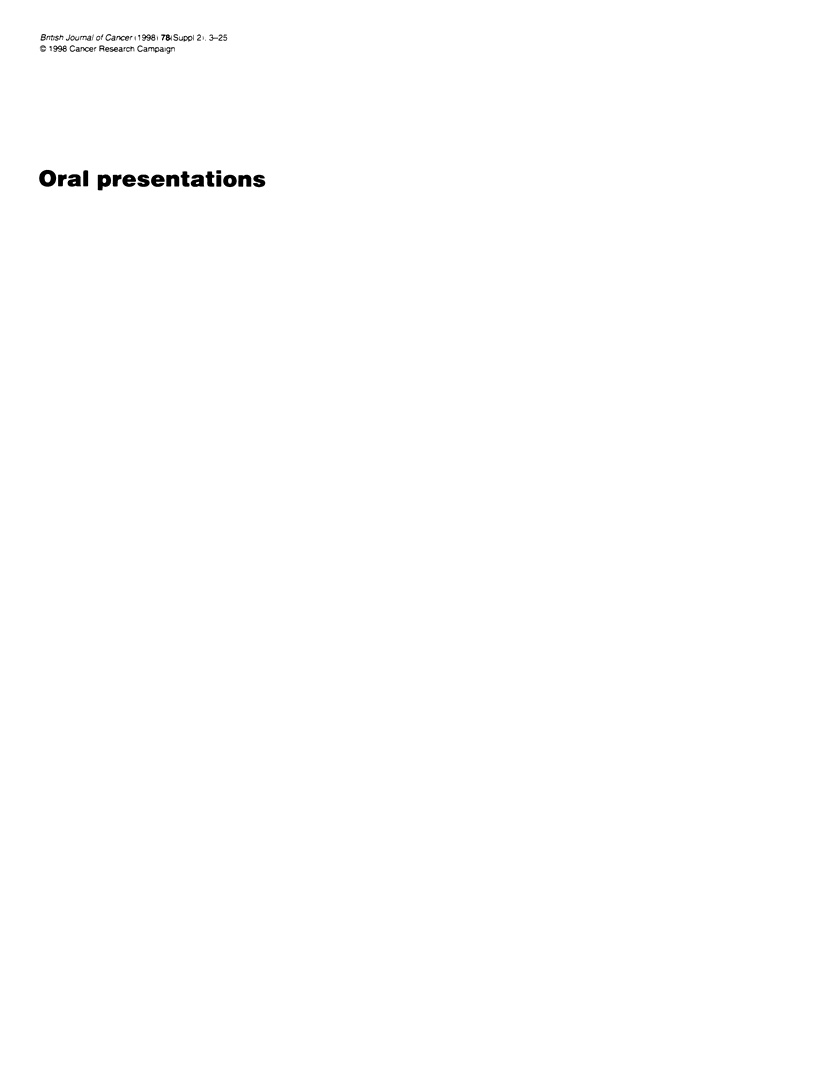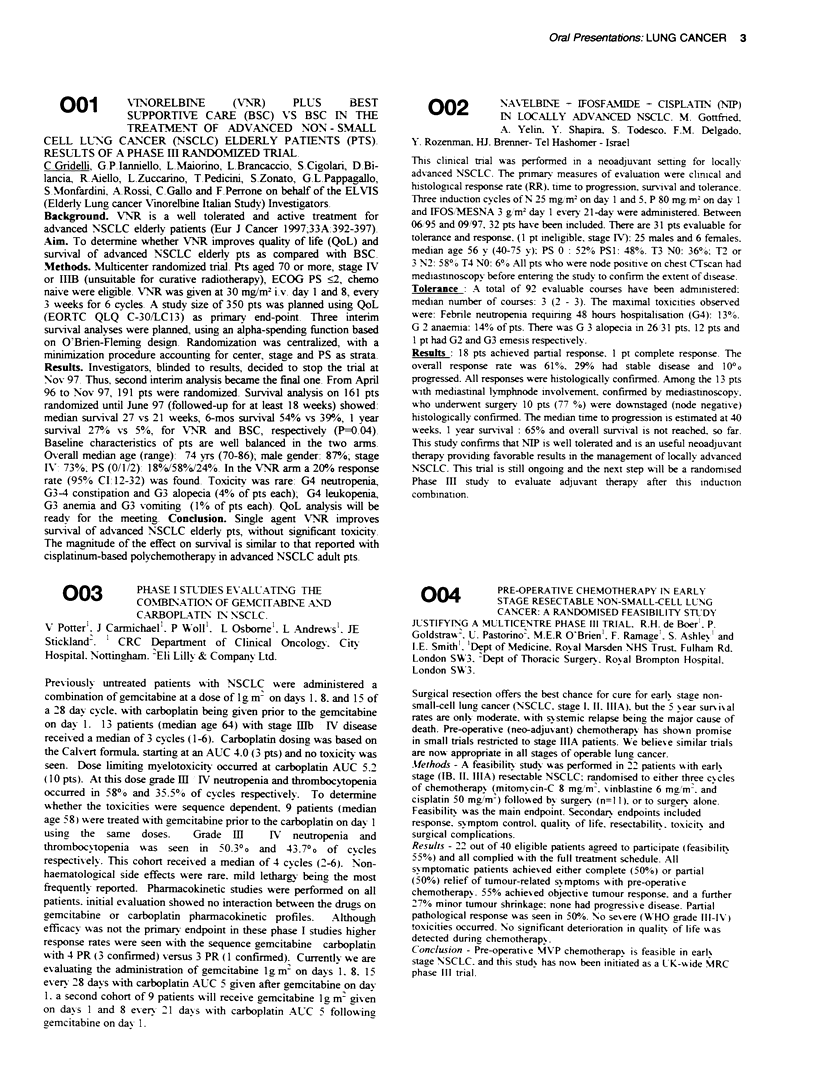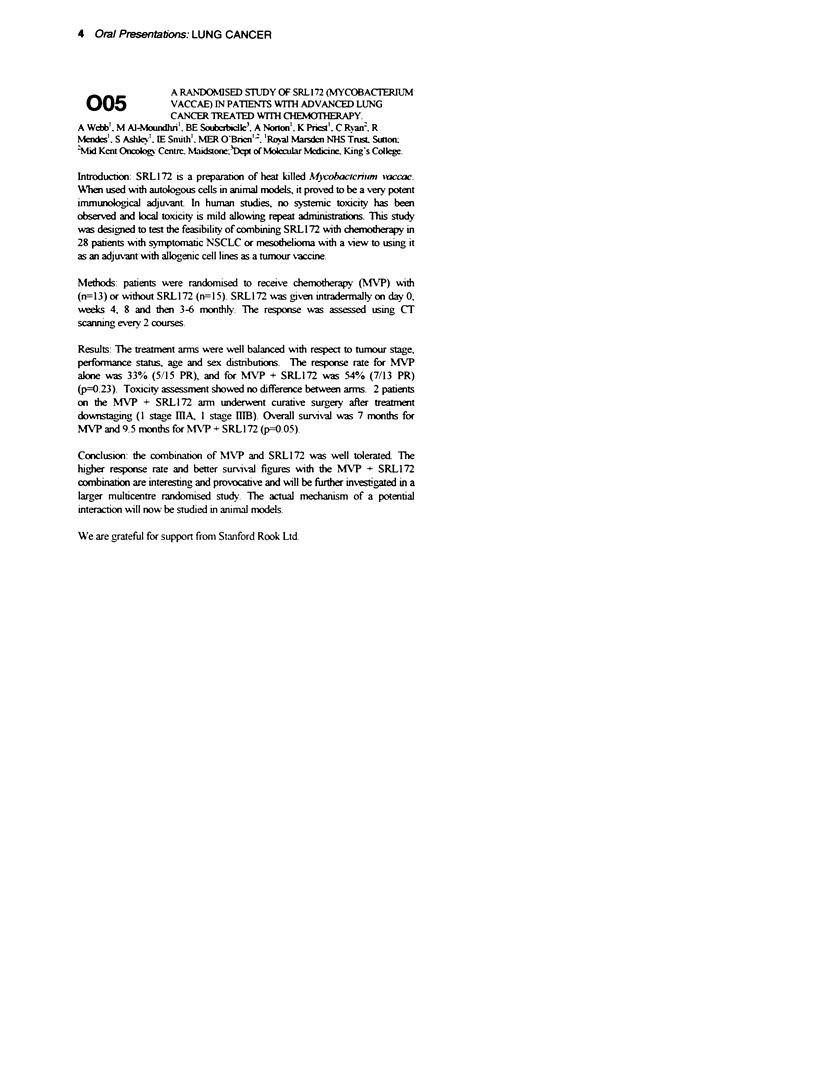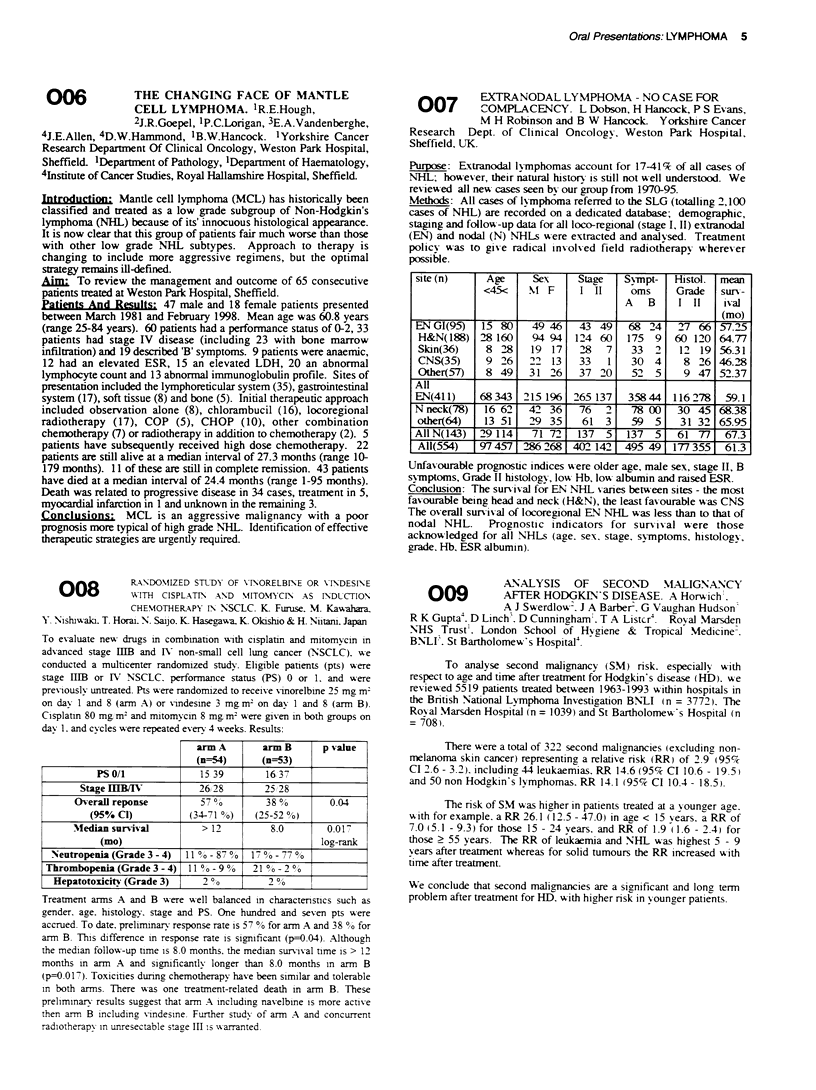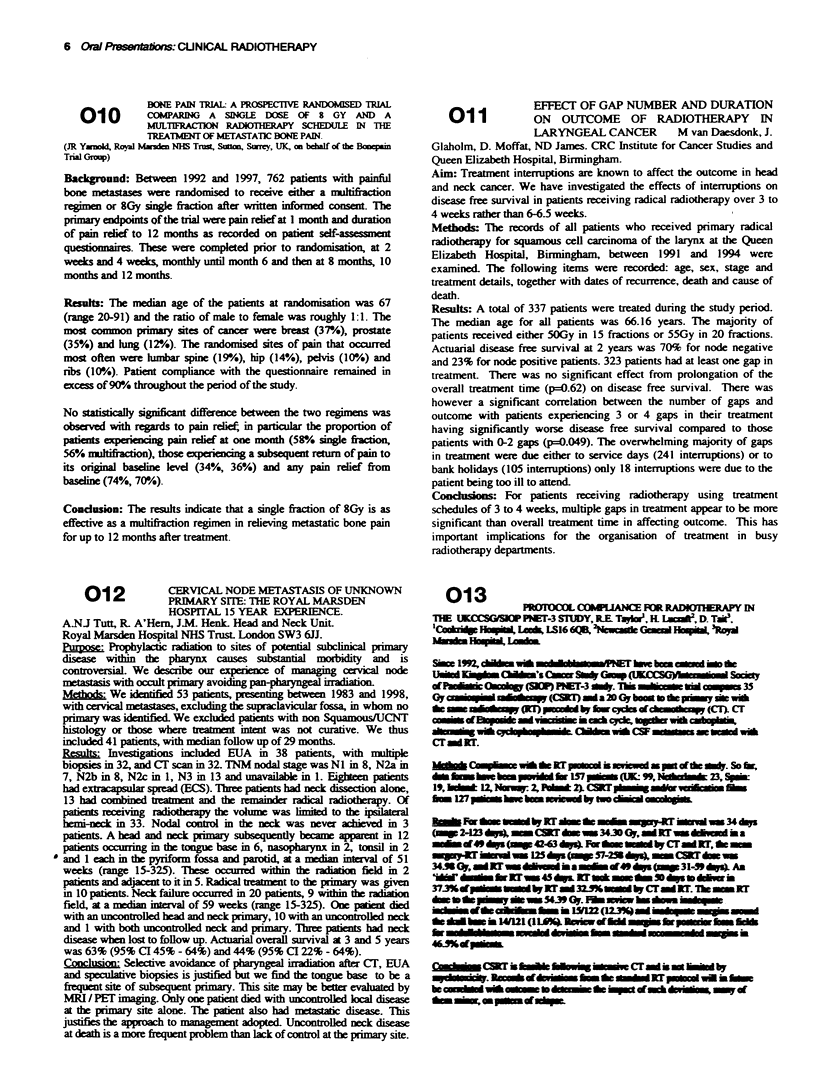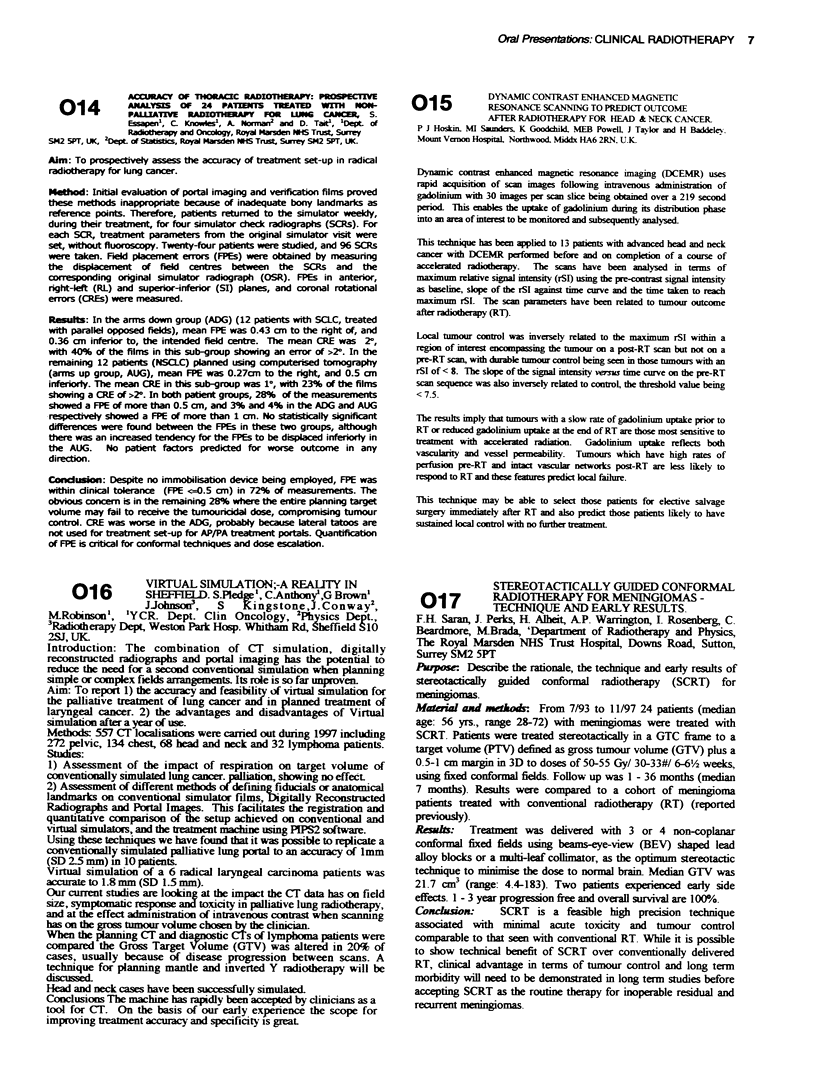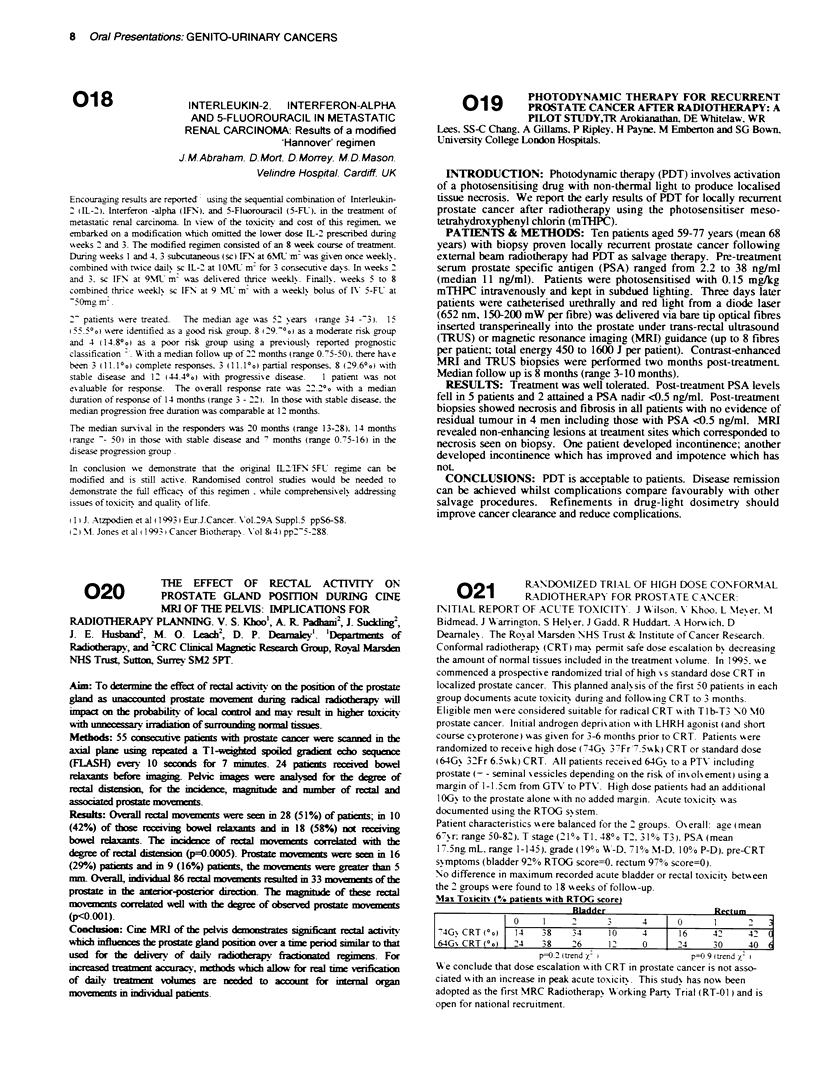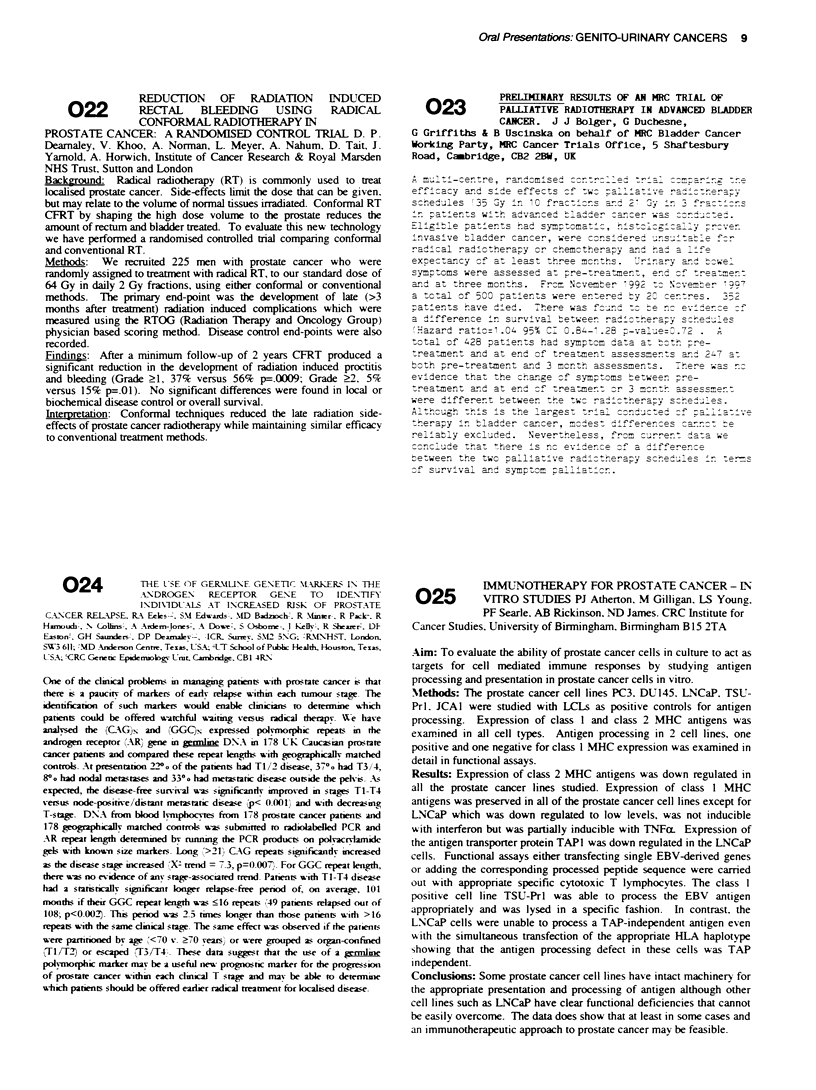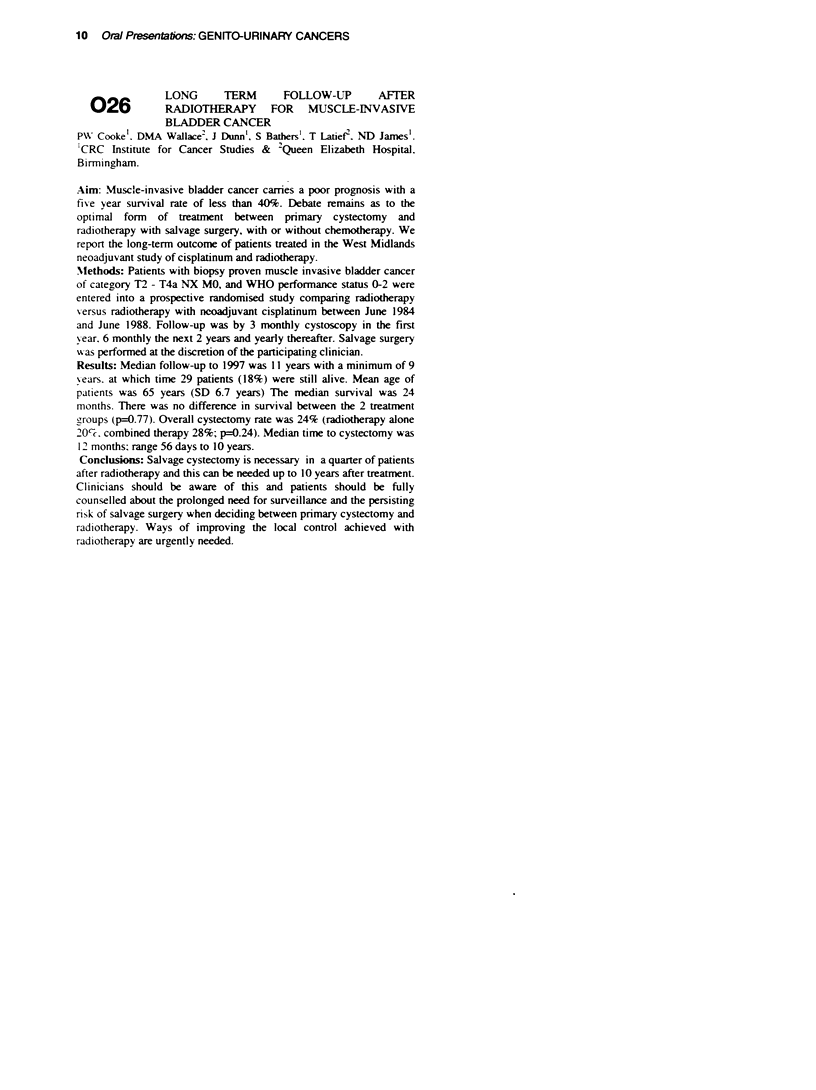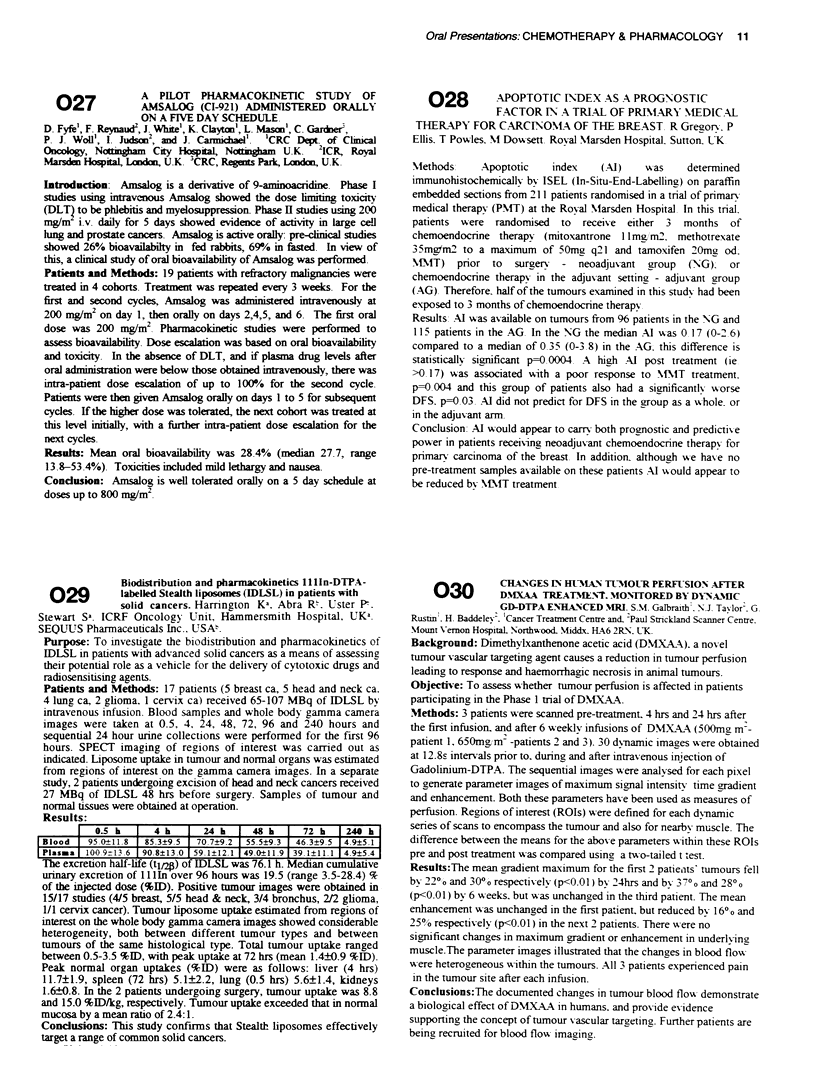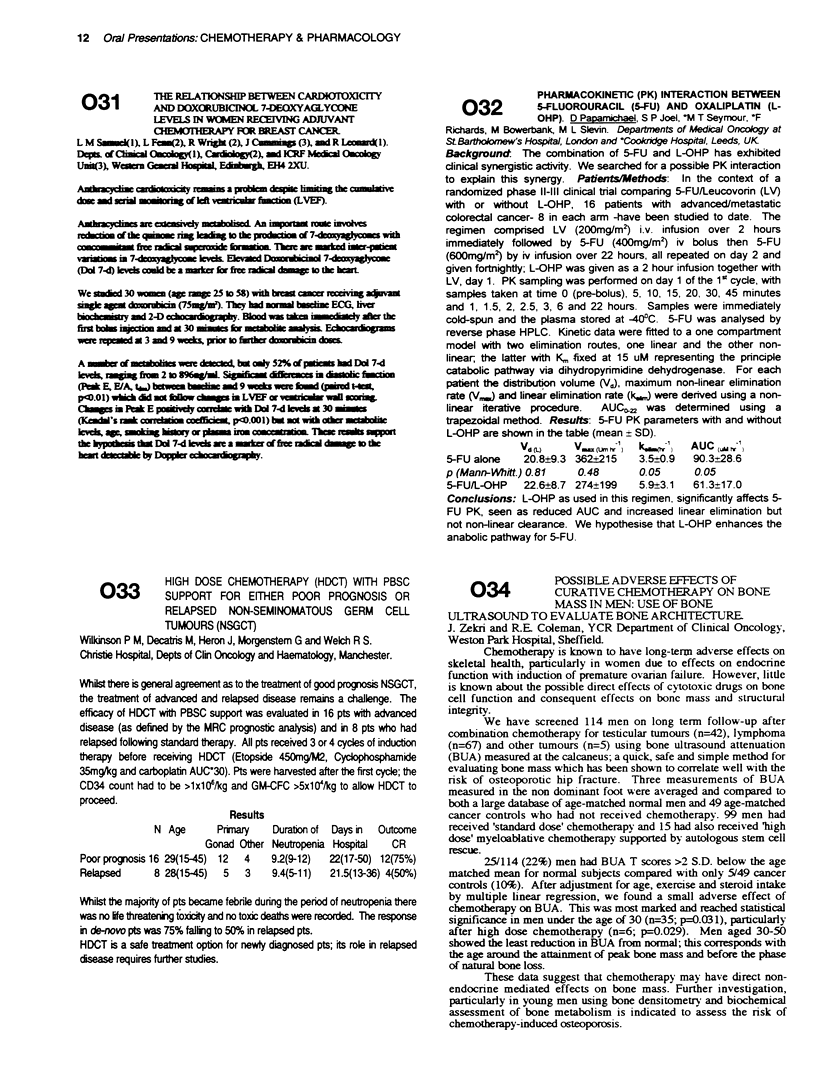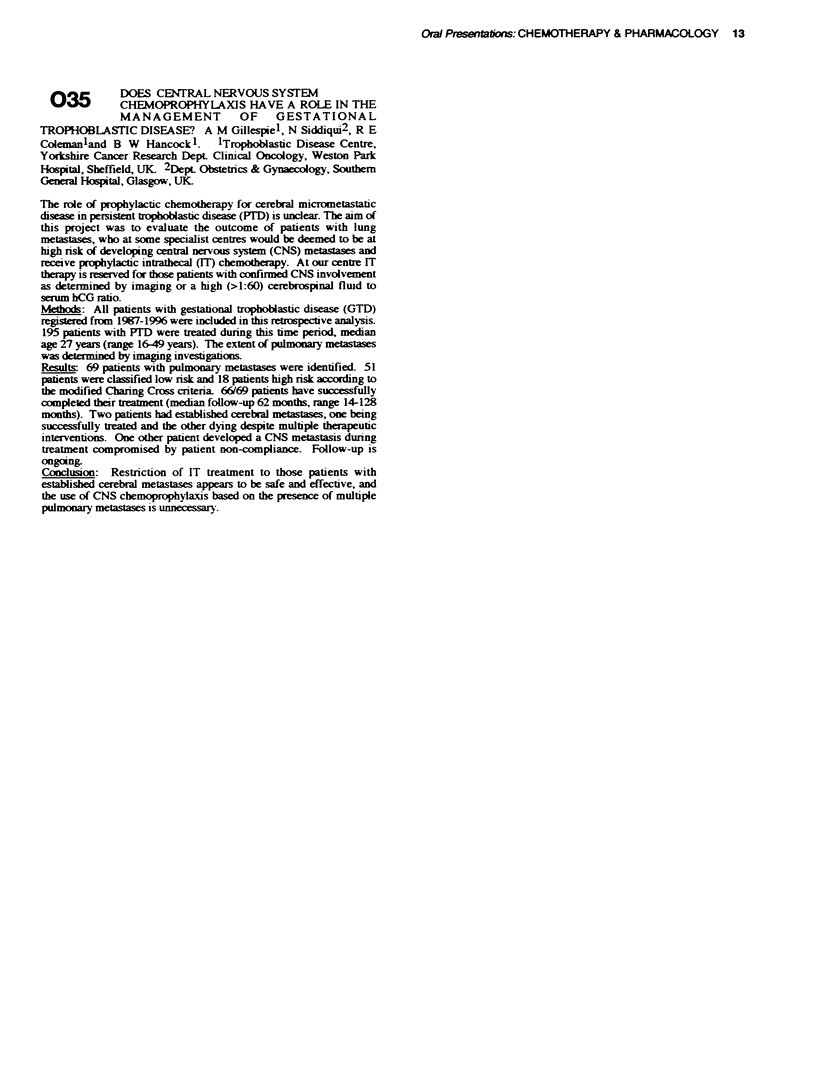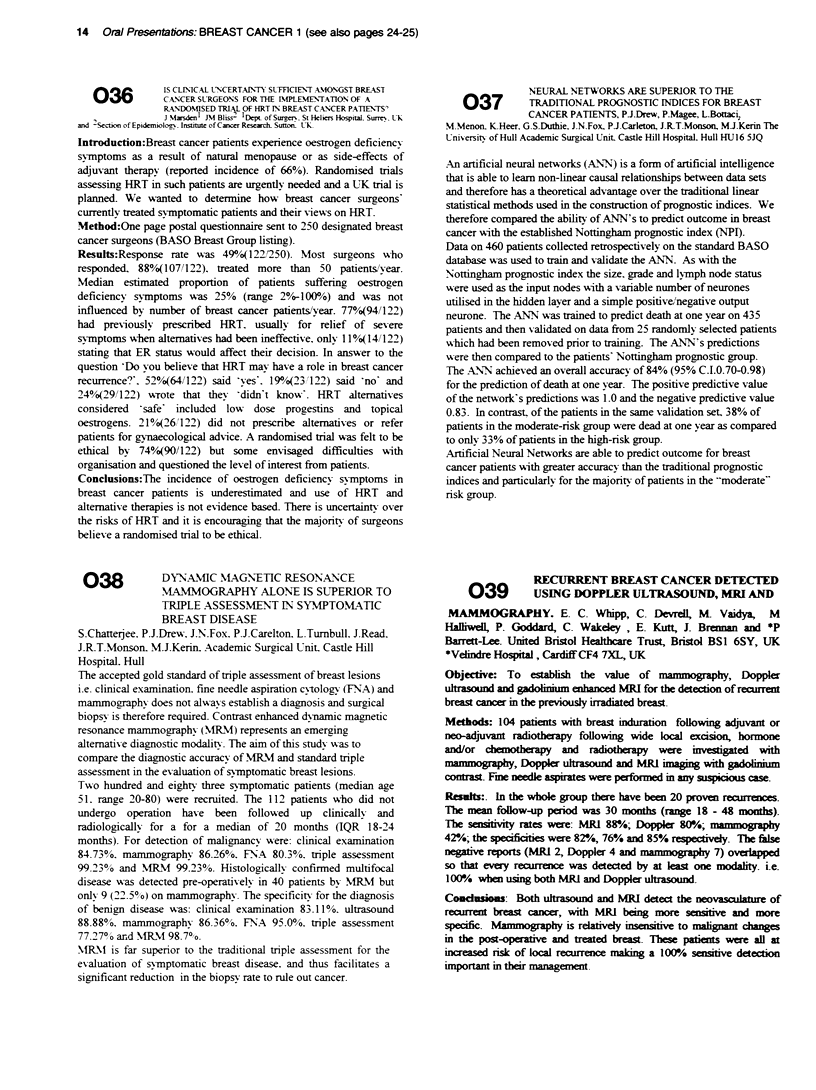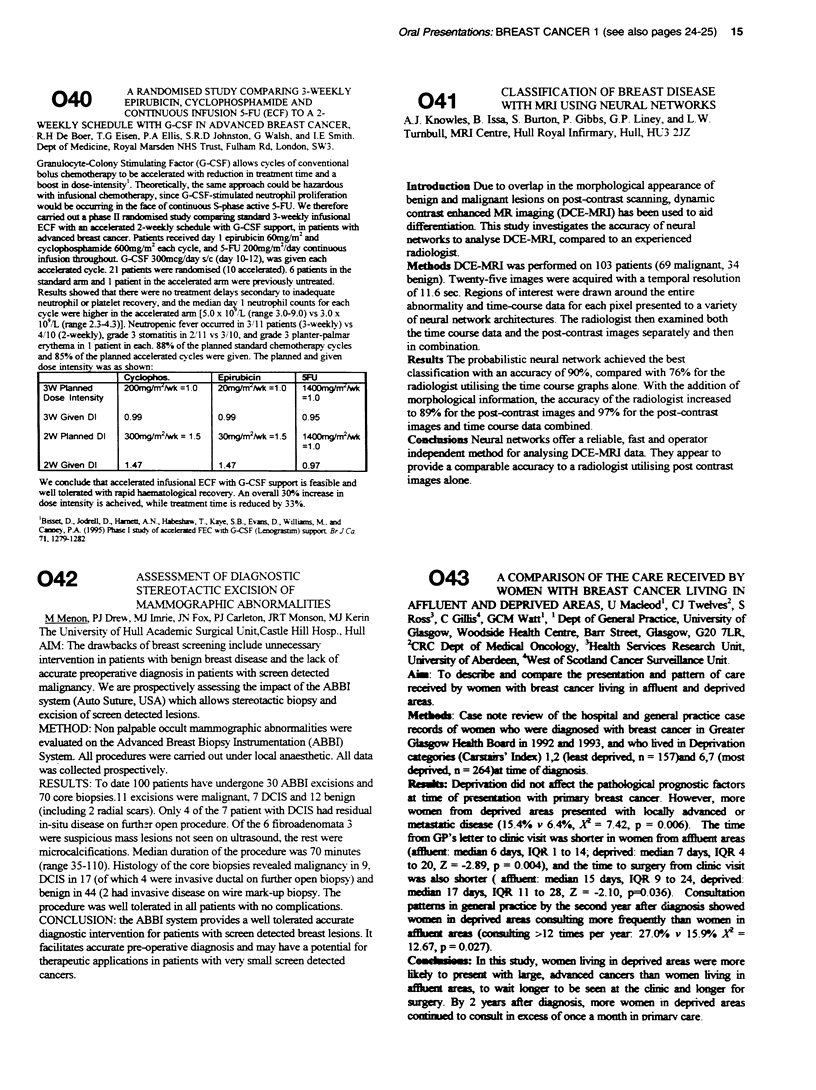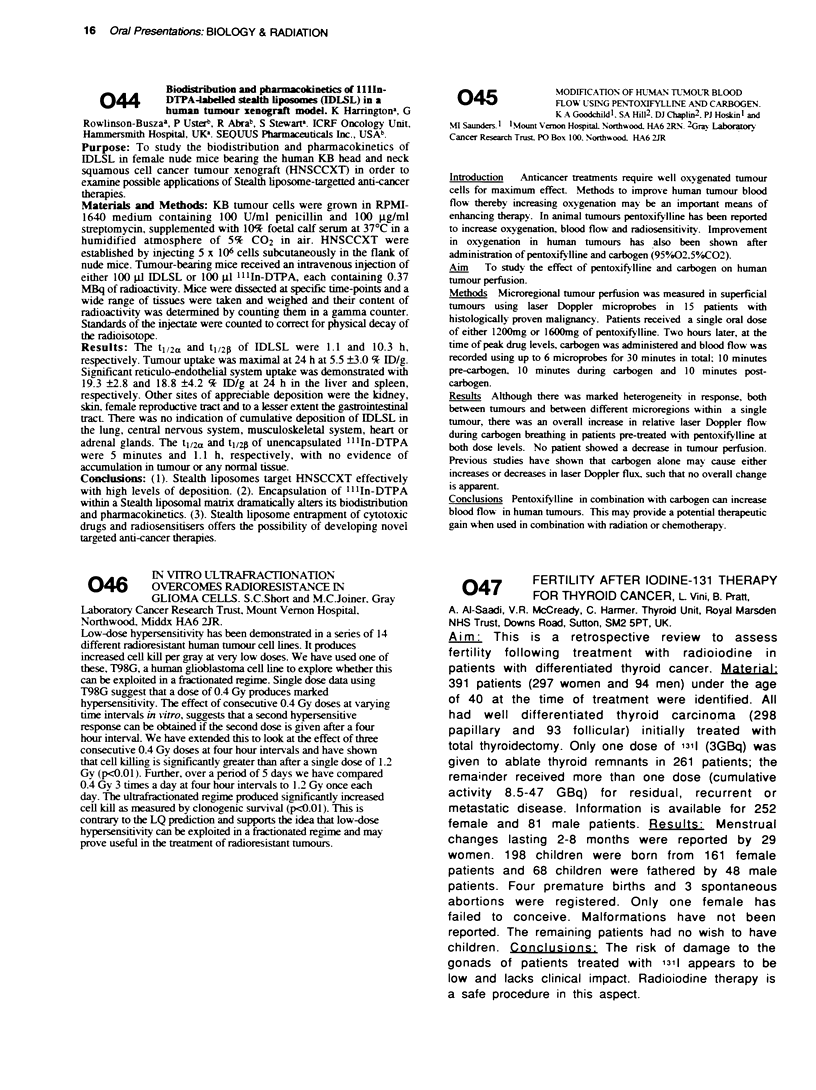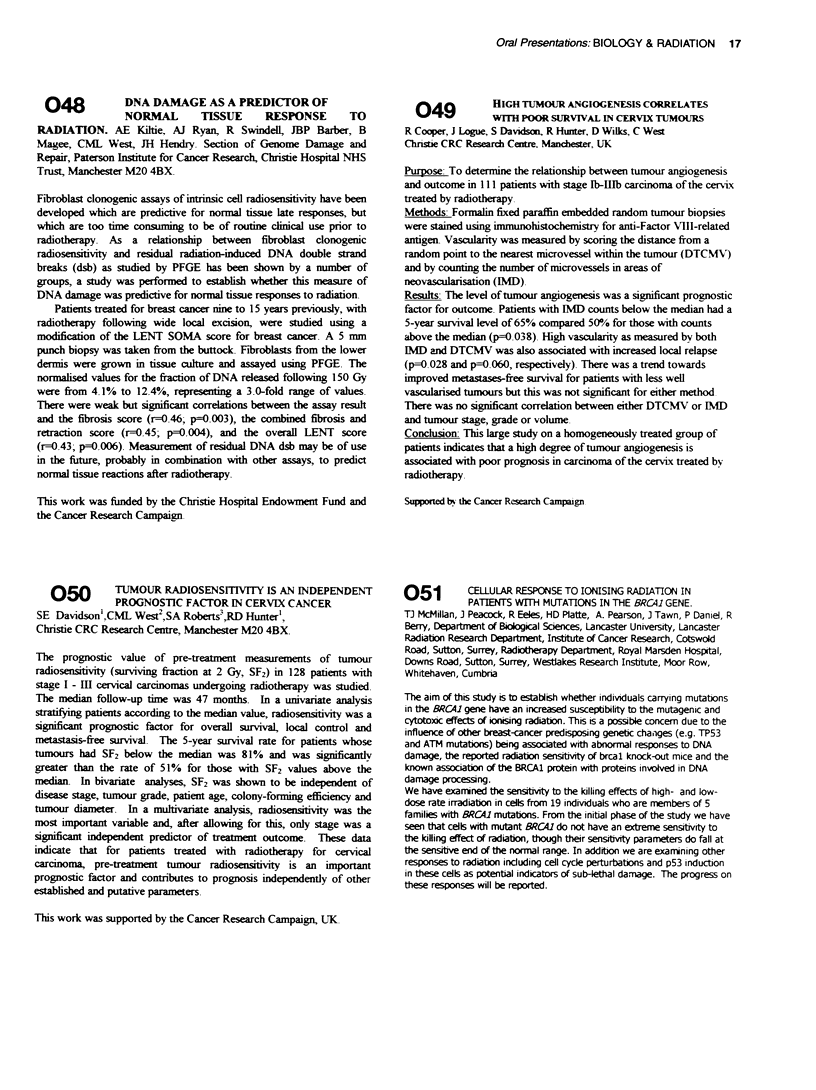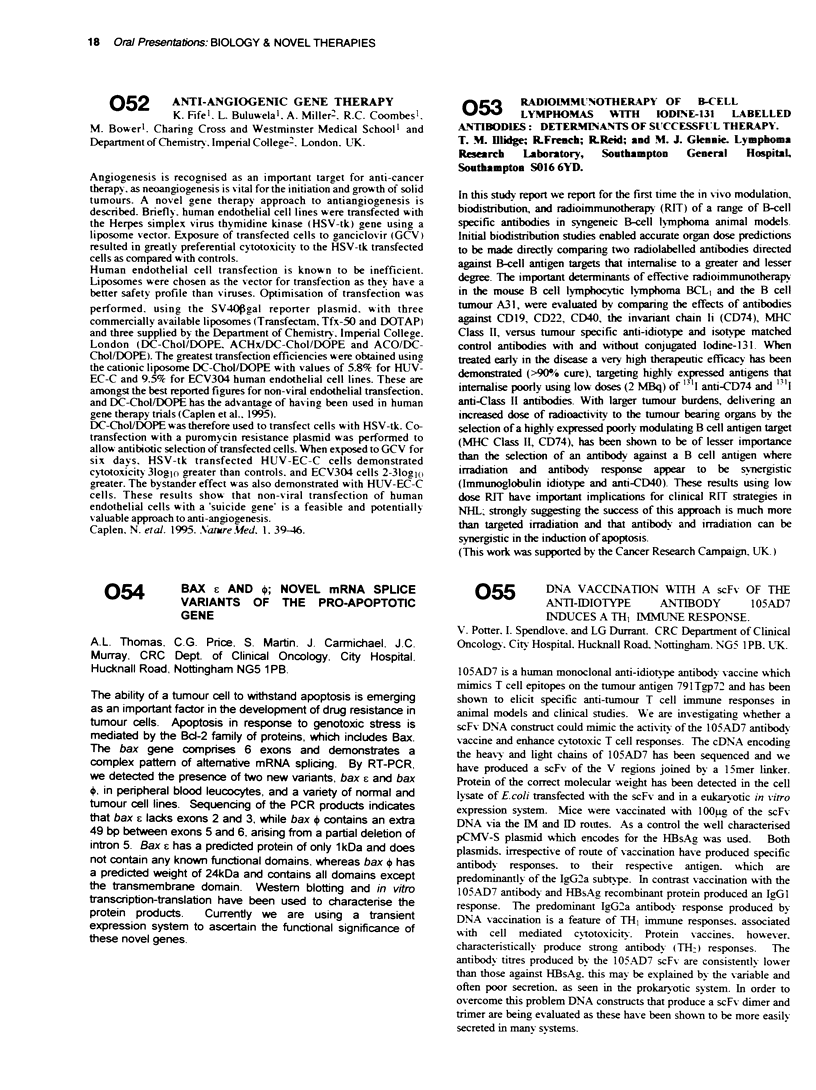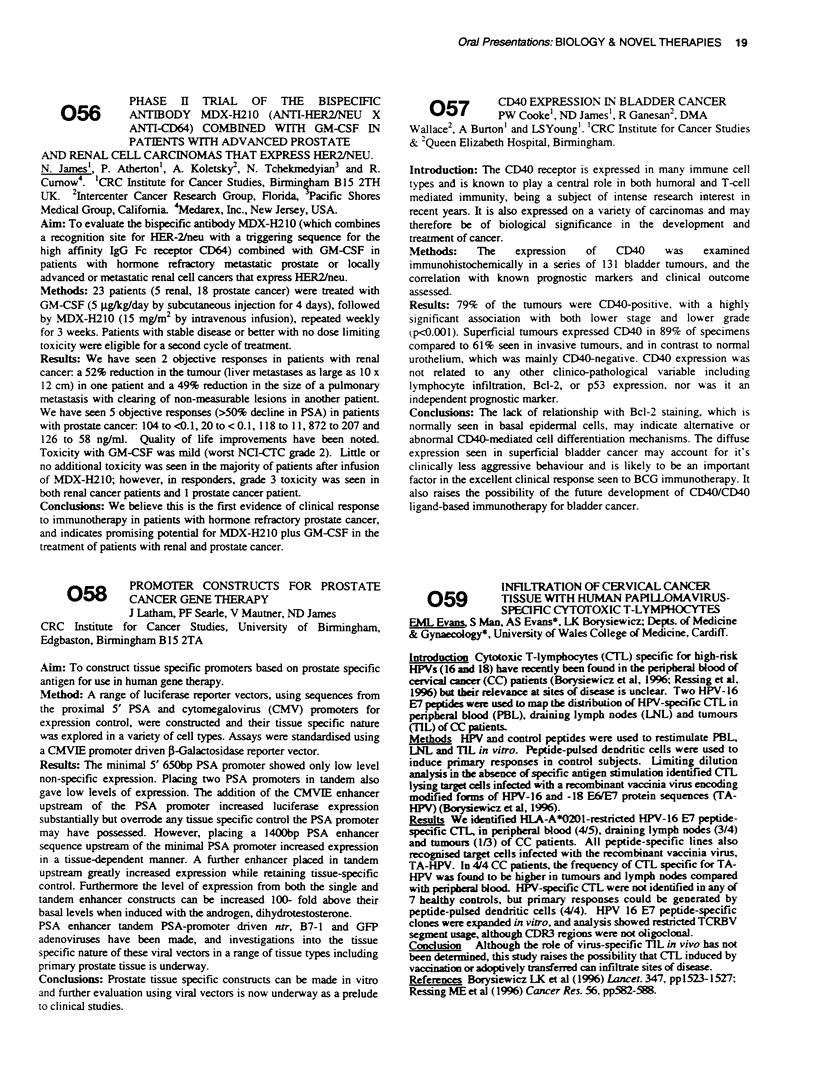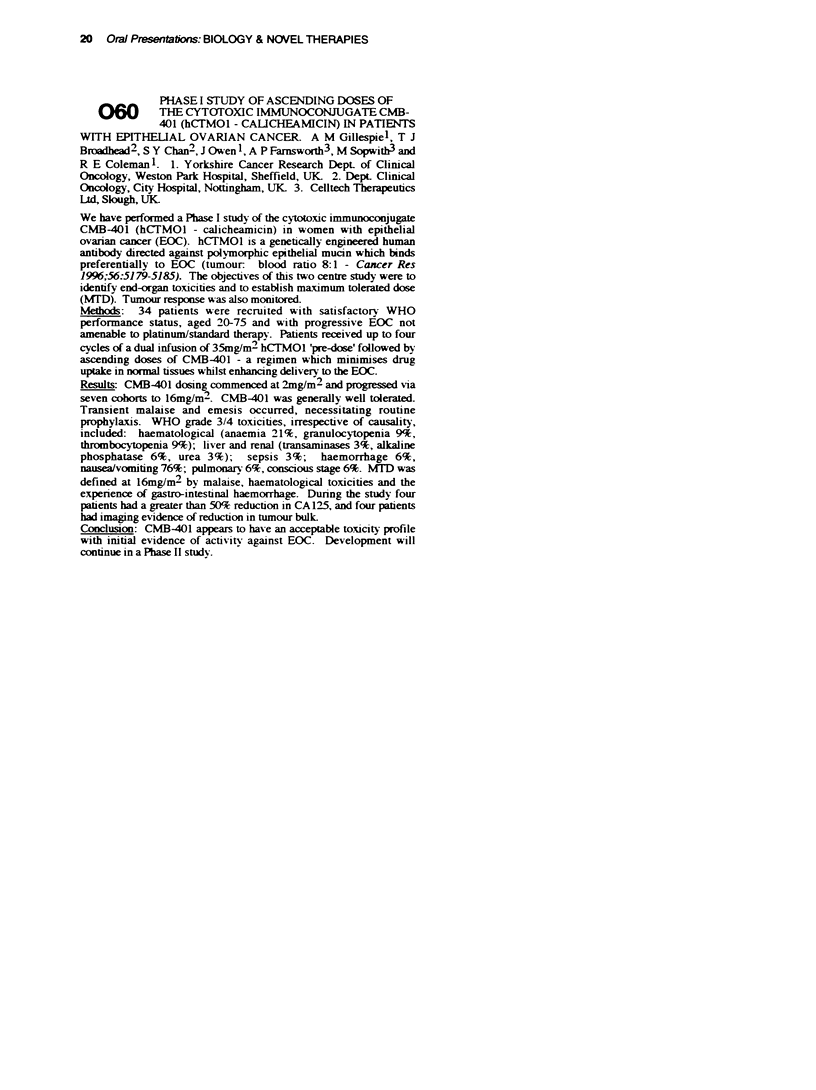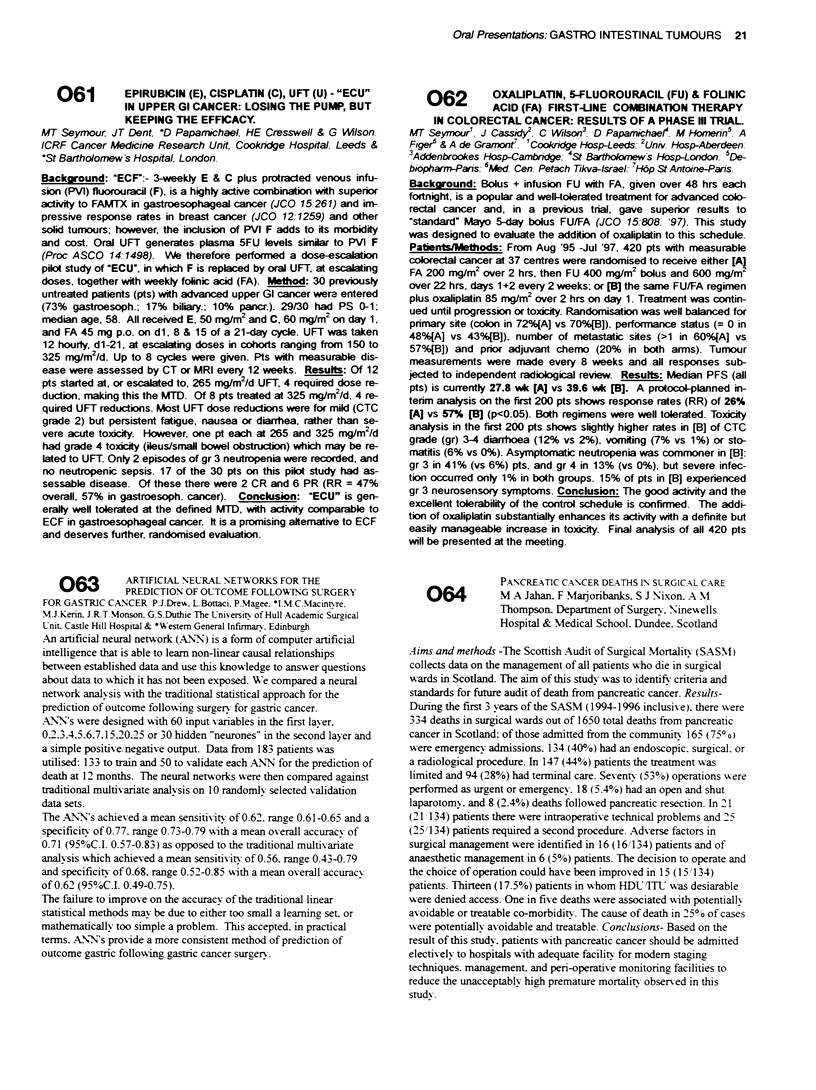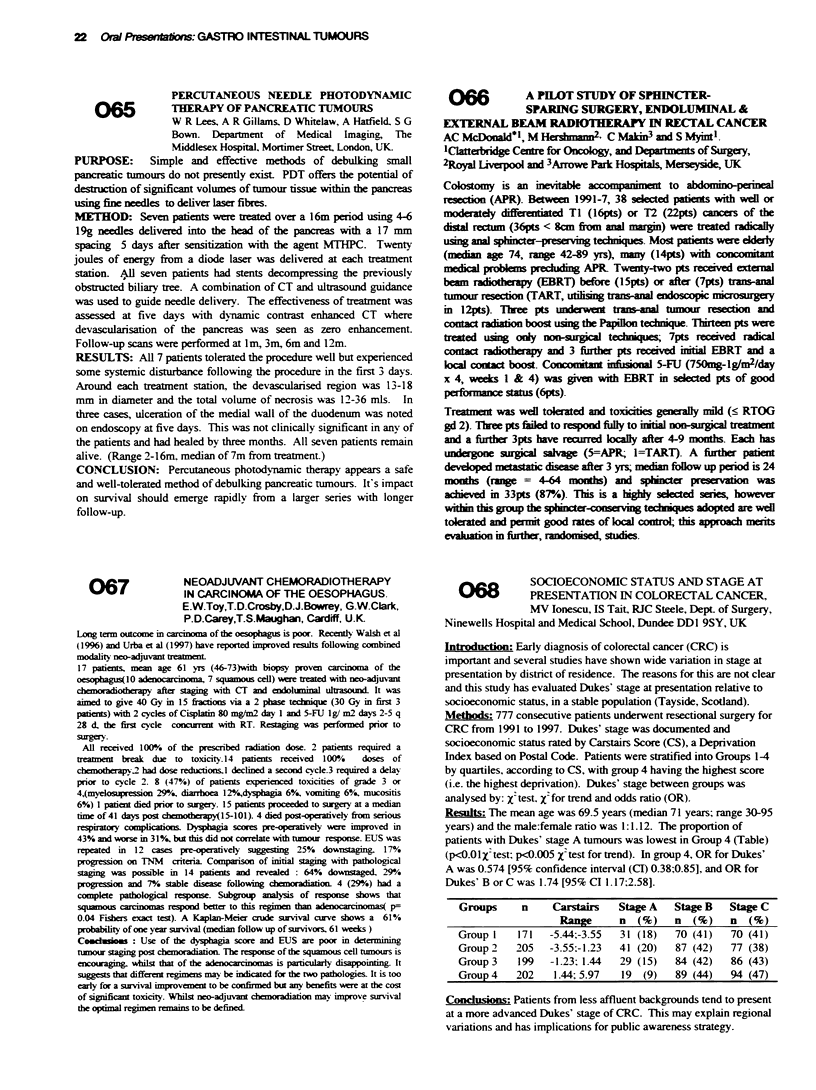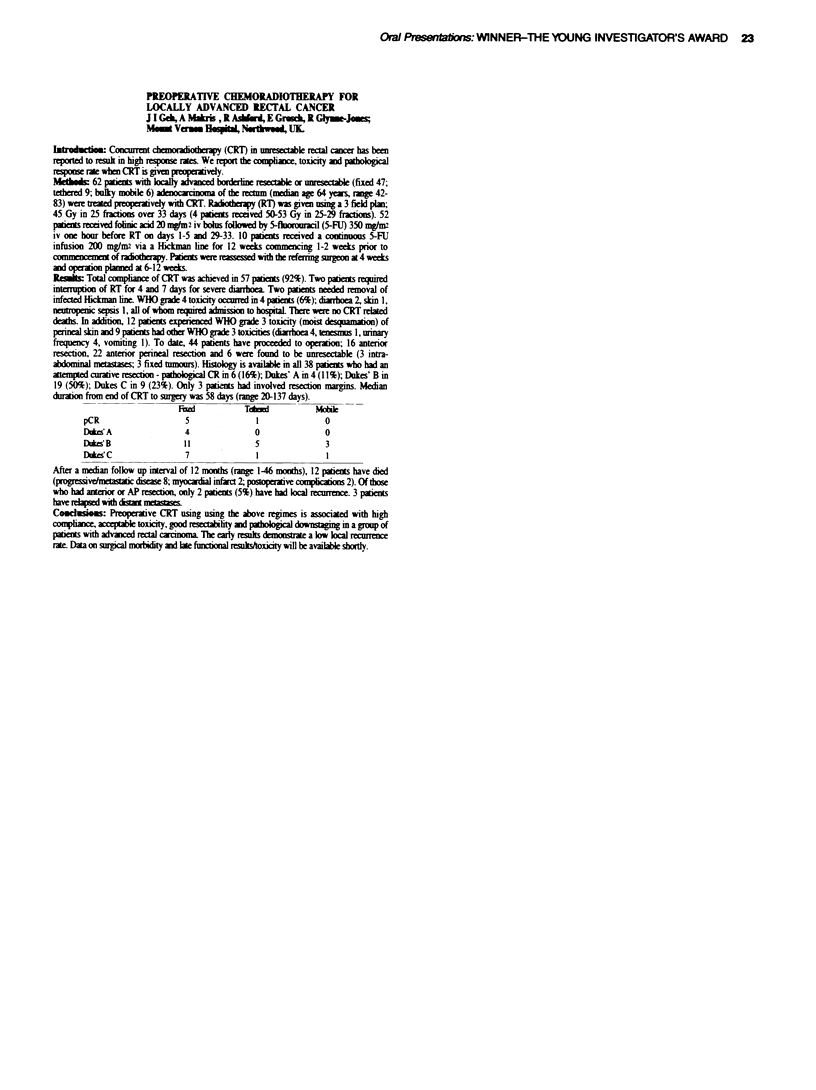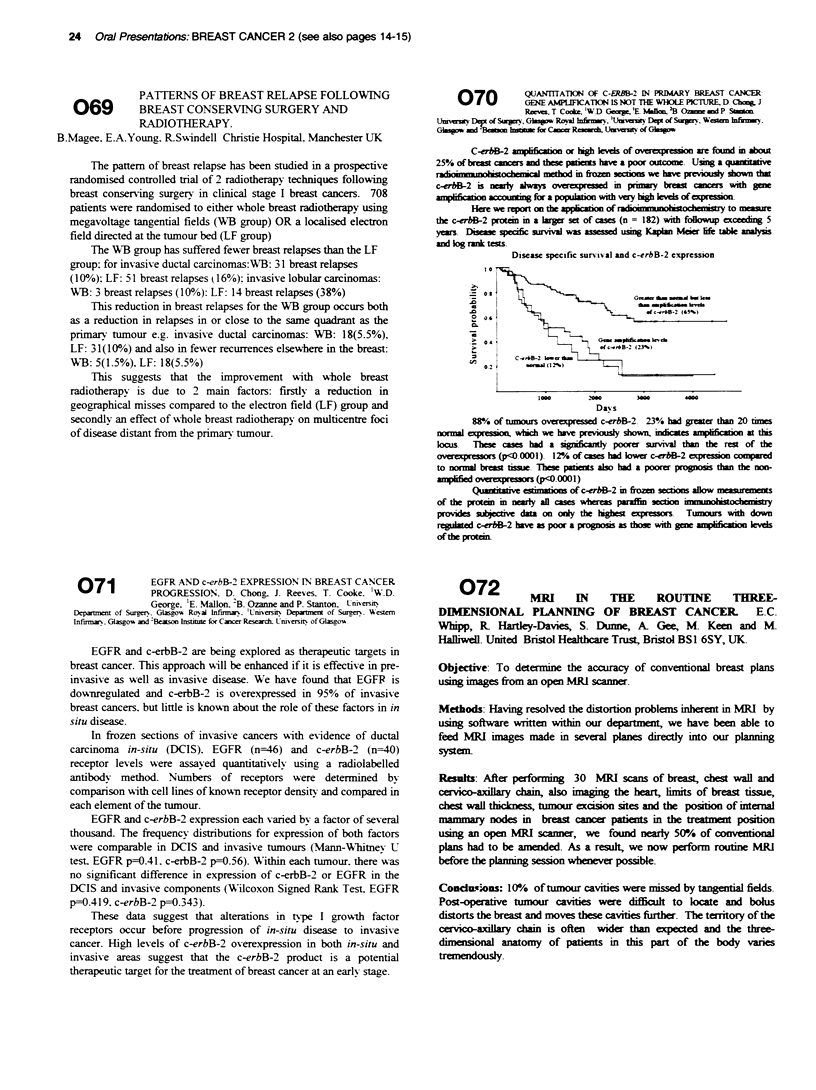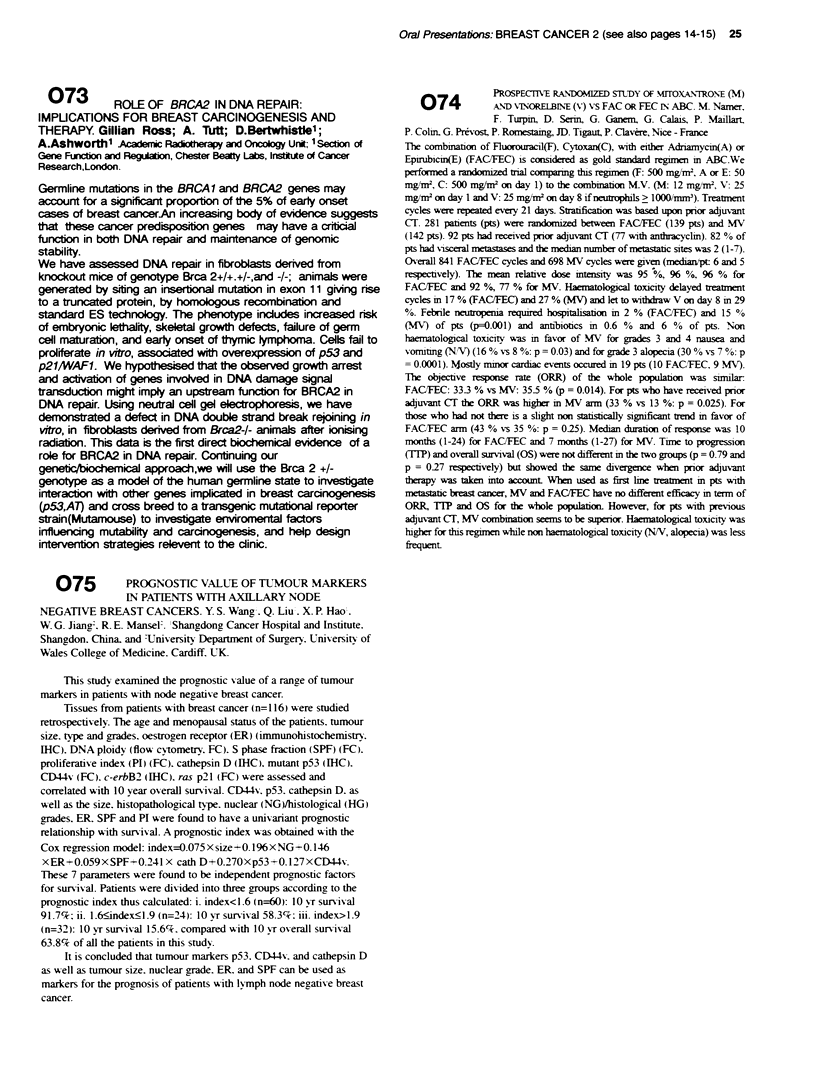# Joint meeting of the British Oncological Association, Association of Cancer Physicians, Royal College of Radiologists. Nottingham, United Kingdom. 5-7 July 1998. Abstracts.

**Published:** 1998

**Authors:** 


British Journal of Cancer (1998) 78(Suppl 2), 3–25

© 1998 Cancer Research Campaign

### Oral presentations

### O01 VINORELBINE (VNR) PLUS BEST SUPPORTIVE CARE (BSC) VS BSC IN THE TREATMENT OF ADVANCED NON-SMALL CELL LUNG CANCER (NSCLC) ELDERLY PATIENTS (PTS). RESULTS OF A PHASE III RANDOMIZED TRIAL.

C.Gridelli, G.P.Ianniello, L.Maiorino, L.Brancaccio, S.Cigolari, D.Bilancia, R.Aiello, L.Zuccarino, T.Pedicini, S.Zonato, G.L.Pappagallo, S.Monfardini, A.Rossi, C.Gallo and F.Perrone on behalf of the ELVIS (Elderly Lung cancer Vinorelbine Italian Study) Investigators.

**Background.** VNR is a well tolerated and active treatment for advanced NSCLC elderly patients (Eur J Cancer 1997;33A:392-397). **Aim.** To determine whether VNR improves quality of life (QoL) and survival of advanced NSCLC elderly pts as compared with BSC. **Methods.** Multicenter randomized trial. Pts aged 70 or more, stage IV or IIIB (unsuitable for curative radiotherapy), ECOG PS ≤2, chemo naive were eligible. VNR was given at 30 mg/m² i.v. day 1 and 8, every 3 weeks for 6 cycles. A study size of 350 pts was planned using QoL (EORTC QLQ C-30/LC13) as primary end-point. Three interim survival analyses were planned, using an alpha-spending function based on O'Brien-Fleming design. Randomization was centralized, with a minimization procedure accounting for center, stage and PS as strata. **Results.** Investigators, blinded to results, decided to stop the trial at Nov 97. Thus, second interim analysis became the final one. From April 96 to Nov 97, 191 pts were randomized. Survival analysis on 161 pts randomized until June 97 (followed-up for at least 18 weeks) showed: median survival 27 vs 21 weeks, 6-mos survival 54% vs 39%, 1 year survival 27% vs 5%, for VNR and BSC, respectively (P=0.04). Baseline characteristics of pts are well balanced in the two arms. Overall median age (range): 74 yrs (70-86); male gender: 87%; stage IV: 73%; PS (0/1/2): 18%/58%/24%. In the VNR arm a 20% response rate (95% CI:12-32) was found. Toxicity was rare: G4 neutropenia, G3-4 constipation and G3 alopecia (4% of pts each); G4 leukopenia, G3 anemia and G3 vomiting (1% of pts each). QoL analysis will be ready for the meeting. **Conclusion.** Single agent VNR improves survival of advanced NSCLC elderly pts, without significant toxicity. The magnitude of the effect on survival is similar to that reported with cisplatinum-based polychemotherapy in advanced NSCLC adult pts.

### O02 NAVELBINE - IFOSFAMIDE - CISPLATIN (NIP) IN LOCALLY ADVANCED NSCLC.

M. Gottfried, A. Yelin, Y. Shapira, S. Todesco, F.M. Delgado, Y. Rozenman, HJ. Brenner- Tel Hashomer - Israel

This clinical trial was performed in a neoadjuvant setting for locally advanced NSCLC. The primary measures of evaluation were clinical and histological response rate (RR), time to progression, survival and tolerance. Three induction cycles of N 25 mg/m² on day 1 and 5, P 80 mg/m² on day 1 and IFOS/MESNA 3 g/m² day 1 every 21-day were administered. Between 06/95 and 09/97, 32 pts have been included. There are 31 pts evaluable for tolerance and response, (1 pt ineligible, stage IV): 25 males and 6 females, median age 56 y (40-75 y); PS 0 : 52% PS1: 48%. T3 N0: 36%; T2 or 3 N2: 58% T4 N0: 6% All pts who were node positive on chest CTscan had mediastinoscopy before entering the study to confirm the extent of disease.

**Tolerance** : A total of 92 evaluable courses have been administered: median number of courses: 3 (2 - 3). The maximal toxicities observed were: Febrile neutropenia requiring 48 hours hospitalisation (G4): 13%. G 2 anaemia: 14% of pts. There was G 3 alopecia in 26/31 pts. 12 pts and 1 pt had G2 and G3 emesis respectively.

**Results** : 18 pts achieved partial response, 1 pt complete response. The overall response rate was 61%, 29% had stable disease and 10% progressed. All responses were histologically confirmed. Among the 13 pts with mediastinal lymphnode involvement, confirmed by mediastinoscopy, who underwent surgery 10 pts (77 %) were downstaged (node negative) histologically confirmed. The median time to progression is estimated at 40 weeks, 1 year survival : 65% and overall survival is not reached, so far. This study confirms that NIP is well tolerated and is an useful neoadjuvant therapy providing favorable results in the management of locally advanced NSCLC. This trial is still ongoing and the next step will be a randomised Phase III study to evaluate adjuvant therapy after this induction combination.

### O03 PHASE I STUDIES EVALUATING THE COMBINATION OF GEMCITABINE AND CARBOPLATIN IN NSCLC.

V Potter[1], J Carmichael[1], P Woll[1], L Osborne[1], L Andrews[1], JE Stickland[2]. [1] CRC Department of Clinical Oncology, City Hospital, Nottingham. [2]Eli Lilly & Company Ltd.

Previously untreated patients with NSCLC were administered a combination of gemcitabine at a dose of 1g/m² on days 1, 8, and 15 of a 28 day cycle, with carboplatin being given prior to the gemcitabine on day 1. 13 patients (median age 64) with stage IIIb / IV disease received a median of 3 cycles (1-6). Carboplatin dosing was based on the Calvert formula, starting at an AUC 4.0 (3 pts) and no toxicity was seen. Dose limiting myelotoxicity occurred at carboplatin AUC 5.2 (10 pts). At this dose grade III / IV neutropenia and thrombocytopenia occurred in 58% and 35.5% of cycles respectively. To determine whether the toxicities were sequence dependent, 9 patients (median age 58) were treated with gemcitabine prior to the carboplatin on day 1 using the same doses. Grade III / IV neutropenia and thrombocytopenia was seen in 50.3% and 43.7% of cycles respectively. This cohort received a median of 4 cycles (2-6). Non-haematological side effects were rare, mild lethargy being the most frequently reported. Pharmacokinetic studies were performed on all patients, initial evaluation showed no interaction between the drugs on gemcitabine or carboplatin pharmacokinetic profiles. Although efficacy was not the primary endpoint in these phase I studies higher response rates were seen with the sequence gemcitabine carboplatin with 4 PR (3 confirmed) versus 3 PR (1 confirmed). Currently we are evaluating the administration of gemcitabine 1g/m² on days 1, 8, 15 every 28 days with carboplatin AUC 5 given after gemcitabine on day 1, a second cohort of 9 patients will receive gemcitabine 1g/m² given on days 1 and 8 every 21 days with carboplatin AUC 5 following gemcitabine on day 1.

### O04 PRE-OPERATIVE CHEMOTHERAPY IN EARLY STAGE RESECTABLE NON-SMALL-CELL LUNG CANCER: A RANDOMISED FEASIBILITY STUDY JUSTIFYING A MULTICENTRE PHASE III TRIAL.

R.H. de Boer[1], P. Goldstraw[2], U. Pastorino[2], M.E.R O'Brien[1], F. Ramage[1], S. Ashley[1] and I.E. Smith[1]. [1]Dept of Medicine, Royal Marsden NHS Trust, Fulham Rd, London SW3. [2]Dept of Thoracic Surgery, Royal Brompton Hospital, London SW3.

Surgical resection offers the best chance for cure for early stage non-small-cell lung cancer (NSCLC, stage I, II, IIIA), but the 5 year survival rates are only moderate, with systemic relapse being the major cause of death. Pre-operative (neo-adjuvant) chemotherapy has shown promise in small trials restricted to stage IIIA patients. We believe similar trials are now appropriate in all stages of operable lung cancer.

*Methods* - A feasibility study was performed in 22 patients with early stage (IB, II, IIIA) resectable NSCLC: randomised to either three cycles of chemotherapy (mitomycin-C 8 mg/m², vinblastine 6 mg/m², and cisplatin 50 mg/m²) followed by surgery (n=11), or to surgery alone. Feasibility was the main endpoint. Secondary endpoints included response, symptom control, quality of life, resectability, toxicity and surgical complications.

*Results* - 22 out of 40 eligible patients agreed to participate (feasibility 55%) and all complied with the full treatment schedule. All symptomatic patients achieved either complete (50%) or partial (50%) relief of tumour-related symptoms with pre-operative chemotherapy. 55% achieved objective tumour response, and a further 27% minor tumour shrinkage; none had progressive disease. Partial pathological response was seen in 50%. No severe (WHO grade III-IV) toxicities occurred. No significant deterioration in quality of life was detected during chemotherapy.

*Conclusion* - Pre-operative MVP chemotherapy is feasible in early stage NSCLC, and this study has now been initiated as a UK-wide MRC phase III trial.

### O05 A RANDOMISED STUDY OF SRL172 (MYCOBACTERIUM VACCAE) IN PATIENTS WITH ADVANCED LUNG CANCER TREATED WITH CHEMOTHERAPY.

A Webb[1], M Al-Moundhri[1], BE Souberbielle[3], A Norton[1], K Priest[1], C Ryan[2], R Mendes[1], S Ashley[1], IE Smith[1], MER O'Brien[1,2], [1]Royal Marsden NHS Trust, Sutton; [2]Mid Kent Oncology Centre, Maidstone; [3]Dept of Molecular Medicine, King's College.

Introduction: SRL172 is a preparation of heat killed *Mycobacterium vaccae*. When used with autologous cells in animal models, it proved to be a very potent immunological adjuvant. In human studies, no systemic toxicity has been observed and local toxicity is mild allowing repeat administrations. This study was designed to test the feasibility of combining SRL172 with chemotherapy in 28 patients with symptomatic NSCLC or mesothelioma with a view to using it as an adjuvant with allogenic cell lines as a tumour vaccine.

Methods: patients were randomised to receive chemotherapy (MVP) with (n=13) or without SRL172 (n=15). SRL172 was given intradermally on day 0, weeks 4, 8 and then 3-6 monthly. The response was assessed using CT scanning every 2 courses.

Results: The treatment arms were well balanced with respect to tumour stage, performance status, age and sex distributions. The response rate for MVP alone was 33% (5/15 PR), and for MVP + SRL172 was 54% (7/13 PR) ($p=0.23$). Toxicity assessment showed no difference between arms. 2 patients on the MVP + SRL172 arm underwent curative surgery after treatment downstaging (1 stage IIIA, 1 stage IIIB). Overall survival was 7 months for MVP and 9.5 months for MVP + SRL172 ($p=0.05$).

Conclusion: the combination of MVP and SRL172 was well tolerated. The higher response rate and better survival figures with the MVP + SRL172 combination are interesting and provocative and will be further investigated in a larger multicentre randomised study. The actual mechanism of a potential interaction will now be studied in animal models.

We are grateful for support from Stanford Rook Ltd.

### O06

**THE CHANGING FACE OF MANTLE CELL LYMPHOMA.** [1]R.E.Hough, [2]J.R.Goepel, [1]P.C.Lorigan, [3]E.A.Vandenberghe, [4]J.E.Allen, [4]D.W.Hammond, [1]B.W.Hancock. [1]Yorkshire Cancer Research Department Of Clinical Oncology, Weston Park Hospital, Sheffield. [1]Department of Pathology, [1]Department of Haematology, [4]Institute of Cancer Studies, Royal Hallamshire Hospital, Sheffield.

**Introduction:** Mantle cell lymphoma (MCL) has historically been classified and treated as a low grade subgroup of Non-Hodgkin's lymphoma (NHL) because of its' innocuous histological appearance. It is now clear that this group of patients fair much worse than those with other low grade NHL subtypes. Approach to therapy is changing to include more aggressive regimens, but the optimal strategy remains ill-defined.
**Aim:** To review the management and outcome of 65 consecutive patients treated at Weston Park Hospital, Sheffield.
**Patients And Results:** 47 male and 18 female patients presented between March 1981 and February 1998. Mean age was 60.8 years (range 25-84 years). 60 patients had a performance status of 0-2, 33 patients had stage IV disease (including 23 with bone marrow infiltration) and 19 described 'B' symptoms. 9 patients were anaemic, 12 had an elevated ESR, 15 an elevated LDH, 20 an abnormal lymphocyte count and 13 abnormal immunoglobulin profile. Sites of presentation included the lymphoreticular system (35), gastrointestinal system (17), soft tissue (8) and bone (5). Initial therapeutic approach included observation alone (8), chlorambucil (16), locoregional radiotherapy (17), COP (5), CHOP (10), other combination chemotherapy (7) or radiotherapy in addition to chemotherapy (2). 5 patients have subsequently received high dose chemotherapy. 22 patients are still alive at a median interval of 27.3 months (range 10-179 months). 11 of these are still in complete remission. 43 patients have died at a median interval of 24.4 months (range 1-95 months). Death was related to progressive disease in 34 cases, treatment in 5, myocardial infarction in 1 and unknown in the remaining 3.
**Conclusions:** MCL is an aggressive malignancy with a poor prognosis more typical of high grade NHL. Identification of effective therapeutic strategies are urgently required.

### O07

EXTRANODAL LYMPHOMA - NO CASE FOR COMPLACENCY. L Dobson, H Hancock, P S Evans, M H Robinson and B W Hancock. Yorkshire Cancer Research Dept. of Clinical Oncology, Weston Park Hospital, Sheffield, UK.

Purpose: Extranodal lymphomas account for 17-41% of all cases of NHL; however, their natural history is still not well understood. We reviewed all new cases seen by our group from 1970-95.
Methods: All cases of lymphoma referred to the SLG (totalling 2,100 cases of NHL) are recorded on a dedicated database; demographic, staging and follow-up data for all loco-regional (stage I, II) extranodal (EN) and nodal (N) NHLs were extracted and analysed. Treatment policy was to give radical involved field radiotherapy wherever possible.

| site (n) | Age <45< | Sex M F | Stage I II | Symptoms A B | Histol. Grade I II | mean survival (mo) |
|---|---|---|---|---|---|---|
| EN GI(95) | 15 80 | 49 46 | 43 49 | 68 24 | 27 66 | 57.25 |
| H&N(188) | 28 160 | 94 94 | 124 60 | 175 9 | 60 120 | 64.77 |
| Skin(36) | 8 28 | 19 17 | 28 7 | 33 2 | 12 19 | 56.31 |
| CNS(35) | 9 26 | 22 13 | 33 1 | 30 4 | 8 26 | 46.28 |
| Other(57) | 8 49 | 31 26 | 37 20 | 52 5 | 9 47 | 52.37 |
| All EN(411) | 68 343 | 215 196 | 265 137 | 358 44 | 116 278 | 59.1 |
| N neck(78) | 16 62 | 42 36 | 76 2 | 78 00 | 30 45 | 68.38 |
| other(64) | 13 51 | 29 35 | 61 3 | 59 5 | 31 32 | 65.95 |
| All N(143) | 29 114 | 71 72 | 137 5 | 137 5 | 61 77 | 67.3 |
| All(554) | 97 457 | 286 268 | 402 142 | 495 49 | 177 355 | 61.3 |

Unfavourable prognostic indices were older age, male sex, stage II, B symptoms, Grade II histology, low Hb, low albumin and raised ESR.
Conclusion: The survival for EN NHL varies between sites - the most favourable being head and neck (H&N), the least favourable was CNS The overall survival of locoregional EN NHL was less than to that of nodal NHL. Prognostic indicators for survival were those acknowledged for all NHLs (age, sex, stage, symptoms, histology, grade, Hb, ESR albumin).

### O08

RANDOMIZED STUDY OF VINORELBINE OR VINDESINE WITH CISPLATIN AND MITOMYCIN AS INDUCTION CHEMOTHERAPY IN NSCLC. K. Furuse, M. Kawahara, Y. Nishiwaki, T. Horai, N. Saijo, K. Hasegawa, K. Okishio & H. Niitani, Japan

To evaluate new drugs in combination with cisplatin and mitomycin in advanced stage IIIB and IV non-small cell lung cancer (NSCLC), we conducted a multicenter randomized study. Eligible patients (pts) were stage IIIB or IV NSCLC, performance status (PS) 0 or 1, and were previously untreated. Pts were randomized to receive vinorelbine 25 mg/m$^2$ on day 1 and 8 (arm A) or vindesine 3 mg/m$^2$ on day 1 and 8 (arm B). Cisplatin 80 mg/m$^2$ and mitomycin 8 mg/m$^2$ were given in both groups on day 1, and cycles were repeated every 4 weeks. Results:

| | arm A (n=54) | arm B (n=53) | p value |
|---|---|---|---|
| **PS 0/1** | 15/39 | 16/37 | |
| **Stage IIIB/IV** | 26/28 | 25/28 | |
| **Overall reponse (95% CI)** | 57 % (34-71 %) | 38 % (25-52 %) | 0.04 |
| **Median survival (mo)** | > 12 | 8.0 | 0.017 log-rank |
| **Neutropenia (Grade 3 - 4)** | 11 % - 87 % | 17 % - 77 % | |
| **Thrombopenia (Grade 3 - 4)** | 11 % - 9 % | 21 % - 2 % | |
| **Hepatotoxicity (Grade 3)** | 2 % | 2 % | |

Treatment arms A and B were well balanced in characteristics such as gender, age, histology, stage and PS. One hundred and seven pts were accrued. To date, preliminary response rate is 57 % for arm A and 38 % for arm B. This difference in response rate is significant (p=0.04). Although the median follow-up time is 8.0 months, the median survival time is > 12 months in arm A and significantly longer than 8.0 months in arm B (p=0.017). Toxicities during chemotherapy have been similar and tolerable in both arms. There was one treatment-related death in arm B. These preliminary results suggest that arm A including navelbine is more active then arm B including vindesine. Further study of arm A and concurrent radiotherapy in unresectable stage III is warranted.

### O09

ANALYSIS OF SECOND MALIGNANCY AFTER HODGKIN'S DISEASE. A Horwich[1], A J Swerdlow[2], J A Barber[2], G Vaughan Hudson[3], R K Gupta[4], D Linch[3], D Cunningham[1], T A Lister[4]. Royal Marsden NHS Trust[1], London School of Hygiene & Tropical Medicine[2], BNLI[3], St Bartholomew's Hospital[4].

To analyse second malignancy (SM) risk, especially with respect to age and time after treatment for Hodgkin's disease (HD), we reviewed 5519 patients treated between 1963-1993 within hospitals in the British National Lymphoma Investigation BNLI (n = 3772), The Royal Marsden Hospital (n = 1039) and St Bartholomew's Hospital (n = 708).

There were a total of 322 second malignancies (excluding non-melanoma skin cancer) representing a relative risk (RR) of 2.9 (95% CI 2.6 - 3.2), including 44 leukaemias, RR 14.6 (95% CI 10.6 - 19.5) and 50 non Hodgkin's lymphomas, RR 14.1 (95% CI 10.4 - 18.5).

The risk of SM was higher in patients treated at a younger age, with for example, a RR 26.1 (12.5 - 47.0) in age < 15 years, a RR of 7.0 (5.1 - 9.3) for those 15 - 24 years, and RR of 1.9 (1.6 - 2.4) for those ≥ 55 years. The RR of leukaemia and NHL was highest 5 - 9 years after treatment whereas for solid tumours the RR increased with time after treatment.

We conclude that second malignancies are a significant and long term problem after treatment for HD, with higher risk in younger patients.

### O10

BONE PAIN TRIAL: A PROSPECTIVE RANDOMISED TRIAL COMPARING A SINGLE DOSE OF 8 GY AND A MULTIFRACTION RADIOTHERAPY SCHEDULE IN THE TREATMENT OF METASTATIC BONE PAIN.

(JR Yarnold, Royal Marsden NHS Trust, Sutton, Surrey, UK, on behalf of the Bonepain Trial Group)

**Background:** Between 1992 and 1997, 762 patients with painful bone metastases were randomised to receive either a multifraction regimen or 8Gy single fraction after written informed consent. The primary endpoints of the trial were pain relief at 1 month and duration of pain relief to 12 months as recorded on patient self-assessment questionnaires. These were completed prior to randomisation, at 2 weeks and 4 weeks, monthly until month 6 and then at 8 months, 10 months and 12 months.

**Results:** The median age of the patients at randomisation was 67 (range 20-91) and the ratio of male to female was roughly 1:1. The most common primary sites of cancer were breast (37%), prostate (35%) and lung (12%). The randomised sites of pain that occurred most often were lumbar spine (19%), hip (14%), pelvis (10%) and ribs (10%). Patient compliance with the questionnaire remained in excess of 90% throughout the period of the study.

No statistically significant difference between the two regimens was observed with regards to pain relief; in particular the proportion of patients experiencing pain relief at one month (58% single fraction, 56% multifraction), those experiencing a subsequent return of pain to its original baseline level (34%, 36%) and any pain relief from baseline (74%, 70%).

**Conclusion:** The results indicate that a single fraction of 8Gy is as effective as a multifraction regimen in relieving metastatic bone pain for up to 12 months after treatment.

### O11

EFFECT OF GAP NUMBER AND DURATION ON OUTCOME OF RADIOTHERAPY IN LARYNGEAL CANCER M van Daesdonk, J. Glaholm, D. Moffat, ND James. CRC Institute for Cancer Studies and Queen Elizabeth Hospital, Birmingham.

**Aim:** Treatment interruptions are known to affect the outcome in head and neck cancer. We have investigated the effects of interruptions on disease free survival in patients receiving radical radiotherapy over 3 to 4 weeks rather than 6-6.5 weeks.

**Methods:** The records of all patients who received primary radical radiotherapy for squamous cell carcinoma of the larynx at the Queen Elizabeth Hospital, Birmingham, between 1991 and 1994 were examined. The following items were recorded: age, sex, stage and treatment details, together with dates of recurrence, death and cause of death.

**Results:** A total of 337 patients were treated during the study period. The median age for all patients was 66.16 years. The majority of patients received either 50Gy in 15 fractions or 55Gy in 20 fractions. Actuarial disease free survival at 2 years was 70% for node negative and 23% for node positive patients. 323 patients had at least one gap in treatment. There was no significant effect from prolongation of the overall treatment time (p=0.62) on disease free survival. There was however a significant correlation between the number of gaps and outcome with patients experiencing 3 or 4 gaps in their treatment having significantly worse disease free survival compared to those patients with 0-2 gaps (p=0.049). The overwhelming majority of gaps in treatment were due either to service days (241 interruptions) or to bank holidays (105 interruptions) only 18 interruptions were due to the patient being too ill to attend.

**Conclusions:** For patients receiving radiotherapy using treatment schedules of 3 to 4 weeks, multiple gaps in treatment appear to be more significant than overall treatment time in affecting outcome. This has important implications for the organisation of treatment in busy radiotherapy departments.

### O12

CERVICAL NODE METASTASIS OF UNKNOWN PRIMARY SITE: THE ROYAL MARSDEN HOSPITAL 15 YEAR EXPERIENCE.

A.N.J Tutt, R. A'Hern, J.M. Henk. Head and Neck Unit.
Royal Marsden Hospital NHS Trust. London SW3 6JJ.

Purpose: Prophylactic radiation to sites of potential subclinical primary disease within the pharynx causes substantial morbidity and is controversial. We describe our experience of managing cervical node metastasis with occult primary avoiding pan-pharyngeal irradiation.
Methods: We identified 53 patients, presenting between 1983 and 1998, with cervical metastases, excluding the supraclavicular fossa, in whom no primary was identified. We excluded patients with non Squamous/UCNT histology or those where treatment intent was not curative. We thus included 41 patients, with median follow up of 29 months.
Results: Investigations included EUA in 38 patients, with multiple biopsies in 32, and CT scan in 32. TNM nodal stage was N1 in 8, N2a in 7, N2b in 8, N2c in 1, N3 in 13 and unavailable in 1. Eighteen patients had extracapsular spread (ECS). Three patients had neck dissection alone, 13 had combined treatment and the remainder radical radiotherapy. Of patients receiving radiotherapy the volume was limited to the ipsilateral hemi-neck in 33. Nodal control in the neck was never achieved in 3 patients. A head and neck primary subsequently became apparent in 12 patients occurring in the tongue base in 6, nasopharynx in 2, tonsil in 2 and 1 each in the pyriform fossa and parotid, at a median interval of 51 weeks (range 15-325). These occurred within the radiation field in 2 patients and adjacent to it in 5. Radical treatment to the primary was given in 10 patients. Neck failure occurred in 20 patients, 9 within the radiation field, at a median interval of 59 weeks (range 15-325). One patient died with an uncontrolled head and neck primary, 10 with an uncontrolled neck and 1 with both uncontrolled neck and primary. Three patients had neck disease when lost to follow up. Actuarial overall survival at 3 and 5 years was 63% (95% CI 45% - 64%) and 44% (95% CI 22% - 64%).
Conclusion: Selective avoidance of pharyngeal irradiation after CT, EUA and speculative biopsies is justified but we find the tongue base to be a frequent site of subsequent primary. This site may be better evaluated by MRI / PET imaging. Only one patient died with uncontrolled local disease at the primary site alone. The patient also had metastatic disease. This justifies the approach to management adopted. Uncontrolled neck disease at death is a more frequent problem than lack of control at the primary site.

**Aim**: To prospectively assess the accuracy of treatment set-up in radical radiotherapy for lung cancer.

**Method**: Initial evaluation of portal imaging and verification films proved these methods inappropriate because of inadequate bony landmarks as reference points. Therefore, patients returned to the simulator weekly, during their treatment, for four simulator check radiographs (SCRs). For each SCR, treatment parameters from the original simulator visit were set, without fluoroscopy. Twenty-four patients were studied, and 96 SCRs were taken. Field placement errors (FPEs) were obtained by measuring the displacement of field centres between the SCRs and the corresponding original simulator radiograph (OSR). FPEs in anterior, right-left (RL) and superior-inferior (SI) planes, and coronal rotational errors (CREs) were measured.

**Results**: In the arms down group (ADG) (12 patients with SCLC, treated with parallel opposed fields), mean FPE was 0.43 cm to the right of, and 0.36 cm inferior to, the intended field centre. The mean CRE was 2°, with 40% of the films in this sub-group showing an error of >2°. In the remaining 12 patients (NSCLC) planned using computerised tomography (arms up group, AUG), mean FPE was 0.27cm to the right, and 0.5 cm inferiorly. The mean CRE in this sub-group was 1°, with 23% of the films showing a CRE of >2°. In both patient groups, 28% of the measurements showed a FPE of more than 0.5 cm, and 3% and 4% in the ADG and AUG respectively showed a FPE of more than 1 cm. No statistically significant differences were found between the FPEs in these two groups, although there was an increased tendency for the FPEs to be displaced inferiorly in the AUG. No patient factors predicted for worse outcome in any direction.

**Conclusion**: Despite no immobilisation device being employed, FPE was within clinical tolerance (FPE <=0.5 cm) in 72% of measurements. The obvious concern is in the remaining 28% where the entire planning target volume may fail to receive the tumouricidal dose, compromising tumour control. CRE was worse in the ADG, probably because lateral tatoos are not used for treatment set-up for AP/PA treatment portals. Quantification of FPE is critical for conformal techniques and dose escalation.

### O15

DYNAMIC CONTRAST ENHANCED MAGNETIC RESONANCE SCANNING TO PREDICT OUTCOME AFTER RADIOTHERAPY FOR HEAD & NECK CANCER.

P J Hoskin, MI Saunders, K Goodchild, MEB Powell, J Taylor and H Baddeley. Mount Vernon Hospital, Northwood, Middx HA6 2RN, U.K.

Dynamic contrast enhanced magnetic resonance imaging (DCEMR) uses rapid acquisition of scan images following intravenous administration of gadolinium with 30 images per scan slice being obtained over a 219 second period. This enables the uptake of gadolinium during its distribution phase into an area of interest to be monitored and subsequently analysed.

This technique has been applied to 13 patients with advanced head and neck cancer with DCEMR performed before and on completion of a course of accelerated radiotherapy. The scans have been analysed in terms of maximum relative signal intensity (rSI) using the pre-contrast signal intensity as baseline, slope of the rSI against time curve and the time taken to reach maximum rSI. The scan parameters have been related to tumour outcome after radiotherapy (RT).

Local tumour control was inversely related to the maximum rSI within a region of interest encompassing the tumour on a post-RT scan but not on a pre-RT scan, with durable tumour control being seen in those tumours with an rSI of < 8. The slope of the signal intensity *versus* time curve on the pre-RT scan sequence was also inversely related to control, the threshold value being < 7.5.

The results imply that tumours with a slow rate of gadolinium uptake prior to RT or reduced gadolinium uptake at the end of RT are those most sensitive to treatment with accelerated radiation. Gadolinium uptake reflects both vascularity and vessel permeability. Tumours which have high rates of perfusion pre-RT and intact vascular networks post-RT are less likely to respond to RT and these features predict local failure.

This technique may be able to select those patients for elective salvage surgery immediately after RT and also predict those patients likely to have sustained local control with no further treatment.

### O16

VIRTUAL SIMULATION;-A REALITY IN SHEFFIELD.
S.Pledge[1], C.Anthony[1],G Brown[1] J.Johnson[3], S Kingstone,J.Conway[2], M.Robinson[1], [1]YCR. Dept. Clin Oncology, [2]Physics Dept., [3]Radiotherapy Dept, Weston Park Hosp. Whitham Rd, Sheffield S10 2SJ, UK.
Introduction: The combination of CT simulation, digitally reconstructed radiographs and portal imaging has the potential to reduce the need for a second conventional simulation when planning simple or complex fields arrangements. Its role is so far unproven.
Aim: To report 1) the accuracy and feasibility of virtual simulation for the palliative treatment of lung cancer and in planned treatment of laryngeal cancer. 2) the advantages and disadvantages of Virtual simulation after a year of use.
Methods: 557 CT localisations were carried out during 1997 including 272 pelvic, 134 chest, 68 head and neck and 32 lymphoma patients.
Studies:
1) Assessment of the impact of respiration on target volume of conventionally simulated lung cancer. palliation, showing no effect.
2) Assessment of different methods of defining fiducials or anatomical landmarks on conventional simulator films, Digitally Reconstructed Radiographs and Portal Images. This facilitates the registration and quantitative comparison of the setup achieved on conventional and virtual simulators, and the treatment machine using PIPS2 software.
Using these techniques we have found that it was possible to replicate a conventionally simulated palliative lung portal to an accuracy of 1mm (SD 2.5 mm) in 10 patients.
Virtual simulation of a 6 radical laryngeal carcinoma patients was accurate to 1.8 mm (SD 1.5 mm).
Our current studies are looking at the impact the CT data has on field size, symptomatic response and toxicity in palliative lung radiotherapy, and at the effect administration of intravenous contrast when scanning has on the gross tumour volume chosen by the clinician.
When the planning CT and diagnostic CTs of lymphoma patients were compared the Gross Target Volume (GTV) was altered in 20% of cases, usually because of disease progression between scans. A technique for planning mantle and inverted Y radiotherapy will be discussed.
Head and neck cases have been successfully simulated.
Conclusions The machine has rapidly been accepted by clinicians as a tool for CT. On the basis of our early experience the scope for improving treatment accuracy and specificity is great.

### O17

STEREOTACTICALLY GUIDED CONFORMAL RADIOTHERAPY FOR MENINGIOMAS - TECHNIQUE AND EARLY RESULTS.
F.H. Saran, J. Perks, H. Alheit, A.P. Warrington, I. Rosenberg, C. Beardmore, M.Brada, 'Department of Radiotherapy and Physics, The Royal Marsden NHS Trust Hospital, Downs Road, Sutton, Surrey SM2 5PT
***Purpose:*** Describe the rationale, the technique and early results of stereotactically guided conformal radiotherapy (SCRT) for meningiomas.
***Material and methods:*** From 7/93 to 11/97 24 patients (median age: 56 yrs., range 28-72) with meningiomas were treated with SCRT. Patients were treated stereotactically in a GTC frame to a target volume (PTV) defined as gross tumour volume (GTV) plus a 0.5-1 cm margin in 3D to doses of 50-55 Gy/ 30-33#/ 6-6½ weeks, using fixed conformal fields. Follow up was 1 - 36 months (median 7 months). Results were compared to a cohort of meningioma patients treated with conventional radiotherapy (RT) (reported previously).
***Results:*** Treatment was delivered with 3 or 4 non-coplanar conformal fixed fields using beams-eye-view (BEV) shaped lead alloy blocks or a multi-leaf collimator, as the optimum stereotactic technique to minimise the dose to normal brain. Median GTV was 21.7 $cm^3$ (range: 4.4-183). Two patients experienced early side effects. 1 - 3 year progression free and overall survival are 100%.
***Conclusion:*** SCRT is a feasible high precision technique associated with minimal acute toxicity and tumour control comparable to that seen with conventional RT. While it is possible to show technical benefit of SCRT over conventionally delivered RT, clinical advantage in terms of tumour control and long term morbidity will need to be demonstrated in long term studies before accepting SCRT as the routine therapy for inoperable residual and recurrent meningiomas.

### O18 INTERLEUKIN-2, INTERFERON-ALPHA AND 5-FLUOROURACIL IN METASTATIC RENAL CARCINOMA: Results of a modified 'Hannover' regimen

*J.M.Abraham, D.Mort, D.Morrey, M.D.Mason.*
*Velindre Hospital, Cardiff, UK*

Encouraging results are reported[1] using the sequential combination of Interleukin-2 (IL-2), Interferon -alpha (IFN), and 5-Fluorouracil (5-FU), in the treatment of metastatic renal carcinoma. In view of the toxicity and cost of this regimen, we embarked on a modification which omitted the lower dose IL-2 prescribed during weeks 2 and 3. The modified regimen consisted of an 8 week course of treatment. During weeks 1 and 4, 3 subcutaneous (sc) IFN at 6MU/m$^2$ was given once weekly, combined with twice daily sc IL-2 at 10MU/m$^2$ for 3 consecutive days. In weeks 2 and 3, sc IFN at 9MU/m$^2$ was delivered thrice weekly. Finally, weeks 5 to 8 combined thrice weekly sc IFN at 9 MU/m$^2$ with a weekly bolus of IV 5-FU at 750mg/m$^2$.

27 patients were treated. The median age was 52 years (range 34 -73). 15 (55.5%) were identified as a good risk group, 8 (29.7%) as a moderate risk group and 4 (14.8%) as a poor risk group using a previously reported prognostic classification[2]. With a median follow up of 22 months (range 0.75-50), there have been 3 (11.1%) complete responses, 3 (11.1%) partial responses, 8 (29.6%) with stable disease and 12 (44.4%) with progressive disease. 1 patient was not evaluable for response. The overall response rate was 22.2% with a median duration of response of 14 months (range 3 - 22). In those with stable disease, the median progression free duration was comparable at 12 months.

The median survival in the responders was 20 months (range 13-28), 14 months (range 7- 50) in those with stable disease and 7 months (range 0.75-16) in the disease progression group .

In conclusion we demonstrate that the original IL2/IFN/5FU regime can be modified and is still active. Randomised control studies would be needed to demonstrate the full efficacy of this regimen , while comprehensively addressing issues of toxicity and quality of life.

(1) J. Atzpodien et al (1993) Eur.J.Cancer. Vol.29A Suppl.5 ppS6-S8.

(2) M. Jones et al (1993) Cancer Biotherapy. Vol 8(4) pp275-288.

### O19 PHOTODYNAMIC THERAPY FOR RECURRENT PROSTATE CANCER AFTER RADIOTHERAPY: A PILOT STUDY,

TR Arokianathan, DE Whitelaw, WR Lees, SS-C Chang, A Gillams, P Ripley, H Payne, M Emberton and SG Bown, University College London Hospitals.

**INTRODUCTION:** Photodynamic therapy (PDT) involves activation of a photosensitising drug with non-thermal light to produce localised tissue necrosis. We report the early results of PDT for locally recurrent prostate cancer after radiotherapy using the photosensitiser meso-tetrahydroxyphenyl chlorin (mTHPC).

**PATIENTS & METHODS:** Ten patients aged 59-77 years (mean 68 years) with biopsy proven locally recurrent prostate cancer following external beam radiotherapy had PDT as salvage therapy. Pre-treatment serum prostate specific antigen (PSA) ranged from 2.2 to 38 ng/ml (median 11 ng/ml). Patients were photosensitised with 0.15 mg/kg mTHPC intravenously and kept in subdued lighting. Three days later patients were catheterised urethrally and red light from a diode laser (652 nm, 150-200 mW per fibre) was delivered via bare tip optical fibres inserted transperineally into the prostate under trans-rectal ultrasound (TRUS) or magnetic resonance imaging (MRI) guidance (up to 8 fibres per patient; total energy 450 to 1600 J per patient). Contrast-enhanced MRI and TRUS biopsies were performed two months post-treatment. Median follow up is 8 months (range 3-10 months).

**RESULTS:** Treatment was well tolerated. Post-treatment PSA levels fell in 5 patients and 2 attained a PSA nadir <0.5 ng/ml. Post-treatment biopsies showed necrosis and fibrosis in all patients with no evidence of residual tumour in 4 men including those with PSA <0.5 ng/ml. MRI revealed non-enhancing lesions at treatment sites which corresponded to necrosis seen on biopsy. One patient developed incontinence; another developed incontinence which has improved and impotence which has not.

**CONCLUSIONS:** PDT is acceptable to patients. Disease remission can be achieved whilst complications compare favourably with other salvage procedures. Refinements in drug-light dosimetry should improve cancer clearance and reduce complications.

### O20 THE EFFECT OF RECTAL ACTIVITY ON PROSTATE GLAND POSITION DURING CINE MRI OF THE PELVIS: IMPLICATIONS FOR RADIOTHERAPY PLANNING.

**V. S. Khoo[1], A. R. Padhani[2], J. Suckling[2], J. E. Husband[2], M. O. Leach[2], D. P. Dearnaley[1]. [1]Departments of Radiotherapy, and [2]CRC Clinical Magnetic Research Group, Royal Marsden NHS Trust, Sutton, Surrey SM2 5PT.**

**Aim: To determine the effect of rectal activity on the position of the prostate gland as unaccounted prostate movement during radical radiotherapy will impact on the probability of local control and may result in higher toxicity with unnecessary irradiation of surrounding normal tissues.**

**Methods: 55 consecutive patients with prostate cancer were scanned in the axial plane using repeated a T1-weighted spoiled gradient echo sequence (FLASH) every 10 seconds for 7 minutes. 24 patients received bowel relaxants before imaging. Pelvic images were analysed for the degree of rectal distension, for the incidence, magnitude and number of rectal and associated prostate movements.**

**Results: Overall rectal movements were seen in 28 (51%) of patients; in 10 (42%) of those receiving bowel relaxants and in 18 (58%) not receiving bowel relaxants. The incidence of rectal movements correlated with the degree of rectal distension (p=0.0005). Prostate movements were seen in 16 (29%) patients and in 9 (16%) patients, the movements were greater than 5 mm. Overall, individual 86 rectal movements resulted in 33 movements of the prostate in the anterior-posterior direction. The magnitude of these rectal movements correlated well with the degree of observed prostate movements (p<0.001).**

**Conclusion: Cine MRI of the pelvis demonstrates significant rectal activity which influences the prostate gland position over a time period similar to that used for the delivery of daily radiotherapy fractionated regimens. For increased treatment accuracy, methods which allow for real time verification of daily treatment volumes are needed to account for internal organ movements in individual patients.**

### O21 RANDOMIZED TRIAL OF HIGH DOSE CONFORMAL RADIOTHERAPY FOR PROSTATE CANCER: INITIAL REPORT OF ACUTE TOXICITY.

J Wilson, V Khoo, L Meyer, M Bidmead, J Warrington, S Helyer, J Gadd, R Huddart, A Horwich, D Dearnaley. The Royal Marsden NHS Trust & Institute of Cancer Research.

Conformal radiotherapy (CRT) may permit safe dose escalation by decreasing the amount of normal tissues included in the treatment volume. In 1995, we commenced a prospective randomized trial of high vs standard dose CRT in localized prostate cancer. This planned analysis of the first 50 patients in each group documents acute toxicity during and following CRT to 3 months.

Eligible men were considered suitable for radical CRT with T1b-T3 N0 M0 prostate cancer. Initial androgen deprivation with LHRH agonist (and short course cyproterone) was given for 3-6 months prior to CRT. Patients were randomized to receive high dose (74Gy/37Fr/7.5wk) CRT or standard dose (64Gy/32Fr/6.5wk) CRT. All patients received 64Gy to a PTV including prostate (+/- seminal vessicles depending on the risk of involvement) using a margin of 1-1.5cm from GTV to PTV. High dose patients had an additional 10Gy to the prostate alone with no added margin. Acute toxicity was documented using the RTOG system.

Patient characteristics were balanced for the 2 groups. Overall: age (mean 67yr; range 50-82), T stage (21% T1, 48% T2, 31% T3), PSA (mean 17.5ng/mL, range 1-145), grade (19% W-D, 71% M-D, 10% P-D), pre-CRT symptoms (bladder 92% RTOG score=0, rectum 97% score=0).

No difference in maximum recorded acute bladder or rectal toxicity between the 2 groups were found to 18 weeks of follow-up.

**Max Toxicity (% patients with RTOG score)**

| | Bladder | | | | | Rectum | | | |
|---|---|---|---|---|---|---|---|---|---|
| | 0 | 1 | 2 | 3 | 4 | 0 | 1 | 2 | 3 |
| 74Gy CRT (%) | 14 | 38 | 34 | 10 | 4 | 16 | 42 | 42 | 0 |
| 64Gy CRT (%) | 24 | 38 | 26 | 12 | 0 | 24 | 30 | 40 | 6 |
| | p=0.2 (trend $\chi^2$) | | | | | p=0.9 (trend $\chi^2$) | | | |

We conclude that dose escalation with CRT in prostate cancer is not associated with an increase in peak acute toxicity. This study has now been adopted as the first MRC Radiotherapy Working Party Trial (RT-01) and is open for national recruitment.

### O22

REDUCTION OF RADIATION INDUCED RECTAL BLEEDING USING RADICAL CONFORMAL RADIOTHERAPY IN PROSTATE CANCER: A RANDOMISED CONTROL TRIAL D. P. Dearnaley, V. Khoo, A. Norman, L. Meyer, A. Nahum, D. Tait, J. Yarnold, A. Horwich, Institute of Cancer Research & Royal Marsden NHS Trust, Sutton and London

Background: Radical radiotherapy (RT) is commonly used to treat localised prostate cancer. Side-effects limit the dose that can be given, but may relate to the volume of normal tissues irradiated. Conformal RT CFRT by shaping the high dose volume to the prostate reduces the amount of rectum and bladder treated. To evaluate this new technology we have performed a randomised controlled trial comparing conformal and conventional RT.

Methods: We recruited 225 men with prostate cancer who were randomly assigned to treatment with radical RT, to our standard dose of 64 Gy in daily 2 Gy fractions, using either conformal or conventional methods. The primary end-point was the development of late (>3 months after treatment) radiation induced complications which were measured using the RTOG (Radiation Therapy and Oncology Group) physician based scoring method. Disease control end-points were also recorded.

Findings: After a minimum follow-up of 2 years CFRT produced a significant reduction in the development of radiation induced proctitis and bleeding (Grade ≥1, 37% versus 56% p=.0009; Grade ≥2, 5% versus 15% p=.01). No significant differences were found in local or biochemical disease control or overall survival.

Interpretation: Conformal techniques reduced the late radiation side-effects of prostate cancer radiotherapy while maintaining similar efficacy to conventional treatment methods.

### O23

PRELIMINARY RESULTS OF AN MRC TRIAL OF PALLIATIVE RADIOTHERAPY IN ADVANCED BLADDER CANCER. J J Bolger, G Duchesne, G Griffiths & B Uscinska on behalf of MRC Bladder Cancer Working Party, MRC Cancer Trials Office, 5 Shaftesbury Road, Cambridge, CB2 2BW, UK

A multi-centre, randomised controlled trial comparing the efficacy and side effects of two palliative radiotherapy schedules (35 Gy in 10 fractions and 21 Gy in 3 fractions in patients with advanced bladder cancer was conducted. Eligible patients had symptomatic, histologically proven invasive bladder cancer, were considered unsuitable for radical radiotherapy or chemotherapy and had a life expectancy of at least three months. Urinary and bowel symptoms were assessed at pre-treatment, end of treatment and at three months. From November 1992 to November 1997 a total of 500 patients were entered by 20 centres. 352 patients have died. There was found to be no evidence of a difference in survival between radiotherapy schedules (Hazard ratio=1.04 95% CI 0.84-1.28 p-value=0.72 . A total of 428 patients had symptom data at both pre-treatment and at end of treatment assessments and 247 at both pre-treatment and 3 month assessments. There was no evidence that the change of symptoms between pre-treatment and at end of treatment or 3 month assessment were different between the two radiotherapy schedules. Although this is the largest trial conducted of palliative therapy in bladder cancer, modest differences cannot be reliably excluded. Nevertheless, from current data we conclude that there is no evidence of a difference between the two palliative radiotherapy schedules in terms of survival and symptom palliation.

One of the clinical problems in managing patients with prostate cancer is that there is a paucity of markers of early relapse within each tumour stage. The identification of such markers would enable clinicians to determine which patients could be offered watchful waiting versus radical therapy. We have analysed the $(CAG)_N$ and $(GGC)_N$ expressed polymorphic repeats in the androgen receptor (AR) gene in germline DNA in 178 UK Caucasian prostate cancer patients and compared these repeat lengths with geographically matched controls. At presentation 22% of the patients had T1/2 disease, 37% had T3/4, 8% had nodal metastases and 33% had metastatic disease outside the pelvis. As expected, the disease-free survival was significantly improved in stages T1-T4 versus node-positive/distant metastatic disease (p< 0.001) and with decreasing T-stage. DNA from blood lymphocytes from 178 prostate cancer patients and 178 geographically matched controls was submitted to radiolabelled PCR and AR repeat length determined by running the PCR products on polyacrylamide gels with known size markers. Long (>21) CAG repeats significantly increased as the disease stage increased ($X^2$ trend = 7.3, p=0.007). For GGC repeat length, there was no evidence of any stage-associated trend. Patients with T1-T4 disease had a statistically significant longer relapse-free period of, on average, 101 months if their GGC repeat length was ≤16 repeats (49 patients relapsed out of 108; p<0.002). This period was 2.5 times longer than those patients with >16 repeats with the same clinical stage. The same effect was observed if the patients were partitioned by age (<70 v. ≥70 years) or were grouped as organ-confined (T1/T2) or escaped (T3/T4). These data suggest that the use of a germline polymorphic marker may be a useful new prognostic marker for the progression of prostate cancer within each clinical T stage and may be able to determine which patients should be offered earlier radical treatment for localised disease.

### O25

IMMUNOTHERAPY FOR PROSTATE CANCER – IN VITRO STUDIES PJ Atherton, M Gilligan, LS Young, PF Searle, AB Rickinson, ND James. CRC Institute for Cancer Studies, University of Birmingham, Birmingham B15 2TA

**Aim:** To evaluate the ability of prostate cancer cells in culture to act as targets for cell mediated immune responses by studying antigen processing and presentation in prostate cancer cells in vitro.

**Methods:** The prostate cancer cell lines PC3, DU145, LNCaP, TSU-Pr1, JCA1 were studied with LCLs as positive controls for antigen processing. Expression of class 1 and class 2 MHC antigens was examined in all cell types. Antigen processing in 2 cell lines, one positive and one negative for class 1 MHC expression was examined in detail in functional assays.

**Results:** Expression of class 2 MHC antigens was down regulated in all the prostate cancer lines studied. Expression of class 1 MHC antigens was preserved in all of the prostate cancer cell lines except for LNCaP which was down regulated to low levels, was not inducible with interferon but was partially inducible with TNFα. Expression of the antigen transporter protein TAP1 was down regulated in the LNCaP cells. Functional assays either transfecting single EBV-derived genes or adding the corresponding processed peptide sequence were carried out with appropriate specific cytotoxic T lymphocytes. The class 1 positive cell line TSU-Pr1 was able to process the EBV antigen appropriately and was lysed in a specific fashion. In contrast, the LNCaP cells were unable to process a TAP-independent antigen even with the simultaneous transfection of the appropriate HLA haplotype showing that the antigen processing defect in these cells was TAP independent.

**Conclusions:** Some prostate cancer cell lines have intact machinery for the appropriate presentation and processing of antigen although other cell lines such as LNCaP have clear functional deficiencies that cannot be easily overcome. The data does show that at least in some cases and an immunotherapeutic approach to prostate cancer may be feasible.

### O26 LONG TERM FOLLOW-UP AFTER RADIOTHERAPY FOR MUSCLE-INVASIVE BLADDER CANCER

PW Cooke[1], DMA Wallace[2], J Dunn[1], S Bathers[1], T Latief[2], ND James[1].

[1]CRC Institute for Cancer Studies & [2]Queen Elizabeth Hospital, Birmingham.

**Aim**: Muscle-invasive bladder cancer carries a poor prognosis with a five year survival rate of less than 40%. Debate remains as to the optimal form of treatment between primary cystectomy and radiotherapy with salvage surgery, with or without chemotherapy. We report the long-term outcome of patients treated in the West Midlands neoadjuvant study of cisplatinum and radiotherapy.

**Methods:** Patients with biopsy proven muscle invasive bladder cancer of category T2 - T4a NX M0, and WHO performance status 0-2 were entered into a prospective randomised study comparing radiotherapy versus radiotherapy with neoadjuvant cisplatinum between June 1984 and June 1988. Follow-up was by 3 monthly cystoscopy in the first year, 6 monthly the next 2 years and yearly thereafter. Salvage surgery was performed at the discretion of the participating clinician.

**Results:** Median follow-up to 1997 was 11 years with a minimum of 9 years, at which time 29 patients (18%) were still alive. Mean age of patients was 65 years (SD 6.7 years) The median survival was 24 months. There was no difference in survival between the 2 treatment groups ($p=0.77$). Overall cystectomy rate was 24% (radiotherapy alone 20%, combined therapy 28%; $p=0.24$). Median time to cystectomy was 12 months; range 56 days to 10 years.

**Conclusions:** Salvage cystectomy is necessary in a quarter of patients after radiotherapy and this can be needed up to 10 years after treatment. Clinicians should be aware of this and patients should be fully counselled about the prolonged need for surveillance and the persisting risk of salvage surgery when deciding between primary cystectomy and radiotherapy. Ways of improving the local control achieved with radiotherapy are urgently needed.

### O27 A PILOT PHARMACOKINETIC STUDY OF AMSALOG (CI-921) ADMINISTERED ORALLY ON A FIVE DAY SCHEDULE.

D. Fyfe[1], F. Reynaud[2], J. White[1], K. Clayton[1], L. Mason[1], C. Gardner[3], P. J. Woll[1], I. Judson[2], and J. Carmichael[1]. [1]CRC Dept. of Clinical Oncology, Nottingham City Hospital, Nottingham U.K. [2]ICR, Royal Marsden Hospital, London, U.K. [3]CRC, Regents Park, London, U.K.

**Introduction**: Amsalog is a derivative of 9-aminoacridine. Phase I studies using intravenous Amsalog showed the dose limiting toxicity (DLT) to be phlebitis and myelosuppression. Phase II studies using 200 mg/m$^2$ i.v. daily for 5 days showed evidence of activity in large cell lung and prostate cancers. Amsalog is active orally: pre-clinical studies showed 26% bioavailabilty in fed rabbits, 69% in fasted. In view of this, a clinical study of oral bioavailability of Amsalog was performed.

**Patients and Methods:** 19 patients with refractory malignancies were treated in 4 cohorts. Treatment was repeated every 3 weeks. For the first and second cycles, Amsalog was administered intravenously at 200 mg/m$^2$ on day 1, then orally on days 2,4,5, and 6. The first oral dose was 200 mg/m$^2$. Pharmacokinetic studies were performed to assess bioavailability. Dose escalation was based on oral bioavailability and toxicity. In the absence of DLT, and if plasma drug levels after oral administration were below those obtained intravenously, there was intra-patient dose escalation of up to 100% for the second cycle. Patients were then given Amsalog orally on days 1 to 5 for subsequent cycles. If the higher dose was tolerated, the next cohort was treated at this level initially, with a further intra-patient dose escalation for the next cycles.

**Results:** Mean oral bioavailability was 28.4% (median 27.7, range 13.8–53.4%). Toxicities included mild lethargy and nausea.

**Conclusion:** Amsalog is well tolerated orally on a 5 day schedule at doses up to 800 mg/m$^2$.

### O28 APOPTOTIC INDEX AS A PROGNOSTIC FACTOR IN A TRIAL OF PRIMARY MEDICAL THERAPY FOR CARCINOMA OF THE BREAST.

R Gregory, P Ellis, T Powles, M Dowsett. Royal Marsden Hospital, Sutton, UK

Methods: Apoptotic index (AI) was determined immunohistochemically by ISEL (In-Situ-End-Labelling) on paraffin embedded sections from 211 patients randomised in a trial of primary medical therapy (PMT) at the Royal Marsden Hospital. In this trial, patients were randomised to receive either 3 months of chemoendocrine therapy (mitoxantrone 11mg/m2, methotrexate 35mg/m2 to a maximum of 50mg q21 and tamoxifen 20mg od; MMT) prior to surgery - neoadjuvant group (NG); or chemoendocrine therapy in the adjuvant setting - adjuvant group (AG). Therefore, half of the tumours examined in this study had been exposed to 3 months of chemoendocrine therapy.

Results: AI was available on tumours from 96 patients in the NG and 115 patients in the AG. In the NG the median AI was 0.17 (0-2.6) compared to a median of 0.35 (0-3.8) in the AG, this difference is statistically significant p=0.0004. A high AI post treatment (ie >0.17) was associated with a poor response to MMT treatment, p=0.004 and this group of patients also had a significantly worse DFS, p=0.03. AI did not predict for DFS in the group as a whole, or in the adjuvant arm.

Conclusion: AI would appear to carry both prognostic and predictive power in patients receiving neoadjuvant chemoendocrine therapy for primary carcinoma of the breast. In addition, although we have no pre-treatment samples available on these patients AI would appear to be reduced by MMT treatment.

### O29 Biodistribution and pharmacokinetics 111In-DTPA-labelled Stealth liposomes (IDLSL) in patients with solid cancers.

Harrington K[a], Abra R[b], Uster P[b], Stewart S[a]. ICRF Oncology Unit, Hammersmith Hospital, UK[a]. SEQUUS Pharmaceuticals Inc., USA[b].

**Purpose:** To investigate the biodistribution and pharmacokinetics of IDLSL in patients with advanced solid cancers as a means of assessing their potential role as a vehicle for the delivery of cytotoxic drugs and radiosensitising agents.

**Patients and Methods:** 17 patients (5 breast ca, 5 head and neck ca, 4 lung ca, 2 glioma, 1 cervix ca) received 65-107 MBq of IDLSL by intravenous infusion. Blood samples and whole body gamma camera images were taken at 0.5, 4, 24, 48, 72, 96 and 240 hours and sequential 24 hour urine collections were performed for the first 96 hours. SPECT imaging of regions of interest was carried out as indicated. Liposome uptake in tumour and normal organs was estimated from regions of interest on the gamma camera images. In a separate study, 2 patients undergoing excision of head and neck cancers received 27 MBq of IDLSL 48 hrs before surgery. Samples of tumour and normal tissues were obtained at operation.

**Results:**

| | 0.5 h | 4 h | 24 h | 48 h | 72 h | 240 h |
|---|---|---|---|---|---|---|
| **Blood** | 95.0±11.8 | 85.3±9.5 | 70.7±9.2 | 55.5±9.3 | 46.3±9.5 | 4.9±5.1 |
| **Plasma** | 100.9±13.6 | 90.8±13.0 | 59.1±12.1 | 49.0±11.9 | 39.1±11.1 | 4.9±5.4 |

The excretion half-life ($t_{1/2\beta}$) of IDLSL was 76.1 h. Median cumulative urinary excretion of 111In over 96 hours was 19.5 (range 3.5-28.4) % of the injected dose (%ID). Positive tumour images were obtained in 15/17 studies (4/5 breast, 5/5 head & neck, 3/4 bronchus, 2/2 glioma, 1/1 cervix cancer). Tumour liposome uptake estimated from regions of interest on the whole body gamma camera images showed considerable heterogeneity, both between different tumour types and between tumours of the same histological type. Total tumour uptake ranged between 0.5-3.5 %ID, with peak uptake at 72 hrs (mean 1.4±0.9 %ID). Peak normal organ uptakes (%ID) were as follows: liver (4 hrs) 11.7±1.9, spleen (72 hrs) 5.1±2.2, lung (0.5 hrs) 5.6±1.4, kidneys 1.6±0.8. In the 2 patients undergoing surgery, tumour uptake was 8.8 and 15.0 %ID/kg, respectively. Tumour uptake exceeded that in normal mucosa by a mean ratio of 2.4:1.

**Conclusions:** This study confirms that Stealth liposomes effectively target a range of common solid cancers.

### O30 CHANGES IN HUMAN TUMOUR PERFUSION AFTER DMXAA TREATMENT, MONITORED BY DYNAMIC GD-DTPA ENHANCED MRI.

S.M. Galbraith[1], N.J. Taylor[2], G. Rustin[1], H. Baddeley[2]. [1]Cancer Treatment Centre and [2]Paul Strickland Scanner Centre, Mount Vernon Hospital, Northwood, Middx, HA6 2RN, UK.

**Background:** Dimethylxanthenone acetic acid (DMXAA), a novel tumour vascular targeting agent causes a reduction in tumour perfusion leading to response and haemorrhagic necrosis in animal tumours.

**Objective:** To assess whether tumour perfusion is affected in patients participating in the Phase 1 trial of DMXAA.

**Methods:** 3 patients were scanned pre-treatment, 4 hrs and 24 hrs after the first infusion, and after 6 weekly infusions of DMXAA (500mg m$^{-2}$-patient 1, 650mg/m$^2$ -patients 2 and 3). 30 dynamic images were obtained at 12.8s intervals prior to, during and after intravenous injection of Gadolinium-DTPA. The sequential images were analysed for each pixel to generate parameter images of maximum signal intensity time gradient and enhancement. Both these parameters have been used as measures of perfusion. Regions of interest (ROIs) were defined for each dynamic series of scans to encompass the tumour and also for nearby muscle. The difference between the means for the above parameters within these ROIs pre and post treatment was compared using a two-tailed t test.

**Results:** The mean gradient maximum for the first 2 patients' tumours fell by 22% and 30% respectively (p<0.01) by 24hrs and by 37% and 28% (p<0.01) by 6 weeks, but was unchanged in the third patient. The mean enhancement was unchanged in the first patient, but reduced by 16% and 25% respectively (p<0.01) in the next 2 patients. There were no significant changes in maximum gradient or enhancement in underlying muscle. The parameter images illustrated that the changes in blood flow were heterogeneous within the tumours. All 3 patients experienced pain in the tumour site after each infusion.

**Conclusions:** The documented changes in tumour blood flow demonstrate a biological effect of DMXAA in humans, and provide evidence supporting the concept of tumour vascular targeting. Further patients are being recruited for blood flow imaging.

### O32 PHARMACOKINETIC (PK) INTERACTION BETWEEN 5-FLUOROURACIL (5-FU) AND OXALIPLATIN (L-OHP).

D Papamichael, S P Joel, *M T Seymour, *F Richards, M Bowerbank, M L Slevin. *Departments of Medical Oncology at St.Bartholomew's Hospital, London and *Cookridge Hospital, Leeds, UK.*

***Background***: The combination of 5-FU and L-OHP has exhibited clinical synergistic activity. We searched for a possible PK interaction to explain this synergy. ***Patients/Methods***: In the context of a randomized phase II-III clinical trial comparing 5-FU/Leucovorin (LV) with or without L-OHP, 16 patients with advanced/metastatic colorectal cancer- 8 in each arm -have been studied to date. The regimen comprised LV (200mg/m$^2$) i.v. infusion over 2 hours immediately followed by 5-FU (400mg/m$^2$) iv bolus then 5-FU (600mg/m$^2$) by iv infusion over 22 hours, all repeated on day 2 and given fortnightly; L-OHP was given as a 2 hour infusion together with LV, day 1. PK sampling was performed on day 1 of the 1$^{st}$ cycle, with samples taken at time 0 (pre-bolus), 5, 10, 15, 20, 30, 45 minutes and 1, 1.5, 2, 2.5, 3, 6 and 22 hours. Samples were immediately cold-spun and the plasma stored at -40$^0$C. 5-FU was analysed by reverse phase HPLC. Kinetic data were fitted to a one compartment model with two elimination routes, one linear and the other non-linear; the latter with $K_m$ fixed at 15 uM representing the principle catabolic pathway via dihydropyrimidine dehydrogenase. For each patient the distribution volume ($V_d$), maximum non-linear elimination rate ($V_{max}$) and linear elimination rate ($k_{elim}$) were derived using a non-linear iterative procedure. $AUC_{0-22}$ was determined using a trapezoidal method. ***Results***: 5-FU PK parameters with and without L-OHP are shown in the table (mean ± SD).

| | $V_{d\ (L)}$ | $V_{max\ (Um\ hr^{-1})}$ | $k_{elim (hr^{-1})}$ | $AUC_{(uM\ hr^{-1})}$ |
|---|---|---|---|---|
| 5-FU alone | 20.8±9.3 | 362±215 | 3.5±0.9 | 90.3±28.6 |
| *p (Mann-Whitt.)* | *0.81* | *0.48* | *0.05* | *0.05* |
| 5-FU/L-OHP | 22.6±8.7 | 274±199 | 5.9±3.1 | 61.3±17.0 |

***Conclusions:*** L-OHP as used in this regimen, significantly affects 5-FU PK, seen as reduced AUC and increased linear elimination but not non-linear clearance. We hypothesise that L-OHP enhances the anabolic pathway for 5-FU.

### O33 HIGH DOSE CHEMOTHERAPY (HDCT) WITH PBSC SUPPORT FOR EITHER POOR PROGNOSIS OR RELAPSED NON-SEMINOMATOUS GERM CELL TUMOURS (NSGCT)

Wilkinson P M, Decatris M, Heron J, Morgenstern G and Welch R S.
Christie Hospital, Depts of Clin Oncology and Haematology, Manchester.

Whilst there is general agreement as to the treatment of good prognosis NSGCT, the treatment of advanced and relapsed disease remains a challenge. The efficacy of HDCT with PBSC support was evaluated in 16 pts with advanced disease (as defined by the MRC prognostic analysis) and in 8 pts who had relapsed following standard therapy. All pts received 3 or 4 cycles of induction therapy before receiving HDCT (Etopside 450mg/M2, Cyclophosphamide 35mg/kg and carboplatin AUC*30). Pts were harvested after the first cycle; the CD34 count had to be >1x10$^6$/kg and GM-CFC >5x10$^4$/kg to allow HDCT to proceed.

**Results**

| | N | Age | Primary Gonad | Primary Other | Duration of Neutropenia | Days in Hospital | Outcome CR |
|---|---|---|---|---|---|---|---|
| Poor prognosis | 16 | 29(15-45) | 12 | 4 | 9.2(9-12) | 22(17-50) | 12(75%) |
| Relapsed | 8 | 28(15-45) | 5 | 3 | 9.4(5-11) | 21.5(13-36) | 4(50%) |

Whilst the majority of pts became febrile during the period of neutropenia there was no life threatening toxicity and no toxic deaths were recorded. The response in *de-novo* pts was 75% falling to 50% in relapsed pts.
HDCT is a safe treatment option for newly diagnosed pts; its role in relapsed disease requires further studies.

### O34 POSSIBLE ADVERSE EFFECTS OF CURATIVE CHEMOTHERAPY ON BONE MASS IN MEN: USE OF BONE ULTRASOUND TO EVALUATE BONE ARCHITECTURE.

J. Zekri and R.E. Coleman, YCR Department of Clinical Oncology, Weston Park Hospital, Sheffield.

Chemotherapy is known to have long-term adverse effects on skeletal health, particularly in women due to effects on endocrine function with induction of premature ovarian failure. However, little is known about the possible direct effects of cytotoxic drugs on bone cell function and consequent effects on bone mass and structural integrity.

We have screened 114 men on long term follow-up after combination chemotherapy for testicular tumours (n=42), lymphoma (n=67) and other tumours (n=5) using bone ultrasound attenuation (BUA) measured at the calcaneus; a quick, safe and simple method for evaluating bone mass which has been shown to correlate well with the risk of osteoporotic hip fracture. Three measurements of BUA measured in the non dominant foot were averaged and compared to both a large database of age-matched normal men and 49 age-matched cancer controls who had not received chemotherapy. 99 men had received 'standard dose' chemotherapy and 15 had also received 'high dose' myeloablative chemotherapy supported by autologous stem cell rescue.

25/114 (22%) men had BUA T scores >2 S.D. below the age matched mean for normal subjects compared with only 5/49 cancer controls (10%). After adjustment for age, exercise and steroid intake by multiple linear regression, we found a small adverse effect of chemotherapy on BUA. This was most marked and reached statistical significance in men under the age of 30 (n=35; p=0.031), particularly after high dose chemotherapy (n=6; p=0.029). Men aged 30-50 showed the least reduction in BUA from normal; this corresponds with the age around the attainment of peak bone mass and before the phase of natural bone loss.

These data suggest that chemotherapy may have direct non-endocrine mediated effects on bone mass. Further investigation, particularly in young men using bone densitometry and biochemical assessment of bone metabolism is indicated to assess the risk of chemotherapy-induced osteoporosis.

**O35** DOES CENTRAL NERVOUS SYSTEM CHEMOPROPHYLAXIS HAVE A ROLE IN THE MANAGEMENT OF GESTATIONAL TROPHOBLASTIC DISEASE? A M Gillespie[1], N Siddiqui[2], R E Coleman[1] and B W Hancock[1]. [1]Trophoblastic Disease Centre, Yorkshire Cancer Research Dept. Clinical Oncology, Weston Park Hospital, Sheffield, UK. [2]Dept. Obstetrics & Gynaecology, Southern General Hospital, Glasgow, UK.

The role of prophylactic chemotherapy for cerebral micrometastatic disease in persistent trophoblastic disease (PTD) is unclear. The aim of this project was to evaluate the outcome of patients with lung metastases, who at some specialist centres would be deemed to be at high risk of developing central nervous system (CNS) metastases and receive prophylactic intrathecal (IT) chemotherapy. At our centre IT therapy is reserved for those patients with confirmed CNS involvement as determined by imaging or a high (>1:60) cerebrospinal fluid to serum hCG ratio.

Methods: All patients with gestational trophoblastic disease (GTD) registered from 1987-1996 were included in this retrospective analysis. 195 patients with PTD were treated during this time period, median age 27 years (range 16-49 years). The extent of pulmonary metastases was determined by imaging investigations.

Results: 69 patients with pulmonary metastases were identified. 51 patients were classified low risk and 18 patients high risk according to the modified Charing Cross criteria. 66/69 patients have successfully completed their treatment (median follow-up 62 months, range 14-128 months). Two patients had established cerebral metastases, one being successfully treated and the other dying despite multiple therapeutic interventions. One other patient developed a CNS metastasis during treatment compromised by patient non-compliance. Follow-up is ongoing.

Conclusion: Restriction of IT treatment to those patients with established cerebral metastases appears to be safe and effective, and the use of CNS chemoprophylaxis based on the presence of multiple pulmonary metastases is unnecessary.

### O36

IS CLINICAL UNCERTAINTY SUFFICIENT AMONGST BREAST CANCER SURGEONS FOR THE IMPLEMENTATION OF A RANDOMISED TRIAL OF HRT IN BREAST CANCER PATIENTS?

J Marsden[1] JM Bliss[2] [1]Dept. of Surgery, St Heliers Hospital, Surrey, UK and [2]Section of Epidemiology, Institute of Cancer Research, Sutton, UK.

**Introduction:** Breast cancer patients experience oestrogen deficiency symptoms as a result of natural menopause or as side-effects of adjuvant therapy (reported incidence of 66%). Randomised trials assessing HRT in such patients are urgently needed and a UK trial is planned. We wanted to determine how breast cancer surgeons' currently treated symptomatic patients and their views on HRT.
**Method:** One page postal questionnaire sent to 250 designated breast cancer surgeons (BASO Breast Group listing).
**Results:** Response rate was 49%(122/250). Most surgeons who responded, 88%(107/122), treated more than 50 patients/year. Median estimated proportion of patients suffering oestrogen deficiency symptoms was 25% (range 2%-100%) and was not influenced by number of breast cancer patients/year. 77%(94/122) had previously prescribed HRT, usually for relief of severe symptoms when alternatives had been ineffective, only 11%(14/122) stating that ER status would affect their decision. In answer to the question 'Do you believe that HRT may have a role in breast cancer recurrence?', 52%(64/122) said 'yes', 19%(23/122) said 'no' and 24%(29/122) wrote that they 'didn't know'. HRT alternatives considered 'safe' included low dose progestins and topical oestrogens. 21%(26/122) did not prescribe alternatives or refer patients for gynaecological advice. A randomised trial was felt to be ethical by 74%(90/122) but some envisaged difficulties with organisation and questioned the level of interest from patients.
**Conclusions:** The incidence of oestrogen deficiency symptoms in breast cancer patients is underestimated and use of HRT and alternative therapies is not evidence based. There is uncertainty over the risks of HRT and it is encouraging that the majority of surgeons believe a randomised trial to be ethical.

### O37

NEURAL NETWORKS ARE SUPERIOR TO THE TRADITIONAL PROGNOSTIC INDICES FOR BREAST CANCER PATIENTS.

P.J.Drew, P.Magee, L.Bottaci, M.Menon, K.Heer, G.S.Duthie, J.N.Fox, P.J.Carleton, J.R.T.Monson, M.J.Kerin The University of Hull Academic Surgical Unit, Castle Hill Hospital, Hull HU16 5JQ

An artificial neural networks (ANN) is a form of artificial intelligence that is able to learn non-linear causal relationships between data sets and therefore has a theoretical advantage over the traditional linear statistical methods used in the construction of prognostic indices. We therefore compared the ability of ANN's to predict outcome in breast cancer with the established Nottingham prognostic index (NPI).
Data on 460 patients collected retrospectively on the standard BASO database was used to train and validate the ANN. As with the Nottingham prognostic index the size, grade and lymph node status were used as the input nodes with a variable number of neurones utilised in the hidden layer and a simple positive/negative output neurone. The ANN was trained to predict death at one year on 435 patients and then validated on data from 25 randomly selected patients which had been removed prior to training. The ANN's predictions were then compared to the patients' Nottingham prognostic group.
The ANN achieved an overall accuracy of 84% (95% C.I.0.70-0.98) for the prediction of death at one year. The positive predictive value of the network's predictions was 1.0 and the negative predictive value 0.83. In contrast, of the patients in the same validation set, 38% of patients in the moderate-risk group were dead at one year as compared to only 33% of patients in the high-risk group.
Artificial Neural Networks are able to predict outcome for breast cancer patients with greater accuracy than the traditional prognostic indices and particularly for the majority of patients in the "moderate" risk group.

### O38

DYNAMIC MAGNETIC RESONANCE MAMMOGRAPHY ALONE IS SUPERIOR TO TRIPLE ASSESSMENT IN SYMPTOMATIC BREAST DISEASE

S.Chatterjee, P.J.Drew, J.N.Fox, P.J.Carelton, L.Turnbull, J.Read, J.R.T.Monson, M.J.Kerin, Academic Surgical Unit, Castle Hill Hospital, Hull

The accepted gold standard of triple assessment of breast lesions i.e. clinical examination, fine needle aspiration cytology (FNA) and mammography does not always establish a diagnosis and surgical biopsy is therefore required. Contrast enhanced dynamic magnetic resonance mammography (MRM) represents an emerging alternative diagnostic modality. The aim of this study was to compare the diagnostic accuracy of MRM and standard triple assessment in the evaluation of symptomatic breast lesions.
Two hundred and eighty three symptomatic patients (median age 51, range 20-80) were recruited. The 112 patients who did not undergo operation have been followed up clinically and radiologically for a for a median of 20 months (IQR 18-24 months). For detection of malignancy were: clinical examination 84.73%, mammography 86.26%, FNA 80.3%, triple assessment 99.23% and MRM 99.23%. Histologically confirmed multifocal disease was detected pre-operatively in 40 patients by MRM but only 9 (22.5%) on mammography. The specificity for the diagnosis of benign disease was: clinical examination 83.11%, ultrasound 88.88%, mammography 86.36%, FNA 95.0%, triple assessment 77.27% and MRM 98.7%.
MRM is far superior to the traditional triple assessment for the evaluation of symptomatic breast disease, and thus facilitates a significant reduction in the biopsy rate to rule out cancer.

### O39

**RECURRENT BREAST CANCER DETECTED USING DOPPLER ULTRASOUND, MRI AND MAMMOGRAPHY.**

E. C. Whipp, C. Devrell, M. Vaidya, M Halliwell, P. Goddard, C. Wakeley , E. Kutt, J. Brennan and *P Barrett-Lee. United Bristol Healthcare Trust, Bristol BS1 6SY, UK *Velindre Hospital , Cardiff CF4 7XL, UK

**Objective:** To establish the value of mammography, Doppler ultrasound and gadolinium enhanced MRI for the detection of recurrent breast cancer in the previously irradiated breast.

**Methods:** 104 patients with breast induration following adjuvant or neo-adjuvant radiotherapy following wide local excision, hormone and/or chemotherapy and radiotherapy were investigated with mammography, Doppler ultrasound and MRI imaging with gadolinium contrast. Fine needle aspirates were performed in any suspicious case.

**Results:**. In the whole group there have been 20 proven recurrences. The mean follow-up period was 30 months (range 18 - 48 months). The sensitivity rates were: MRI 88%; Doppler 80%; mammography 42%; the specificities were 82%, 76% and 85% respectively. The false negative reports (MRI 2, Doppler 4 and mammography 7) overlapped so that every recurrence was detected by at least one modality. i.e. 100% when using both MRI and Doppler ultrasound.

**Conclusions**: Both ultrasound and MRI detect the neovasculature of recurrent breast cancer, with MRI being more sensitive and more specific. Mammography is relatively insensitive to malignant changes in the post-operative and treated breast. These patients were all at increased risk of local recurrence making a 100% sensitive detection important in their management.

### O40 A RANDOMISED STUDY COMPARING 3-WEEKLY EPIRUBICIN, CYCLOPHOSPHAMIDE AND CONTINUOUS INFUSION 5-FU (ECF) TO A 2-WEEKLY SCHEDULE WITH G-CSF IN ADVANCED BREAST CANCER.

R.H De Boer, T.G Eisen, P.A Ellis, S.R.D Johnston, G Walsh, and I.E Smith. Dept of Medicine, Royal Marsden NHS Trust, Fulham Rd, London, SW3.

Granulocyte-Colony Stimulating Factor (G-CSF) allows cycles of conventional bolus chemotherapy to be accelerated with reduction in treatment time and a boost in dose-intensity[1]. Theoretically, the same approach could be hazardous with infusional chemotherapy, since G-CSF-stimulated neutrophil proliferation would be occurring in the face of continuous S-phase active 5-FU. We therefore carried out a phase II randomised study comparing standard 3-weekly infusional ECF with an accelerated 2-weekly schedule with G-CSF support, in patients with advanced breast cancer. Patients received day 1 epirubicin 60mg/m$^2$ and cyclophosphamide 600mg/m$^2$ each cycle, and 5-FU 200mg/m$^2$/day continuous infusion throughout. G-CSF 300mcg/day s/c (day 10-12), was given each accelerated cycle. 21 patients were randomised (10 accelerated). 6 patients in the standard arm and 1 patient in the accelerated arm were previously untreated. Results showed that there were no treatment delays secondary to inadequate neutrophil or platelet recovery, and the median day 1 neutrophil counts for each cycle were higher in the accelerated arm [5.0 x 10$^9$/L (range 3.0-9.0) vs 3.0 x 10$^9$/L (range 2.3-4.3)]. Neutropenic fever occurred in 3/11 patients (3-weekly) vs 4/10 (2-weekly), grade 3 stomatitis in 2/11 vs 3/10, and grade 3 planter-palmar erythema in 1 patient in each. 88% of the planned standard chemotherapy cycles and 85% of the planned accelerated cycles were given. The planned and given dose intensity was as shown:

| | Cyclophos. | Epirubicin | 5FU |
|---|---|---|---|
| 3W Planned Dose Intensity | 200mg/m$^2$/wk =1.0 | 20mg/m$^2$/wk =1.0 | 1400mg/m$^2$/wk =1.0 |
| 3W Given DI | 0.99 | 0.99 | 0.95 |
| 2W Planned DI | 300mg/m$^2$/wk = 1.5 | 30mg/m$^2$/wk =1.5 | 1400mg/m$^2$/wk =1.0 |
| 2W Given DI | 1.47 | 1.47 | 0.97 |

We conclude that accelerated infusional ECF with G-CSF support is feasible and well tolerated with rapid haematological recovery. An overall 30% increase in dose intensity is acheived, while treatment time is reduced by 33%.

[1]Bisset, D., Jodrell, D., Harnett, A.N., Habeshaw, T., Kaye, S.B., Evans, D., Williams, M., and Canney, P.A. (1995) Phase I study of accelerated FEC with G-CSF (Lenograstim) support. *Br J Ca.* 71, 1279-1282

### O41 CLASSIFICATION OF BREAST DISEASE WITH MRI USING NEURAL NETWORKS

A.J. Knowles, B. Issa, S. Burton, P. Gibbs, G.P. Liney, and L.W. Turnbull, MRI Centre, Hull Royal Infirmary, Hull, HU3 2JZ

**Introduction** Due to overlap in the morphological appearance of benign and malignant lesions on post-contrast scanning, dynamic contrast enhanced MR imaging (DCE-MRI) has been used to aid differentiation. This study investigates the accuracy of neural networks to analyse DCE-MRI, compared to an experienced radiologist.
**Methods** DCE-MRI was performed on 103 patients (69 malignant, 34 benign). Twenty-five images were acquired with a temporal resolution of 11.6 sec. Regions of interest were drawn around the entire abnormality and time-course data for each pixel presented to a variety of neural network architectures. The radiologist then examined both the time course data and the post-contrast images separately and then in combination.
**Results** The probabilistic neural network achieved the best classification with an accuracy of 90%, compared with 76% for the radiologist utilising the time course graphs alone. With the addition of morphological information, the accuracy of the radiologist increased to 89% for the post-contrast images and 97% for the post-contrast images and time course data combined.
**Conclusions** Neural networks offer a reliable, fast and operator independent method for analysing DCE-MRI data. They appear to provide a comparable accuracy to a radiologist utilising post contrast images alone.

### O42 ASSESSMENT OF DIAGNOSTIC STEREOTACTIC EXCISION OF MAMMOGRAPHIC ABNORMALITIES

M Menon, PJ Drew, MJ Imrie, JN Fox, PJ Carleton, JRT Monson, MJ Kerin
The University of Hull Academic Surgical Unit,Castle Hill Hosp., Hull

AIM: The drawbacks of breast screening include unnecessary intervention in patients with benign breast disease and the lack of accurate preoperative diagnosis in patients with screen detected malignancy. We are prospectively assessing the impact of the ABBI system (Auto Suture, USA) which allows stereotactic biopsy and excision of screen detected lesions.
METHOD: Non palpable occult mammographic abnormalities were evaluated on the Advanced Breast Biopsy Instrumentation (ABBI) System. All procedures were carried out under local anaesthetic. All data was collected prospectively.
RESULTS: To date 100 patients have undergone 30 ABBI excisions and 70 core biopsies.11 excisions were malignant, 7 DCIS and 12 benign (including 2 radial scars). Only 4 of the 7 patient with DCIS had residual in-situ disease on further open procedure. Of the 6 fibroadenomata 3 were suspicious mass lesions not seen on ultrasound, the rest were microcalcifications. Median duration of the procedure was 70 minutes (range 35-110). Histology of the core biopsies revealed malignancy in 9, DCIS in 17 (of which 4 were invasive ductal on further open biopsy) and benign in 44 (2 had invasive disease on wire mark-up biopsy. The procedure was well tolerated in all patients with no complications.
CONCLUSION: the ABBI system provides a well tolerated accurate diagnostic intervention for patients with screen detected breast lesions. It facilitates accurate pre-operative diagnosis and may have a potential for therapeutic applications in patients with very small screen detected cancers.

### O43 A COMPARISON OF THE CARE RECEIVED BY WOMEN WITH BREAST CANCER LIVING IN AFFLUENT AND DEPRIVED AREAS,

U Macleod[1], CJ Twelves[2], S Ross[3], C Gillis[4], GCM Watt[1], [1] Dept of General Practice, University of Glasgow, Woodside Health Centre, Barr Street, Glasgow, G20 7LR, [2]CRC Dept of Medical Oncology, [3]Health Services Research Unit, University of Aberdeen, [4]West of Scotland Cancer Surveillance Unit.

**Aim**: To describe and compare the presentation and pattern of care received by women with breast cancer living in affluent and deprived areas.

**Methods**: Case note review of the hospital and general practice case records of women who were diagnosed with breast cancer in Greater Glasgow Health Board in 1992 and 1993, and who lived in Deprivation categories (Carstairs' Index) 1,2 (least deprived, n = 157)and 6,7 (most deprived, n = 264)at time of diagnosis.

**Results:** Deprivation did not affect the pathological prognostic factors at time of presentation with primary breast cancer. However, more women from deprived areas presented with locally advanced or metastatic disease (15.4% *v* 6.4%, $X^2$ = 7.42, p = 0.006). The time from GP's letter to clinic visit was shorter in women from affluent areas (affluent: median 6 days, IQR 1 to 14; deprived: median 7 days, IQR 4 to 20, Z = -2.89, p = 0.004), and the time to surgery from clinic visit was also shorter ( affluent: median 15 days, IQR 9 to 24, deprived: median 17 days, IQR 11 to 28, Z = -2.10, p=0.036). Consultation patterns in general practice by the second year after diagnosis showed women in deprived areas consulting more frequently than women in affluent areas (consulting >12 times per year: 27.0% *v* 15.9% $X^2$ = 12.67, p = 0.027).

**Conclusions:** In this study, women living in deprived areas were more likely to present with large, advanced cancers than women living in affluent areas, to wait longer to be seen at the clinic and longer for surgery. By 2 years after diagnosis, more women in deprived areas continued to consult in excess of once a month in primary care.

### O044

**Biodistribution and pharmacokinetics of 111In-DTPA-labelled stealth liposomes (IDLSL) in a human tumour xenograft model.** K Harrington[a], G Rowlinson-Busza[a], P Uster[b], R Abra[b], S Stewart[a]. ICRF Oncology Unit, Hammersmith Hospital, UK[a]. SEQUUS Pharmaceuticals Inc., USA[b].

**Purpose:** To study the biodistribution and pharmacokinetics of IDLSL in female nude mice bearing the human KB head and neck squamous cell cancer tumour xenograft (HNSCCXT) in order to examine possible applications of Stealth liposome-targetted anti-cancer therapies.

**Materials and Methods:** KB tumour cells were grown in RPMI-1640 medium containing 100 U/ml penicillin and 100 μg/ml streptomycin, supplemented with 10% foetal calf serum at 37°C in a humidified atmosphere of 5% $CO_2$ in air. HNSCCXT were established by injecting 5 x $10^6$ cells subcutaneously in the flank of nude mice. Tumour-bearing mice received an intravenous injection of either 100 μl IDLSL or 100 μl $^{111}$In-DTPA, each containing 0.37 MBq of radioactivity. Mice were dissected at specific time-points and a wide range of tissues were taken and weighed and their content of radioactivity was determined by counting them in a gamma counter. Standards of the injectate were counted to correct for physical decay of the radioisotope.

**Results:** The $t_{1/2\alpha}$ and $t_{1/2\beta}$ of IDLSL were 1.1 and 10.3 h, respectively. Tumour uptake was maximal at 24 h at 5.5 ±3.0 % ID/g. Significant reticulo-endothelial system uptake was demonstrated with 19.3 ±2.8 and 18.8 ±4.2 % ID/g at 24 h in the liver and spleen, respectively. Other sites of appreciable deposition were the kidney, skin, female reproductive tract and to a lesser extent the gastrointestinal tract. There was no indication of cumulative deposition of IDLSL in the lung, central nervous system, musculoskeletal system, heart or adrenal glands. The $t_{1/2\alpha}$ and $t_{1/2\beta}$ of unencapsulated $^{111}$In-DTPA were 5 minutes and 1.1 h, respectively, with no evidence of accumulation in tumour or any normal tissue.

**Conclusions:** (1). Stealth liposomes target HNSCCXT effectively with high levels of deposition. (2). Encapsulation of $^{111}$In-DTPA within a Stealth liposomal matrix dramatically alters its biodistribution and pharmacokinetics. (3). Stealth liposome entrapment of cytotoxic drugs and radiosensitisers offers the possibility of developing novel targeted anti-cancer therapies.

### O045

MODIFICATION OF HUMAN TUMOUR BLOOD FLOW USING PENTOXIFYLLINE AND CARBOGEN.

K A Goodchild[1], SA Hill[2], DJ Chaplin[2], PJ Hoskin[1] and MI Saunders.[1] [1]Mount Vernon Hospital, Northwood, HA6 2RN. [2]Gray Laboratory Cancer Research Trust, PO Box 100, Northwood, HA6 2JR

Introduction Anticancer treatments require well oxygenated tumour cells for maximum effect. Methods to improve human tumour blood flow thereby increasing oxygenation may be an important means of enhancing therapy. In animal tumours pentoxifylline has been reported to increase oxygenation, blood flow and radiosensitivity. Improvement in oxygenation in human tumours has also been shown after administration of pentoxifylline and carbogen (95%O2,5%CO2).

Aim To study the effect of pentoxifylline and carbogen on human tumour perfusion.

Methods Microregional tumour perfusion was measured in superficial tumours using laser Doppler microprobes in 15 patients with histologically proven malignancy. Patients received a single oral dose of either 1200mg or 1600mg of pentoxifylline. Two hours later, at the time of peak drug levels, carbogen was administered and blood flow was recorded using up to 6 microprobes for 30 minutes in total; 10 minutes pre-carbogen, 10 minutes during carbogen and 10 minutes post-carbogen.

Results Although there was marked heterogeneity in response, both between tumours and between different microregions within a single tumour, there was an overall increase in relative laser Doppler flow during carbogen breathing in patients pre-treated with pentoxifylline at both dose levels. No patient showed a decrease in tumour perfusion. Previous studies have shown that carbogen alone may cause either increases or decreases in laser Doppler flux, such that no overall change is apparent.

Conclusions Pentoxifylline in combination with carbogen can increase blood flow in human tumours. This may provide a potential therapeutic gain when used in combination with radiation or chemotherapy.

### O046

IN VITRO ULTRAFRACTIONATION OVERCOMES RADIORESISTANCE IN GLIOMA CELLS. S.C.Short and M.C.Joiner, Gray Laboratory Cancer Research Trust, Mount Vernon Hospital, Northwood, Middx HA6 2JR.

Low-dose hypersensitivity has been demonstrated in a series of 14 different radioresistant human tumour cell lines. It produces increased cell kill per gray at very low doses. We have used one of these, T98G, a human glioblastoma cell line to explore whether this can be exploited in a fractionated regime. Single dose data using T98G suggest that a dose of 0.4 Gy produces marked hypersensitivity. The effect of consecutive 0.4 Gy doses at varying time intervals *in vitro*, suggests that a second hypersensitive response can be obtained if the second dose is given after a four hour interval. We have extended this to look at the effect of three consecutive 0.4 Gy doses at four hour intervals and have shown that cell killing is significantly greater than after a single dose of 1.2 Gy ($p<0.01$). Further, over a period of 5 days we have compared 0.4 Gy 3 times a day at four hour intervals to 1.2 Gy once each day. The ultrafractionated regime produced significantly increased cell kill as measured by clonogenic survival ($p<0.01$). This is contrary to the LQ prediction and supports the idea that low-dose hypersensitivity can be exploited in a fractionated regime and may prove useful in the treatment of radioresistant tumours.

### O047

FERTILITY AFTER IODINE-131 THERAPY FOR THYROID CANCER, L Vini, B. Pratt, A. Al-Saadi, V.R. McCready, C. Harmer. Thyroid Unit, Royal Marsden NHS Trust, Downs Road, Sutton, SM2 5PT, UK.

Aim: This is a retrospective review to assess fertility following treatment with radioiodine in patients with differentiated thyroid cancer. Material: 391 patients (297 women and 94 men) under the age of 40 at the time of treatment were identified. All had well differentiated thyroid carcinoma (298 papillary and 93 follicular) initially treated with total thyroidectomy. Only one dose of $^{131}$I (3GBq) was given to ablate thyroid remnants in 261 patients; the remainder received more than one dose (cumulative activity 8.5-47 GBq) for residual, recurrent or metastatic disease. Information is available for 252 female and 81 male patients. Results: Menstrual changes lasting 2-8 months were reported by 29 women. 198 children were born from 161 female patients and 68 children were fathered by 48 male patients. Four premature births and 3 spontaneous abortions were registered. Only one female has failed to conceive. Malformations have not been reported. The remaining patients had no wish to have children. Conclusions: The risk of damage to the gonads of patients treated with $^{131}$I appears to be low and lacks clinical impact. Radioiodine therapy is a safe procedure in this aspect.

### O48 DNA DAMAGE AS A PREDICTOR OF NORMAL TISSUE RESPONSE TO RADIATION.

AE Kiltie, AJ Ryan, R Swindell, JBP Barber, B Magee, CML West, JH Hendry. Section of Genome Damage and Repair, Paterson Institute for Cancer Research, Christie Hospital NHS Trust, Manchester M20 4BX.

Fibroblast clonogenic assays of intrinsic cell radiosensitivity have been developed which are predictive for normal tissue late responses, but which are too time consuming to be of routine clinical use prior to radiotherapy. As a relationship between fibroblast clonogenic radiosensitivity and residual radiation-induced DNA double strand breaks (dsb) as studied by PFGE has been shown by a number of groups, a study was performed to establish whether this measure of DNA damage was predictive for normal tissue responses to radiation.

Patients treated for breast cancer nine to 15 years previously, with radiotherapy following wide local excision, were studied using a modification of the LENT SOMA score for breast cancer. A 5 mm punch biopsy was taken from the buttock. Fibroblasts from the lower dermis were grown in tissue culture and assayed using PFGE. The normalised values for the fraction of DNA released following 150 Gy were from 4.1% to 12.4%, representing a 3.0-fold range of values. There were weak but significant correlations between the assay result and the fibrosis score ($r=0.46$; $p=0.003$), the combined fibrosis and retraction score ($r=0.45$; $p=0.004$), and the overall LENT score ($r=0.43$; $p=0.006$). Measurement of residual DNA dsb may be of use in the future, probably in combination with other assays, to predict normal tissue reactions after radiotherapy.

This work was funded by the Christie Hospital Endowment Fund and the Cancer Research Campaign.

### O49 HIGH TUMOUR ANGIOGENESIS CORRELATES WITH POOR SURVIVAL IN CERVIX TUMOURS

R Cooper, J Logue, S Davidson, R Hunter, D Wilks, C West

Christie CRC Research Centre, Manchester, UK

<u>Purpose:</u> To determine the relationship between tumour angiogenesis and outcome in 111 patients with stage Ib-IIIb carcinoma of the cervix treated by radiotherapy.
<u>Methods:</u> Formalin fixed paraffin embedded random tumour biopsies were stained using immunohistochemistry for anti-Factor VIII-related antigen. Vascularity was measured by scoring the distance from a random point to the nearest microvessel within the tumour (DTCMV) and by counting the number of microvessels in areas of neovascularisation (IMD).
<u>Results:</u> The level of tumour angiogenesis was a significant prognostic factor for outcome. Patients with IMD counts below the median had a 5-year survival level of 65% compared 50% for those with counts above the median ($p=0.038$). High vascularity as measured by both IMD and DTCMV was also associated with increased local relapse ($p=0.028$ and $p=0.060$, respectively). There was a trend towards improved metastases-free survival for patients with less well vascularised tumours but this was not significant for either method. There was no significant correlation between either DTCMV or IMD and tumour stage, grade or volume.
<u>Conclusion:</u> This large study on a homogeneously treated group of patients indicates that a high degree of tumour angiogenesis is associated with poor prognosis in carcinoma of the cervix treated by radiotherapy.

Supported by the Cancer Research Campaign.

### O50 TUMOUR RADIOSENSITIVITY IS AN INDEPENDENT PROGNOSTIC FACTOR IN CERVIX CANCER

SE Davidson[1], CML West[2], SA Roberts[3], RD Hunter[1],

Christie CRC Research Centre, Manchester M20 4BX.

The prognostic value of pre-treatment measurements of tumour radiosensitivity (surviving fraction at 2 Gy, $SF_2$) in 128 patients with stage I - III cervical carcinomas undergoing radiotherapy was studied. The median follow-up time was 47 months. In a univariate analysis stratifying patients according to the median value, radiosensitivity was a significant prognostic factor for overall survival, local control and metastasis-free survival. The 5-year survival rate for patients whose tumours had $SF_2$ below the median was 81% and was significantly greater than the rate of 51% for those with $SF_2$ values above the median. In bivariate analyses, $SF_2$ was shown to be independent of disease stage, tumour grade, patient age, colony-forming efficiency and tumour diameter. In a multivariate analysis, radiosensitivity was the most important variable and, after allowing for this, only stage was a significant independent predictor of treatment outcome. These data indicate that for patients treated with radiotherapy for cervical carcinoma, pre-treatment tumour radiosensitivity is an important prognostic factor and contributes to prognosis independently of other established and putative parameters.

This work was supported by the Cancer Research Campaign, UK.

### O51 CELLULAR RESPONSE TO IONISING RADIATION IN PATIENTS WITH MUTATIONS IN THE *BRCA1* GENE.

TJ McMillan, J Peacock, R Eeles, HD Platte, A. Pearson, J Tawn, P Daniel, R Berry, Department of Biological Sciences, Lancaster University, Lancaster Radiation Research Department, Institute of Cancer Research, Cotswold Road, Sutton, Surrey, Radiotherapy Department, Royal Marsden Hospital, Downs Road, Sutton, Surrey, Westlakes Research Institute, Moor Row, Whitehaven, Cumbria

The aim of this study is to establish whether individuals carrying mutations in the *BRCA1* gene have an increased susceptibility to the mutagenic and cytotoxic effects of ionising radiation. This is a possible concern due to the influence of other breast-cancer predisposing genetic changes (e.g. TP53 and ATM mutations) being associated with abnormal responses to DNA damage, the reported radiation sensitivity of brca1 knock-out mice and the known association of the BRCA1 protein with proteins involved in DNA damage processing.
We have examined the sensitivity to the killing effects of high- and low-dose rate irradiation in cells from 19 individuals who are members of 5 families with *BRCA1* mutations. From the initial phase of the study we have seen that cells with mutant *BRCA1* do not have an extreme sensitivity to the killing effect of radiation, though their sensitivity parameters do fall at the sensitive end of the normal range. In addition we are examining other responses to radiation including cell cycle perturbations and p53 induction in these cells as potential indicators of sub-lethal damage. The progress on these responses will be reported.

### O52 ANTI-ANGIOGENIC GENE THERAPY

K. Fife[1], L. Buluwela[1], A. Miller[2], R.C. Coombes[1], M. Bower[1]. Charing Cross and Westminster Medical School[1] and Department of Chemistry, Imperial College[2], London, UK.

Angiogenesis is recognised as an important target for anti-cancer therapy, as neoangiogenesis is vital for the initiation and growth of solid tumours. A novel gene therapy approach to antiangiogenesis is described. Briefly, human endothelial cell lines were transfected with the Herpes simplex virus thymidine kinase (HSV-tk) gene using a liposome vector. Exposure of transfected cells to ganciclovir (GCV) resulted in greatly preferential cytotoxicity to the HSV-tk transfected cells as compared with controls.

Human endothelial cell transfection is known to be inefficient. Liposomes were chosen as the vector for transfection as they have a better safety profile than viruses. Optimisation of transfection was performed, using the SV40βgal reporter plasmid, with three commercially available liposomes (Transfectam, Tfx-50 and DOTAP) and three supplied by the Department of Chemistry, Imperial College, London (DC-Chol/DOPE, ACHx/DC-Chol/DOPE and ACO/DC-Chol/DOPE). The greatest transfection efficiencies were obtained using the cationic liposome DC-Chol/DOPE with values of 5.8% for HUV-EC-C and 9.5% for ECV304 human endothelial cell lines. These are amongst the best reported figures for non-viral endothelial transfection, and DC-Chol/DOPE has the advantage of having been used in human gene therapy trials (Caplen et al., 1995).

DC-Chol/DOPE was therefore used to transfect cells with HSV-tk. Co-transfection with a puromycin resistance plasmid was performed to allow antibiotic selection of transfected cells. When exposed to GCV for six days, HSV-tk transfected HUV-EC-C cells demonstrated cytotoxicity $3\log_{10}$ greater than controls, and ECV304 cells $2\text{-}3\log_{10}$ greater. The bystander effect was also demonstrated with HUV-EC-C cells. These results show that non-viral transfection of human endothelial cells with a 'suicide gene' is a feasible and potentially valuable approach to anti-angiogenesis.

Caplen, N. *et al.* 1995. *Nature Med.* 1, 39-46.

### O53 RADIOIMMUNOTHERAPY OF B-CELL LYMPHOMAS WITH IODINE-131 LABELLED ANTIBODIES: DETERMINANTS OF SUCCESSFUL THERAPY.

**T. M. Illidge; R.French; R.Reid; and M. J. Glennie. Lymphoma Research Laboratory, Southampton General Hospital, Southampton S016 6YD.**

In this study report we report for the first time the in vivo modulation, biodistribution, and radioimmunotherapy (RIT) of a range of B-cell specific antibodies in syngeneic B-cell lymphoma animal models. Initial biodistribution studies enabled accurate organ dose predictions to be made directly comparing two radiolabelled antibodies directed against B-cell antigen targets that internalise to a greater and lesser degree. The important determinants of effective radioimmunotherapy in the mouse B cell lymphocytic lymphoma $BCL_1$ and the B cell tumour A31, were evaluated by comparing the effects of antibodies against CD19, CD22, CD40, the invariant chain Ii (CD74), MHC Class II, versus tumour specific anti-idiotype and isotype matched control antibodies with and without conjugated Iodine-131. When treated early in the disease a very high therapeutic efficacy has been demonstrated (>90% cure), targeting highly expressed antigens that internalise poorly using low doses (2 MBq) of $^{131}$I anti-CD74 and $^{131}$I anti-Class II antibodies. With larger tumour burdens, delivering an increased dose of radioactivity to the tumour bearing organs by the selection of a highly expressed poorly modulating B cell antigen target (MHC Class II, CD74), has been shown to be of lesser importance than the selection of an antibody against a B cell antigen where irradiation and antibody response appear to be synergistic (Immunoglobulin idiotype and anti-CD40). These results using low dose RIT have important implications for clinical RIT strategies in NHL; strongly suggesting the success of this approach is much more than targeted irradiation and that antibody and irradiation can be synergistic in the induction of apoptosis.

(This work was supported by the Cancer Research Campaign, UK.)

### O54 BAX ε AND ϕ; NOVEL mRNA SPLICE VARIANTS OF THE PRO-APOPTOTIC GENE

A.L. Thomas, C.G. Price, S. Martin, J. Carmichael, J.C. Murray, CRC Dept. of Clinical Oncology, City Hospital, Hucknall Road, Nottingham NG5 1PB.

The ability of a tumour cell to withstand apoptosis is emerging as an important factor in the development of drug resistance in tumour cells. Apoptosis in response to genotoxic stress is mediated by the Bcl-2 family of proteins, which includes Bax. The *bax* gene comprises 6 exons and demonstrates a complex pattern of alternative mRNA splicing. By RT-PCR, we detected the presence of two new variants, *bax* ε and *bax* ϕ, in peripheral blood leucocytes, and a variety of normal and tumour cell lines. Sequencing of the PCR products indicates that *bax* ε lacks exons 2 and 3, while *bax* ϕ contains an extra 49 bp between exons 5 and 6, arising from a partial deletion of intron 5. *Bax* ε has a predicted protein of only 1kDa and does not contain any known functional domains, whereas *bax* ϕ has a predicted weight of 24kDa and contains all domains except the transmembrane domain. Western blotting and *in vitro* transcription-translation have been used to characterise the protein products. Currently we are using a transient expression system to ascertain the functional significance of these novel genes.

### O55 DNA VACCINATION WITH A scFv OF THE ANTI-IDIOTYPE ANTIBODY 105AD7 INDUCES A $TH_1$ IMMUNE RESPONSE.

V. Potter, I. Spendlove, and LG Durrant. CRC Department of Clinical Oncology, City Hospital, Hucknall Road, Nottingham, NG5 1PB, UK.

105AD7 is a human monoclonal anti-idiotype antibody vaccine which mimics T cell epitopes on the tumour antigen 791Tgp72 and has been shown to elicit specific anti-tumour T cell immune responses in animal models and clinical studies. We are investigating whether a scFv DNA construct could mimic the activity of the 105AD7 antibody vaccine and enhance cytotoxic T cell responses. The cDNA encoding the heavy and light chains of 105AD7 has been sequenced and we have produced a scFv of the V regions joined by a 15mer linker. Protein of the correct molecular weight has been detected in the cell lysate of *E.coli* transfected with the scFv and in a eukaryotic *in vitro* expression system. Mice were vaccinated with 100μg of the scFv DNA via the IM and ID routes. As a control the well characterised pCMV-S plasmid which encodes for the HBsAg was used. Both plasmids, irrespective of route of vaccination have produced specific antibody responses, to their respective antigen, which are predominantly of the IgG2a subtype. In contrast vaccination with the 105AD7 antibody and HBsAg recombinant protein produced an IgG1 response. The predominant IgG2a antibody response produced by DNA vaccination is a feature of $TH_1$ immune responses, associated with cell mediated cytotoxicity. Protein vaccines, however, characteristically produce strong antibody ($TH_2$) responses. The antibody titres produced by the 105AD7 scFv are consistently lower than those against HBsAg, this may be explained by the variable and often poor secretion, as seen in the prokaryotic system. In order to overcome this problem DNA constructs that produce a scFv dimer and trimer are being evaluated as these have been shown to be more easily secreted in many systems.

### O56 PHASE II TRIAL OF THE BISPECIFIC ANTIBODY MDX-H210 (ANTI-HER2/NEU X ANTI-CD64) COMBINED WITH GM-CSF IN PATIENTS WITH ADVANCED PROSTATE AND RENAL CELL CARCINOMAS THAT EXPRESS HER2/NEU.

N. James[1], P. Atherton[1], A. Koletsky[2], N. Tchekmedyian[3] and R. Curnow[4]. [1]CRC Institute for Cancer Studies, Birmingham B15 2TH UK. [2]Intercenter Cancer Research Group, Florida, [3]Pacific Shores Medical Group, California. [4]Medarex, Inc., New Jersey, USA.

**Aim:** To evaluate the bispecific antibody MDX-H210 (which combines a recognition site for HER-2/neu with a triggering sequence for the high affinity IgG Fc receptor CD64) combined with GM-CSF in patients with hormone refractory metastatic prostate or locally advanced or metastatic renal cell cancers that express HER2/neu.

**Methods:** 23 patients (5 renal, 18 prostate cancer) were treated with GM-CSF (5 µg/kg/day by subcutaneous injection for 4 days), followed by MDX-H210 (15 mg/m$^2$ by intravenous infusion), repeated weekly for 3 weeks. Patients with stable disease or better with no dose limiting toxicity were eligible for a second cycle of treatment.

**Results:** We have seen 2 objective responses in patients with renal cancer: a 52% reduction in the tumour (liver metastases as large as 10 x 12 cm) in one patient and a 49% reduction in the size of a pulmonary metastasis with clearing of non-measurable lesions in another patient. We have seen 5 objective responses (>50% decline in PSA) in patients with prostate cancer: 104 to <0.1, 20 to < 0.1, 118 to 11, 872 to 207 and 126 to 58 ng/ml. Quality of life improvements have been noted. Toxicity with GM-CSF was mild (worst NCI-CTC grade 2). Little or no additional toxicity was seen in the majority of patients after infusion of MDX-H210; however, in responders, grade 3 toxicity was seen in both renal cancer patients and 1 prostate cancer patient.

**Conclusions:** We believe this is the first evidence of clinical response to immunotherapy in patients with hormone refractory prostate cancer, and indicates promising potential for MDX-H210 plus GM-CSF in the treatment of patients with renal and prostate cancer.

### O57 CD40 EXPRESSION IN BLADDER CANCER

PW Cooke[1], ND James[1], R Ganesan[2], DMA Wallace[2], A Burton[1] and LSYoung[1]. [1]CRC Institute for Cancer Studies & [2]Queen Elizabeth Hospital, Birmingham.

**Introduction:** The CD40 receptor is expressed in many immune cell types and is known to play a central role in both humoral and T-cell mediated immunity, being a subject of intense research interest in recent years. It is also expressed on a variety of carcinomas and may therefore be of biological significance in the development and treatment of cancer.

**Methods:** The expression of CD40 was examined immunohistochemically in a series of 131 bladder tumours, and the correlation with known prognostic markers and clinical outcome assessed.

**Results:** 79% of the tumours were CD40-positive, with a highly significant association with both lower stage and lower grade ($p<0.001$). Superficial tumours expressed CD40 in 89% of specimens compared to 61% seen in invasive tumours, and in contrast to normal urothelium, which was mainly CD40-negative. CD40 expression was not related to any other clinico-pathological variable including lymphocyte infiltration, Bcl-2, or p53 expression, nor was it an independent prognostic marker.

**Conclusions:** The lack of relationship with Bcl-2 staining, which is normally seen in basal epidermal cells, may indicate alternative or abnormal CD40-mediated cell differentiation mechanisms. The diffuse expression seen in superficial bladder cancer may account for it's clinically less aggressive behaviour and is likely to be an important factor in the excellent clinical response seen to BCG immunotherapy. It also raises the possibility of the future development of CD40/CD40 ligand-based immunotherapy for bladder cancer.

### O58 PROMOTER CONSTRUCTS FOR PROSTATE CANCER GENE THERAPY

J Latham, PF Searle, V Mautner, ND James
CRC Institute for Cancer Studies, University of Birmingham, Edgbaston, Birmingham B15 2TA

**Aim:** To construct tissue specific promoters based on prostate specific antigen for use in human gene therapy.

**Method:** A range of luciferase reporter vectors, using sequences from the proximal 5′ PSA and cytomegalovirus (CMV) promoters for expression control, were constructed and their tissue specific nature was explored in a variety of cell types. Assays were standardised using a CMVIE promoter driven β-Galactosidase reporter vector.

**Results:** The minimal 5′ 650bp PSA promoter showed only low level non-specific expression. Placing two PSA promoters in tandem also gave low levels of expression. The addition of the CMVIE enhancer upstream of the PSA promoter increased luciferase expression substantially but overrode any tissue specific control the PSA promoter may have possessed. However, placing a 1400bp PSA enhancer sequence upstream of the minimal PSA promoter increased expression in a tissue-dependent manner. A further enhancer placed in tandem upstream greatly increased expression while retaining tissue-specific control. Furthermore the level of expression from both the single and tandem enhancer constructs can be increased 100- fold above their basal levels when induced with the androgen, dihydrotestosterone.

PSA enhancer tandem PSA-promoter driven *ntr*, B7-1 and GFP adenoviruses have been made, and investigations into the tissue specific nature of these viral vectors in a range of tissue types including primary prostate tissue is underway.

**Conclusions:** Prostate tissue specific constructs can be made in vitro and further evaluation using viral vectors is now underway as a prelude to clinical studies.

### O59 INFILTRATION OF CERVICAL CANCER TISSUE WITH HUMAN PAPILLOMAVIRUS-SPECIFIC CYTOTOXIC T-LYMPHOCYTES

EML Evans, S Man, AS Evans*, LK Borysiewicz; Depts. of Medicine & Gynaecology*, University of Wales College of Medicine, Cardiff.

Introduction Cytotoxic T-lymphocytes (CTL) specific for high-risk HPVs (16 and 18) have recently been found in the peripheral blood of cervical cancer (CC) patients (Borysiewicz et al, 1996; Ressing et al, 1996) but their relevance at sites of disease is unclear. Two HPV-16 E7 peptides were used to map the distribution of HPV-specific CTL in peripheral blood (PBL), draining lymph nodes (LNL) and tumours (TIL) of CC patients.

Methods HPV and control peptides were used to restimulate PBL, LNL and TIL *in vitro*. Peptide-pulsed dendritic cells were used to induce primary responses in control subjects. Limiting dilution analysis in the absence of specific antigen stimulation identified CTL lysing target cells infected with a recombinant vaccinia virus encoding modified forms of HPV-16 and -18 E6/E7 protein sequences (TA-HPV) (Borysiewicz et al, 1996).

Results We identified HLA-A*0201-restricted HPV-16 E7 peptide-specific CTL, in peripheral blood (4/5), draining lymph nodes (3/4) and tumours (1/3) of CC patients. All peptide-specific lines also recognised target cells infected with the recombinant vaccinia virus, TA-HPV. In 4/4 CC patients, the frequency of CTL specific for TA-HPV was found to be higher in tumours and lymph nodes compared with peripheral blood. HPV-specific CTL were not identified in any of 7 healthy controls, but primary responses could be generated by peptide-pulsed dendritic cells (4/4). HPV 16 E7 peptide-specific clones were expanded *in vitro*, and analysis showed restricted TCRBV segment usage, although CDR3 regions were not oligoclonal.

Conclusion Although the role of virus-specific TIL *in vivo* has not been determined, this study raises the possibility that CTL induced by vaccination or adoptively transferred can infiltrate sites of disease.

References Borysiewicz LK et al (1996) *Lancet*. 347, pp1523-1527; Ressing ME et al (1996) *Cancer Res*. 56, pp582-588.

**O60** PHASE I STUDY OF ASCENDING DOSES OF THE CYTOTOXIC IMMUNOCONJUGATE CMB-401 (hCTMO1 - CALICHEAMICIN) IN PATIENTS WITH EPITHELIAL OVARIAN CANCER.

A M Gillespie[1], T J Broadhead[2], S Y Chan[2], J Owen[1], A P Farnsworth[3], M Sopwith[3] and R E Coleman[1]. 1. Yorkshire Cancer Research Dept. of Clinical Oncology, Weston Park Hospital, Sheffield, UK. 2. Dept. Clinical Oncology, City Hospital, Nottingham, UK. 3. Celltech Therapeutics Ltd, Slough, UK.

We have performed a Phase I study of the cytotoxic immunoconjugate CMB-401 (hCTMO1 - calicheamicin) in women with epithelial ovarian cancer (EOC). hCTMO1 is a genetically engineered human antibody directed against polymorphic epithelial mucin which binds preferentially to EOC (tumour: blood ratio 8:1 - *Cancer Res 1996;56:5179-5185).* The objectives of this two centre study were to identify end-organ toxicities and to establish maximum tolerated dose (MTD). Tumour response was also monitored.

Methods: 34 patients were recruited with satisfactory WHO performance status, aged 20-75 and with progressive EOC not amenable to platinum/standard therapy. Patients received up to four cycles of a dual infusion of 35mg/m$^2$ hCTMO1 'pre-dose' followed by ascending doses of CMB-401 - a regimen which minimises drug uptake in normal tissues whilst enhancing delivery to the EOC.

Results: CMB-401 dosing commenced at 2mg/m$^2$ and progressed via seven cohorts to 16mg/m$^2$. CMB-401 was generally well tolerated. Transient malaise and emesis occurred, necessitating routine prophylaxis. WHO grade 3/4 toxicities, irrespective of causality, included: haematological (anaemia 21%, granulocytopenia 9%, thrombocytopenia 9%); liver and renal (transaminases 3%, alkaline phosphatase 6%, urea 3%); sepsis 3%; haemorrhage 6%, nausea/vomiting 76%; pulmonary 6%, conscious stage 6%. MTD was defined at 16mg/m$^2$ by malaise, haematological toxicities and the experience of gastro-intestinal haemorrhage. During the study four patients had a greater than 50% reduction in CA125, and four patients had imaging evidence of reduction in tumour bulk.

Conclusion: CMB-401 appears to have an acceptable toxicity profile with initial evidence of activity against EOC. Development will continue in a Phase II study.

### O61 EPIRUBICIN (E), CISPLATIN (C), UFT (U) - "ECU" IN UPPER GI CANCER: LOSING THE PUMP, BUT KEEPING THE EFFICACY.

*MT Seymour, JT Dent, *D Papamichael, HE Cresswell & G Wilson. ICRF Cancer Medicine Research Unit, Cookridge Hospital. Leeds & *St Bartholomew's Hospital, London.*

**Background**: "ECF":- 3-weekly E & C plus protracted venous infusion (PVI) fluorouracil (F), is a highly active combination with superior activity to FAMTX in gastroesophageal cancer *(JCO 15:261)* and impressive response rates in breast cancer *(JCO 12:1259)* and other solid tumours; however, the inclusion of PVI F adds to its morbidity and cost. Oral UFT generates plasma 5FU levels similar to PVI F *(Proc ASCO 14:1498)*. We therefore performed a dose-escalation pilot study of "ECU", in which F is replaced by oral UFT, at escalating doses, together with weekly folinic acid (FA). **Method**: 30 previously untreated patients (pts) with advanced upper GI cancer were entered (73% gastroesoph.; 17% biliary.; 10% pancr.). 29/30 had PS 0-1; median age, 58. All received E, 50 mg/m$^2$ and C, 60 mg/m$^2$ on day 1, and FA 45 mg p.o. on d1, 8 & 15 of a 21-day cycle. UFT was taken 12 hourly, d1-21, at escalating doses in cohorts ranging from 150 to 325 mg/m$^2$/d. Up to 8 cycles were given. Pts with measurable disease were assessed by CT or MRI every 12 weeks. **Results**: Of 12 pts started at, or escalated to, 265 mg/m$^2$/d UFT, 4 required dose reduction, making this the MTD. Of 8 pts treated at 325 mg/m$^2$/d, 4 required UFT reductions. Most UFT dose reductions were for mild (CTC grade 2) but persistent fatigue, nausea or diarrhea, rather than severe acute toxicity. However, one pt each at 265 and 325 mg/m$^2$/d had grade 4 toxicity (ileus/small bowel obstruction) which may be related to UFT. Only 2 episodes of gr 3 neutropenia were recorded, and no neutropenic sepsis. 17 of the 30 pts on this pilot study had assessable disease. Of these there were 2 CR and 6 PR (RR = 47% overall, 57% in gastroesoph. cancer). **Conclusion**: "ECU" is generally well tolerated at the defined MTD, with activity comparable to ECF in gastroesophageal cancer. It is a promising alternative to ECF and deserves further, randomised evaluation.

### O62 OXALIPLATIN, 5-FLUOROURACIL (FU) & FOLINIC ACID (FA) FIRST-LINE COMBINATION THERAPY IN COLORECTAL CANCER: RESULTS OF A PHASE III TRIAL.

*MT Seymour[1], J Cassidy[2], C Wilson[3], D Papamichael[4], M Homerin[5], A Figer[6] & A de Gramont[7]. [1]Cookridge Hosp-Leeds; [2]Univ. Hosp-Aberdeen; [3]Addenbrookes Hosp-Cambridge; [4]St Bartholomew's Hosp-London; [5]Debiopharm-Paris; [6]Med. Cen. Petach Tikva-Israel; [7]Hôp St Antoine-Paris.*

**Background:** Bolus + infusion FU with FA, given over 48 hrs each fortnight, is a popular and well-tolerated treatment for advanced colorectal cancer and, in a previous trial, gave superior results to "standard" Mayo 5-day bolus FU/FA *(JCO 15:808, '97)*. This study was designed to evaluate the addition of oxaliplatin to this schedule. **Patients/Methods:** From Aug '95 -Jul '97, 420 pts with measurable colorectal cancer at 37 centres were randomised to receive either **[A]** FA 200 mg/m$^2$ over 2 hrs, then FU 400 mg/m$^2$ bolus and 600 mg/m$^2$ over 22 hrs, days 1+2 every 2 weeks; or **[B]** the same FU/FA regimen plus oxaliplatin 85 mg/m$^2$ over 2 hrs on day 1. Treatment was continued until progression or toxicity. Randomisation was well balanced for primary site (colon in 72%[A] vs 70%[B]), performance status (= 0 in 48%[A] vs 43%[B]), number of metastatic sites (>1 in 60%[A] vs 57%[B]) and prior adjuvant chemo (20% in both arms). Tumour measurements were made every 8 weeks and all responses subjected to independent radiological review. **Results:** Median PFS (all pts) is currently **27.8 wk [A]** vs **39.6 wk [B]**. A protocol-planned interim analysis on the first 200 pts shows response rates (RR) of **26% [A]** vs **57% [B]** (p<0.05). Both regimens were well tolerated. Toxicity analysis in the first 200 pts shows slightly higher rates in [B] of CTC grade (gr) 3-4 diarrhoea (12% vs 2%), vomiting (7% vs 1%) or stomatitis (6% vs 0%). Asymptomatic neutropenia was commoner in [B]: gr 3 in 41% (vs 6%) pts, and gr 4 in 13% (vs 0%), but severe infection occurred only 1% in both groups. 15% of pts in [B] experienced gr 3 neurosensory symptoms. **Conclusion:** The good activity and the excellent tolerability of the control schedule is confirmed. The addition of oxaliplatin substantially enhances its activity with a definite but easily manageable increase in toxicity. Final analysis of all 420 pts will be presented at the meeting.

### O63 ARTIFICIAL NEURAL NETWORKS FOR THE PREDICTION OF OUTCOME FOLLOWING SURGERY FOR GASTRIC CANCER

P.J.Drew, L.Bottaci, P.Magee, *I.M.C.Macintyre, M.J.Kerin, J.R.T.Monson, G.S.Duthie The University of Hull Academic Surgical Unit, Castle Hill Hospital & *Western General Infirmary, Edinburgh

An artificial neural network (ANN) is a form of computer artificial intelligence that is able to learn non-linear causal relationships between established data and use this knowledge to answer questions about data to which it has not been exposed. We compared a neural network analysis with the traditional statistical approach for the prediction of outcome following surgery for gastric cancer.

ANN's were designed with 60 input variables in the first layer, 0,2,3,4,5,6,7,15,20,25 or 30 hidden "neurones" in the second layer and a simple positive/negative output. Data from 183 patients was utilised: 133 to train and 50 to validate each ANN for the prediction of death at 12 months. The neural networks were then compared against traditional multivariate analysis on 10 randomly selected validation data sets.

The ANN's achieved a mean sensitivity of 0.62, range 0.61-0.65 and a specificity of 0.77, range 0.73-0.79 with a mean overall accuracy of 0.71 (95%C.I. 0.57-0.83) as opposed to the traditional multivariate analysis which achieved a mean sensitivity of 0.56, range 0.43-0.79 and specificity of 0.68, range 0.52-0.85 with a mean overall accuracy of 0.62 (95%C.I. 0.49-0.75).

The failure to improve on the accuracy of the traditional linear statistical methods may be due to either too small a learning set, or mathematically too simple a problem. This accepted, in practical terms, ANN's provide a more consistent method of prediction of outcome gastric following gastric cancer surgery.

### O64 PANCREATIC CANCER DEATHS IN SURGICAL CARE

M A Jahan, F Marjoribanks, S J Nixon, A M Thompson, Department of Surgery, Ninewells Hospital & Medical School, Dundee, Scotland

*Aims and methods* -The Scottish Audit of Surgical Mortality (SASM) collects data on the management of all patients who die in surgical wards in Scotland. The aim of this study was to identify criteria and standards for future audit of death from pancreatic cancer. *Results*- During the first 3 years of the SASM (1994-1996 inclusive), there were 334 deaths in surgical wards out of 1650 total deaths from pancreatic cancer in Scotland; of those admitted from the community 165 (75%) were emergency admissions, 134 (40%) had an endoscopic, surgical, or a radiological procedure. In 147 (44%) patients the treatment was limited and 94 (28%) had terminal care. Seventy (53%) operations were performed as urgent or emergency, 18 (5.4%) had an open and shut laparotomy, and 8 (2.4%) deaths followed pancreatic resection. In 21 (21/134) patients there were intraoperative technical problems and 25 (25/134) patients required a second procedure. Adverse factors in surgical management were identified in 16 (16/134) patients and of anaesthetic management in 6 (5%) patients. The decision to operate and the choice of operation could have been improved in 15 (15/134) patients. Thirteen (17.5%) patients in whom HDU/ITU was desiarable were denied access. One in five deaths were associated with potentially avoidable or treatable co-morbidity. The cause of death in 25% of cases were potentially avoidable and treatable. *Conclusions*- Based on the result of this study, patients with pancreatic cancer should be admitted electively to hospitals with adequate facility for modern staging techniques, management, and peri-operative monitoring facilities to reduce the unacceptably high premature mortality observed in this study.

### O65

**PERCUTANEOUS NEEDLE PHOTODYNAMIC THERAPY OF PANCREATIC TUMOURS**

W R Lees, A R Gillams, D Whitelaw, A Hatfield, S G Bown. Department of Medical Imaging, The Middlesex Hospital, Mortimer Street, London, UK.

**PURPOSE:** Simple and effective methods of debulking small pancreatic tumours do not presently exist. PDT offers the potential of destruction of significant volumes of tumour tissue within the pancreas using fine needles to deliver laser fibres.

**METHOD:** Seven patients were treated over a 16m period using 4-6 19g needles delivered into the head of the pancreas with a 17 mm spacing 5 days after sensitization with the agent MTHPC. Twenty joules of energy from a diode laser was delivered at each treatment station. All seven patients had stents decompressing the previously obstructed biliary tree. A combination of CT and ultrasound guidance was used to guide needle delivery. The effectiveness of treatment was assessed at five days with dynamic contrast enhanced CT where devascularisation of the pancreas was seen as zero enhancement. Follow-up scans were performed at 1m, 3m, 6m and 12m.

**RESULTS:** All 7 patients tolerated the procedure well but experienced some systemic disturbance following the procedure in the first 3 days. Around each treatment station, the devascularised region was 13-18 mm in diameter and the total volume of necrosis was 12-36 mls. In three cases, ulceration of the medial wall of the duodenum was noted on endoscopy at five days. This was not clinically significant in any of the patients and had healed by three months. All seven patients remain alive. (Range 2-16m, median of 7m from treatment.)

**CONCLUSION:** Percutaneous photodynamic therapy appears a safe and well-tolerated method of debulking pancreatic tumours. It's impact on survival should emerge rapidly from a larger series with longer follow-up.

### O66

**A PILOT STUDY OF SPHINCTER-SPARING SURGERY, ENDOLUMINAL & EXTERNAL BEAM RADIOTHERAPY IN RECTAL CANCER**

AC McDonald*[1], M Hershmann[2], C Makin[3] and S Myint[1].

[1]Clatterbridge Centre for Oncology, and Departments of Surgery, [2]Royal Liverpool and [3]Arrowe Park Hospitals, Merseyside, UK

Colostomy is an inevitable accompaniment to abdomino-perineal resection (APR). Between 1991-7, 38 selected patients with well or moderately differentiated T1 (16pts) or T2 (22pts) cancers of the distal rectum (36pts < 8cm from anal margin) were treated radically using anal sphincter–preserving techniques. Most patients were elderly (median age 74, range 42-89 yrs), many (14pts) with concomitant medical problems precluding APR. Twenty-two pts received external beam radiotherapy (EBRT) before (15pts) or after (7pts) trans-anal tumour resection (TART, utilising trans-anal endoscopic microsurgery in 12pts). Three pts underwent trans-anal tumour resection and contact radiation boost using the Papillon technique. Thirteen pts were treated using only non-surgical techniques; 7pts received radical contact radiotherapy and 3 further pts received initial EBRT and a local contact boost. Concomitant infusional 5-FU (750mg-1g/m$^2$/day x 4, weeks 1 & 4) was given with EBRT in selected pts of good performance status (6pts).

Treatment was well tolerated and toxicities generally mild (≤ RTOG gd 2). Three pts failed to respond fully to initial non-surgical treatment and a further 3pts have recurred locally after 4-9 months. Each has undergone surgical salvage (5=APR; 1=TART). A further patient developed metastatic disease after 3 yrs; median follow up period is 24 months (range = 4-64 months) and sphincter preservation was achieved in 33pts (87%). This is a highly selected series, however within this group the sphincter-conserving techniques adopted are well tolerated and permit good rates of local control; this approach merits evaluation in further, randomised, studies.

### O67

**NEOADJUVANT CHEMORADIOTHERAPY IN CARCINOMA OF THE OESOPHAGUS.**

E.W.Toy,T.D.Crosby,D.J.Bowrey, G.W.Clark, P.D.Carey,T.S.Maughan, Cardiff, U.K.

Long term outcome in carcinoma of the oesophagus is poor. Recently Walsh et al (1996) and Urba et al (1997) have reported improved results following combined modality neo-adjuvant treatment.

17 patients, mean age 61 yrs (46-73)with biopsy proven carcinoma of the oesophagus(10 adenocarcinoma, 7 squamous cell) were treated with neo-adjuvant chemoradiotherapy after staging with CT and endoluminal ultrasound. It was aimed to give 40 Gy in 15 fractions via a 2 phase technique (30 Gy in first 3 patients) with 2 cycles of Cisplatin 80 mg/m2 day 1 and 5-FU 1g/ m2 days 2-5 q 28 d, the first cycle concurrent with RT. Restaging was performed prior to surgery.

All received 100% of the prescribed radiation dose. 2 patients required a treatment break due to toxicity.14 patients received 100% doses of chemotherapy.2 had dose reductions.1 declined a second cycle.3 required a delay prior to cycle 2. 8 (47%) of patients experienced toxicities of grade 3 or 4,(myelosupression 29%, diarrhoea 12%,dysphagia 6%, vomiting 6%, mucositis 6%) 1 patient died prior to surgery. 15 patients proceeded to surgery at a median time of 41 days post chemotherapy(15-101). 4 died post-operatively from serious respiratory complications. Dysphagia scores pre-operatively were improved in 43% and worse in 31%, but this did not correlate with tumour response. EUS was repeated in 12 cases pre-operatively suggesting 25% downstaging. 17% progression on TNM criteria. Comparison of initial staging with pathological staging was possible in 14 patients and revealed : 64% downstaged, 29% progression and 7% stable disease following chemoradiation. 4 (29%) had a complete pathological response. Subgroup analysis of response shows that squamous carcinomas respond better to this regimen than adenocarcinomas( p= 0.04 Fishers exact test). A Kaplan-Meier crude survival curve shows a 61% probability of one year survival (median follow up of survivors, 61 weeks )

**Conclusions** : Use of the dysphagia score and EUS are poor in determining tumour staging post chemoradiation. The response of the squamous cell tumours is encouraging, whilst that of the adenocarcinomas is particularly disappointing. It suggests that different regimens may be indicated for the two pathologies. It is too early for a survival improvement to be confirmed but any benefits were at the cost of significant toxicity. Whilst neo-adjuvant chemoradiation may improve survival the optimal regimen remains to be defined.

### O68

**SOCIOECONOMIC STATUS AND STAGE AT PRESENTATION IN COLORECTAL CANCER,**

MV Ionescu, IS Tait, RJC Steele, Dept. of Surgery, Ninewells Hospital and Medical School, Dundee DD1 9SY, UK

**Introduction:** Early diagnosis of colorectal cancer (CRC) is important and several studies have shown wide variation in stage at presentation by district of residence. The reasons for this are not clear and this study has evaluated Dukes' stage at presentation relative to socioeconomic status, in a stable population (Tayside, Scotland).

**Methods:** 777 consecutive patients underwent resectional surgery for CRC from 1991 to 1997. Dukes' stage was documented and socioeconomic status rated by Carstairs Score (CS), a Deprivation Index based on Postal Code. Patients were stratified into Groups 1-4 by quartiles, according to CS, with group 4 having the highest score (i.e. the highest deprivation). Dukes' stage between groups was analysed by: $\chi^2$ test, $\chi^2$ for trend and odds ratio (OR).

**Results:** The mean age was 69.5 years (median 71 years; range 30-95 years) and the male:female ratio was 1:1.12. The proportion of patients with Dukes' stage A tumours was lowest in Group 4 (Table) ($p<0.01$ $\chi^2$ test; $p<0.005$ $\chi^2$ test for trend). In group 4, OR for Dukes' A was 0.574 [95% confidence interval (CI) 0.38;0.85], and OR for Dukes' B or C was 1.74 [95% CI 1.17;2.58].

| Groups | n | Carstairs Range | Stage A n (%) | Stage B n (%) | Stage C n (%) |
|---|---|---|---|---|---|
| Group 1 | 171 | -5.44;-3.55 | 31 (18) | 70 (41) | 70 (41) |
| Group 2 | 205 | -3.55;-1.23 | 41 (20) | 87 (42) | 77 (38) |
| Group 3 | 199 | -1.23; 1.44 | 29 (15) | 84 (42) | 86 (43) |
| Group 4 | 202 | 1.44; 5.97 | 19 (9) | 89 (44) | 94 (47) |

**Conclusions:** Patients from less affluent backgrounds tend to present at a more advanced Dukes' stage of CRC. This may explain regional variations and has implications for public awareness strategy.

**PREOPERATIVE CHEMORADIOTHERAPY FOR LOCALLY ADVANCED RECTAL CANCER**

**J I Geh, A Makris , R Ashford, E Grosch, R Glynne-Jones; Mount Vernon Hospital, Northwood, UK.**

**Introduction:** Concurrent chemoradiotherapy (CRT) in unresectable rectal cancer has been reported to result in high response rates. We report the compliance, toxicity and pathological response rate when CRT is given preoperatively.

**Methods:** 62 patients with locally advanced borderline resectable or unresectable (fixed 47; tethered 9; bulky mobile 6) adenocarcinoma of the rectum (median age 64 years, range 42-83) were treated preoperatively with CRT. Radiotherapy (RT) was given using a 3 field plan; 45 Gy in 25 fractions over 33 days (4 patients received 50-53 Gy in 25-29 fractions). 52 patients received folinic acid 20 mg/m$^2$ iv bolus followed by 5-fluorouracil (5-FU) 350 mg/m$^2$ iv one hour before RT on days 1-5 and 29-33. 10 patients received a continuous 5-FU infusion 200 mg/m$^2$ via a Hickman line for 12 weeks commencing 1-2 weeks prior to commencement of radiotherapy. Patients were reassessed with the referring surgeon at 4 weeks and operation planned at 6-12 weeks.

**Results:** Total compliance of CRT was achieved in 57 patients (92%). Two patients required interruption of RT for 4 and 7 days for severe diarrhoea. Two patients needed removal of infected Hickman line. WHO grade 4 toxicity occurred in 4 patients (6%); diarrhoea 2, skin 1, neutropenic sepsis 1, all of whom required admission to hospital. There were no CRT related deaths. In addition, 12 patients experienced WHO grade 3 toxicity (moist desquamation) of perineal skin and 9 patients had other WHO grade 3 toxicities (diarrhoea 4, tenesmus 1, urinary frequency 4, vomiting 1). To date, 44 patients have proceeded to operation; 16 anterior resection, 22 anterior perineal resection and 6 were found to be unresectable (3 intra-abdominal metastases; 3 fixed tumours). Histology is available in all 38 patients who had an attempted curative resection - pathological CR in 6 (16%); Dukes' A in 4 (11%); Dukes' B in 19 (50%); Dukes C in 9 (23%). Only 3 patients had involved resection margins. Median duration from end of CRT to surgery was 58 days (range 20-137 days).

| | Fixed | Tethered | Mobile |
|---|---|---|---|
| pCR | 5 | 1 | 0 |
| Dukes' A | 4 | 0 | 0 |
| Dukes' B | 11 | 5 | 3 |
| Dukes' C | 7 | 1 | 1 |

After a median follow up interval of 12 months (range 1-46 months), 12 patients have died (progressive/metastatic disease 8; myocardial infarct 2; postoperative complications 2). Of those who had anterior or AP resection, only 2 patients (5%) have had local recurrence. 3 patients have relapsed with distant metastases.

**Conclusions:** Preoperative CRT using using the above regimes is associated with high compliance, acceptable toxicity, good resectability and pathological downstaging in a group of patients with advanced rectal carcinoma. The early results demonstrate a low local recurrence rate. Data on surgical morbidity and late functional results/toxicity will be available shortly.

### O69 PATTERNS OF BREAST RELAPSE FOLLOWING BREAST CONSERVING SURGERY AND RADIOTHERAPY.

B.Magee, E.A.Young, R.Swindell Christie Hospital, Manchester UK

The pattern of breast relapse has been studied in a prospective randomised controlled trial of 2 radiotherapy techniques following breast conserving surgery in clinical stage I breast cancers. 708 patients were randomised to either whole breast radiotherapy using megavoltage tangential fields (WB group) OR a localised electron field directed at the tumour bed (LF group)

The WB group has suffered fewer breast relapses than the LF group; for invasive ductal carcinomas:WB: 31 breast relapses (10%); LF: 51 breast relapses (16%); invasive lobular carcinomas: WB: 3 breast relapses (10%): LF: 14 breast relapses (38%)

This reduction in breast relapses for the WB group occurs both as a reduction in relapses in or close to the same quadrant as the primary tumour e.g. invasive ductal carcinomas: WB: 18(5.5%), LF: 31(10%) and also in fewer recurrences elsewhere in the breast: WB: 5(1.5%), LF: 18(5.5%)

This suggests that the improvement with whole breast radiotherapy is due to 2 main factors: firstly a reduction in geographical misses compared to the electron field (LF) group and secondly an effect of whole breast radiotherapy on multicentre foci of disease distant from the primary tumour.

### O70 QUANTITATION OF C-*ERB*B-2 IN PRIMARY BREAST CANCER: GENE AMPLIFICATION IS NOT THE WHOLE PICTURE.

D. Chong, J. Reeves, T. Cooke, [1]W.D. George, [1]E. Mallon, [2]B. Ozanne and P. Stanton. University Dept of Surgery, Glasgow Royal Infirmary, [1]University Dept of Surgery, Western Infirmary, Glasgow and [2]Beatson Institute for Cancer Research, University of Glasgow

C-*erb*B-2 amplification or high levels of overexpression are found in about 25% of breast cancers and these patients have a poor outcome. Using a quantitative radioimmunohistochemical method in frozen sections we have previously shown that c-*erb*B-2 is nearly always overexpressed in primary breast cancers with gene amplification accounting for a population with very high levels of expression.

Here we report on the application of radioimmunohistochemistry to measure the c-*erb*B-2 protein in a larger set of cases (n = 182) with followup exceeding 5 years. Disease specific survival was assessed using Kaplan Meier life table analysis and log rank tests.

Disease specific survival and c-*erb*B-2 expression

88% of tumours overexpressed c-*erb*B-2. 23% had greater than 20 times normal expression, which we have previously shown, indicates amplification at this locus. These cases had a significantly poorer survival than the rest of the overexpressors ($p<0.0001$). 12% of cases had lower c-*erb*B-2 expression compared to normal breast tissue. These patients also had a poorer prognosis than the non-amplified overexpressors ($p<0.0001$)

Quantitative estimations of c-*erb*B-2 in frozen sections allow measurements of the protein in nearly all cases whereas paraffin section immunohistochemistry provides subjective data on only the highest expressors. Tumours with down regulated c-*erb*B-2 have as poor a prognosis as those with gene amplification levels of the protein.

### O71 EGFR AND c-*erb*B-2 EXPRESSION IN BREAST CANCER PROGRESSION.

D. Chong, J. Reeves, T. Cooke, [1]W.D. George, [1]E. Mallon, [2]B. Ozanne and P. Stanton, University Department of Surgery, Glasgow Royal Infirmary, [1]University Department of Surgery, Western Infirmary, Glasgow and [2]Beatson Institute for Cancer Research, University of Glasgow.

EGFR and c-erbB-2 are being explored as therapeutic targets in breast cancer. This approach will be enhanced if it is effective in pre-invasive as well as invasive disease. We have found that EGFR is downregulated and c-erbB-2 is overexpressed in 95% of invasive breast cancers, but little is known about the role of these factors in *in situ* disease.

In frozen sections of invasive cancers with evidence of ductal carcinoma *in-situ* (DCIS), EGFR (n=46) and c-*erb*B-2 (n=40) receptor levels were assayed quantitatively using a radiolabelled antibody method. Numbers of receptors were determined by comparison with cell lines of known receptor density and compared in each element of the tumour.

EGFR and c-*erb*B-2 expression each varied by a factor of several thousand. The frequency distributions for expression of both factors were comparable in DCIS and invasive tumours (Mann-Whitney U test, EGFR p=0.41, c-erbB-2 p=0.56). Within each tumour, there was no significant difference in expression of c-erbB-2 or EGFR in the DCIS and invasive components (Wilcoxon Signed Rank Test, EGFR p=0.419, c-*erb*B-2 p=0.343).

These data suggest that alterations in type I growth factor receptors occur before progression of *in-situ* disease to invasive cancer. High levels of c-*erb*B-2 overexpression in both *in-situ* and invasive areas suggest that the c-*erb*B-2 product is a potential therapeutic target for the treatment of breast cancer at an early stage.

### O72 MRI IN THE ROUTINE THREE-DIMENSIONAL PLANNING OF BREAST CANCER.

E.C. Whipp, R. Hartley-Davies, S. Dunne, A. Gee, M. Keen and M. Halliwell. United Bristol Healthcare Trust, Bristol BS1 6SY, UK.

**Objective**: To determine the accuracy of conventional breast plans using images from an open MRI scanner.

**Methods**: Having resolved the distortion problems inherent in MRI by using software written within our department, we have been able to feed MRI images made in several planes directly into our planning system.

**Results**: After performing 30 MRI scans of breast, chest wall and cervico-axillary chain, also imaging the heart, limits of breast tissue, chest wall thickness, tumour excision sites and the position of internal mammary nodes in breast cancer patients in the treatment position using an open MRI scanner, we found nearly 50% of conventional plans had to be amended. As a result, we now perform routine MRI before the planning session whenever possible.

**Conclusions:** 10% of tumour cavities were missed by tangential fields. Post-operative tumour cavities were difficult to locate and bolus distorts the breast and moves these cavities further. The territory of the cervico-axillary chain is often wider than expected and the three-dimensional anatomy of patients in this part of the body varies tremendously.

### O73 ROLE OF *BRCA2* IN DNA REPAIR: IMPLICATIONS FOR BREAST CARCINOGENESIS AND THERAPY.

**Gillian Ross; A. Tutt; D.Bertwhistle[1]; A.Ashworth[1]** Academic Radiotherapy and Oncology Unit; [1] Section of Gene Function and Regulation, Chester Beatty Labs, Institute of Cancer Research,London.

Germline mutations in the *BRCA1* and *BRCA2* genes may account for a significant proportion of the 5% of early onset cases of breast cancer.An increasing body of evidence suggests that these cancer predisposition genes may have a critiial function in both DNA repair and maintenance of genomic stability.

We have assessed DNA repair in fibroblasts derived from knockout mice of genotype Brca 2+/+.+/-,and -/-; animals were generated by siting an insertional mutation in exon 11 giving rise to a truncated protein, by homologous recombination and standard ES technology. The phenotype includes increased risk of embryonic lethality, skeletal growth defects, failure of germ cell maturation, and early onset of thymic lymphoma. Cells fail to proliferate *in vitro*, associated with overexpression of *p53* and *p21/WAF1*. We hypothesised that the observed growth arrest and activation of genes involved in DNA damage signal transduction might imply an upstream function for BRCA2 in DNA repair. Using neutral cell gel electrophoresis, we have demonstrated a defect in DNA double strand break rejoining *in vitro*, in fibroblasts derived from *Brca2-/-* animals after ionising radiation. This data is the first direct biochemical evidence of a role for BRCA2 in DNA repair. Continuing our genetic/biochemical approach,we will use the Brca 2 +/- genotype as a model of the human germline state to investigate interaction with other genes implicated in breast carcinogenesis (*p53,AT*) and cross breed to a transgenic mutational reporter strain(Mutamouse) to investigate enviromental factors influencing mutability and carcinogenesis, and help design intervention strategies relevent to the clinic.

### O74 PROSPECTIVE RANDOMIZED STUDY OF MITOXANTRONE (M) AND VINORELBINE (V) VS FAC OR FEC IN ABC.

M. Namer, F. Turpin, D. Serin, G. Ganem, G. Calais, P. Maillart, P. Colin, G. Prévost, P. Romestaing, JD. Tigaut, P. Clavère, Nice - France

The combination of Fluorouracil(F), Cytoxan(C), with either Adriamycin(A) or Epirubicin(E) (FAC/FEC) is considered as gold standard regimen in ABC.We performed a randomized trial comparing this regimen (F: 500 mg/m², A or E: 50 mg/m², C: 500 mg/m² on day 1) to the combination M.V. (M: 12 mg/m², V: 25 mg/m² on day 1 and V: 25 mg/m² on day 8 if neutrophils ≥ 1000/mm³). Treatment cycles were repeated every 21 days. Stratification was based upon prior adjuvant CT. 281 patients (pts) were randomized between FAC/FEC (139 pts) and MV (142 pts). 92 pts had received prior adjuvant CT (77 with anthracyclin). 82 % of pts had visceral metastases and the median number of metastatic sites was 2 (1-7). Overall 841 FAC/FEC cycles and 698 MV cycles were given (median/pt: 6 and 5 respectively). The mean relative dose intensity was 95 %, 96 %, 96 % for FAC/FEC and 92 %, 77 % for MV. Haematological toxicity delayed treatment cycles in 17 % (FAC/FEC) and 27 % (MV) and let to withdraw V on day 8 in 29 %. Febrile neutropenia required hospitalisation in 2 % (FAC/FEC) and 15 % (MV) of pts (p=0.001) and antibiotics in 0.6 % and 6 % of pts. Non haematological toxicity was in favor of MV for grades 3 and 4 nausea and vomiting (N/V) (16 % vs 8 %: p = 0.03) and for grade 3 alopecia (30 % vs 7 %: p = 0.0001). Mostly minor cardiac events occured in 19 pts (10 FAC/FEC, 9 MV). The objective response rate (ORR) of the whole population was similar: FAC/FEC: 33.3 % vs MV: 35.5 % (p = 0.014). For pts who have received prior adjuvant CT the ORR was higher in MV arm (33 % vs 13 %: p = 0.025). For those who had not there is a slight non statistically significant trend in favor of FAC/FEC arm (43 % vs 35 %: p = 0.25). Median duration of response was 10 months (1-24) for FAC/FEC and 7 months (1-27) for MV. Time to progression (TTP) and overall survival (OS) were not different in the two groups (p = 0.79 and p = 0.27 respectively) but showed the same divergence when prior adjuvant therapy was taken into account. When used as first line treatment in pts with metastatic breast cancer, MV and FAC/FEC have no different efficacy in term of ORR, TTP and OS for the whole population. However, for pts with previous adjuvant CT, MV combination seems to be superior. Haematological toxicity was higher for this regimen while non haematological toxicity (N/V, alopecia) was less frequent.

### O75 PROGNOSTIC VALUE OF TUMOUR MARKERS IN PATIENTS WITH AXILLARY NODE NEGATIVE BREAST CANCERS.

Y.S. Wang[1], Q. Liu[1], X.P. Hao[1], W.G. Jiang[2], R.E. Mansel[2]. [1]Shangdong Cancer Hospital and Institute, Shangdon, China, and [2]University Department of Surgery, University of Wales College of Medicine, Cardiff, UK.

This study examined the prognostic value of a range of tumour markers in patients with node negative breast cancer.

Tissues from patients with breast cancer (n=116) were studied retrospectively. The age and menopausal status of the patients, tumour size, type and grades, oestrogen receptor (ER) (immunohistochemistry, IHC), DNA ploidy (flow cytometry, FC), S phase fraction (SPF) (FC), proliferative index (PI) (FC), cathepsin D (IHC), mutant p53 (IHC), CD44v (FC), *c-erb*B2 (IHC), *ras* p21 (FC) were assessed and correlated with 10 year overall survival. CD44v, p53, cathepsin D, as well as the size, histopathological type, nuclear (NG)/histological (HG) grades, ER, SPF and PI were found to have a univariant prognostic relationship with survival. A prognostic index was obtained with the Cox regression model: index=0.075×size+0.196×NG+0.146 ×ER+0.059×SPF+0.241× cath D+0.270×p53+0.127×CD44v. These 7 parameters were found to be independent prognostic factors for survival. Patients were divided into three groups according to the prognostic index thus calculated: i. index<1.6 (n=60): 10 yr survival 91.7%; ii. 1.6≤index≤1.9 (n=24): 10 yr survival 58.3%; iii. index>1.9 (n=32): 10 yr survival 15.6%, compared with 10 yr overall survival 63.8% of all the patients in this study.

It is concluded that tumour markers p53, CD44v, and cathepsin D as well as tumour size, nuclear grade, ER, and SPF can be used as markers for the prognosis of patients with lymph node negative breast cancer.